# Neuroligin 2 regulates absence seizures and behavioral arrests through GABAergic transmission within the thalamocortical circuitry

**DOI:** 10.1038/s41467-020-17560-3

**Published:** 2020-07-27

**Authors:** Feng Cao, Jackie J. Liu, Susan Zhou, Miguel A. Cortez, O. Carter Snead, Jing Han, Zhengping Jia

**Affiliations:** 1 Neuroscience & Mental Health, The Hospital for Sick Children, Toronto, ON M5G 1X8 Canada; 2 Department of Physiology, University of Toronto, Toronto, ON M5S 1A8 Canada; 3 Department of Pediatrics, University of Toronto, Toronto, ON M5G 1X8 Canada; 4 MOE Key Laboratory of Modern Teaching Technology, Shaanxi Normal University, 710062 Xi’an, Shaanxi China

**Keywords:** Neuroscience, Diseases of the nervous system, Molecular neuroscience, Synaptic transmission

## Abstract

Epilepsy and autism spectrum disorders (ASD) are two distinct brain disorders but have a high rate of co-occurrence, suggesting shared pathogenic mechanisms. Neuroligins are cell adhesion molecules important in synaptic function and ASD, but their role in epilepsy remains unknown. In this study, we show that Neuroligin 2 (NLG2) knockout mice exhibit abnormal spike and wave discharges (SWDs) and behavioral arrests characteristic of absence seizures. The anti-absence seizure drug ethosuximide blocks SWDs and rescues behavioral arrests and social memory impairment in the knockout mice. Restoring GABAergic transmission either by optogenetic activation of the thalamic reticular nucleus (nRT) presynaptic terminals or postsynaptic NLG2 expression in the thalamic neurons reduces the SWDs and behavioral arrests in the knockout mice. These results indicate that NLG2-mediated GABAergic transmission at the nRT-thalamic circuit represents a common mechanism underlying both epileptic seizures and ASD.

## Abstract (web summary)

Neuroligins are postsynaptic cell adhesion molecules that are involved in synapse function and autism spectrum disorder. The authors show that NLG2-mediated GABAergic transmission at the thalamic reticular nucleus-thalamic circuit is a common mechanism underlying epileptic seizures and ASD.

## Introduction

Autism spectrum disorders (ASD) are a class of life-long neurodevelopmental disorders characterized by deficits in social behavior and communication, repetitive behaviors, and restricted interests^1–3^. In addition to these symptoms, ASD also exhibit high rates of comorbidity with other abnormalities such as increased seizures, depression, anxiety, and sleep problems^4–8^. The prevalence of epileptic seizures in autistic individuals is particularly striking^9^. Clinically apparent seizures occur in 1/3 of ASD patients, ranging from 5–46%, and ~32% of individuals with epilepsy suffer from ASD^10^. This co-occurrence suggests potentially shared mechanisms underlying these two disorders. Indeed, a decrease of GABA transmission has been found in both animal models of ASD and epilepsy, and altered excitation to inhibition (E/I) balance may underlie both disorders^11–18^. However, a molecular link between ASD and epileptic seizures remains elusive.

Neuroligins (NLGs) are a family of postsynaptic cell adhesion proteins known to be critically involved in synapse formation, maturation, and function^14,19,20^. Genetic mutations in the NLG genes are closely linked with ASD and other neurodevelopmental disorders^14,19–23^. Animal models of NLGs also display autistic-like behaviors including impaired social interaction, vocal communication, and cognition^24–26^. These results indicate that abnormal NLG function is a causal factor for ASD phenotypes, but whether dysregulated NLGs are also involved in the pathogenic process of epileptic seizures remains unclear.

Several studies suggest that Neuroligin 2 (NLG2) may be particularly important in linking ASD with increased seizures. First, NLG2 is a potent regulator of the E/I balance; loss of NLG2 dramatically reduces GABA receptors and GABA-mediated synaptic transmission^14,27–31^, resulting in increased neuronal excitability^32^. Second, global overexpression of NLG2 in transgenic mice leads to motor discoordination, social impairment and abnormal electroencephalographic (EEG) activities, characteristics shared by ASD and epilepsy^33^. Third, genetic mutations in the NLG2 gene are associated with human patients with multiple symptoms including anxiety, obsessive-compulsive behaviors, developmental delay and autism^14,22,23^. However, there is no direct evidence to support the specific involvement of NLG2 in epileptic seizures. In addition, the underlying mechanisms and circuits involved are unknown.

In this study, we find that NLG2 knockout (KO) mice exhibit abnormal EEG activity and behavioral arrests reminiscent of absence seizures. We further demonstrate that these seizures and behavioral deficits are associated with impaired GABAergic transmission in the thalamic neurons and can be alleviated by activating the thalamic reticular nucleus (nRT) projection to the ventrobasal thalamus. Thus, NLG2-mediated GABAergic regulation at the nRT-thalamic pathway represents a key molecular mechanism to link epileptic seizures to ASD.

## Results

### NLG2 KO mice exhibit SWDs and behavior arrests

**Fig. 1:**
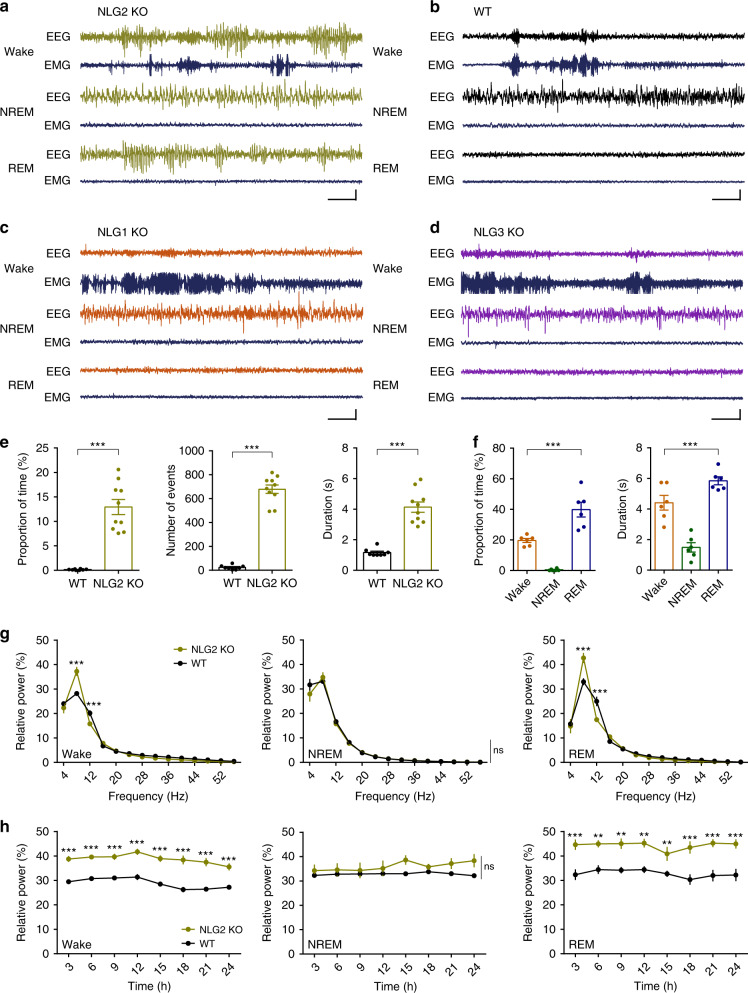
NLG2 KO mice exhibit spontaneous SWDs and behavior arrests. **a**–**d** Representative EEG and EMG traces showing SWDs in NLG2 KO (**a**), but not in WT (**b**), NLG1 KO (**c**), and NLG3 KO mice (**d**). Scale bars: 2 s/100 μV. **e** Averaged EEG recordings (3 h light period and 3 h dark period) showing increased SWD proportion (*p* < 0.0001), number (*p* < 0.0001), and duration (*p* < 0.0001) in NLG2 KO (*n* = 10) compared to WT mice (*n* = 8). Two-sided *t*-test. **f** Averaged EEG recordings (3 h light period and 3 h dark period) showing predominant presence of SWDs in the wake and REM sleep in NLG2 KO mice (proportion of time: *p* < 0.0001, *n* = 6; duration: *p* < 0.0001, *n* = 6; one-way ANOVA). **g** Relative power of various EEG frequencies showing significant genotype-frequency interactions (repeated two-way ANOVA) for the wake (*p* = 0.0001, WT: *n* = 6, NLG2 KO: *n* = 8, post-hoc Holm–Sidak’s comparisons: *p* < 0.0001 for the 5–8 Hz bin, *p* = 0.0004 for the 9–12 Hz bin) and REM sleep (*p* < 0.0001, WT: *n* = 6, NLG2 KO: *n* = 7, post-hoc Holm–Sidak’s comparisons: *p* < 0.0001 for the 5–8 Hz bin, *p* < 0.0001 for the 9–12 Hz bin), but not for the NREM sleep (*p* = 0.7788, WT: *n* = 6, NLG2 KO: *n* = 8). **h** Relative power of theta activity (5–8 Hz bin) over the 24 h EEG recordings showing significant genotype effect (repeated two-way ANOVA) for the wake (*p* < 0.0001, WT: *n* = 6, NLG2 KO: *n* = 7, post-hoc Holm–Sidak’s comparisons: significant at each time point) and REM sleep (*p* = 0.0003, WT: *n* = 5, NLG2 KO: *n* = 6, post-hoc Holm–Sidak’s comparisons: significant at each time point), but not for the NREM sleep (*p* = 0.0897, WT: *n* = 5, NLG2 KO: *n* = 7). Data in **e**–**h** represent mean ± SEM. **p < 0.01, ***p < 0.001. ns nonsignificant. Source data are provided as a Source Data file.

**Fig. 2:**
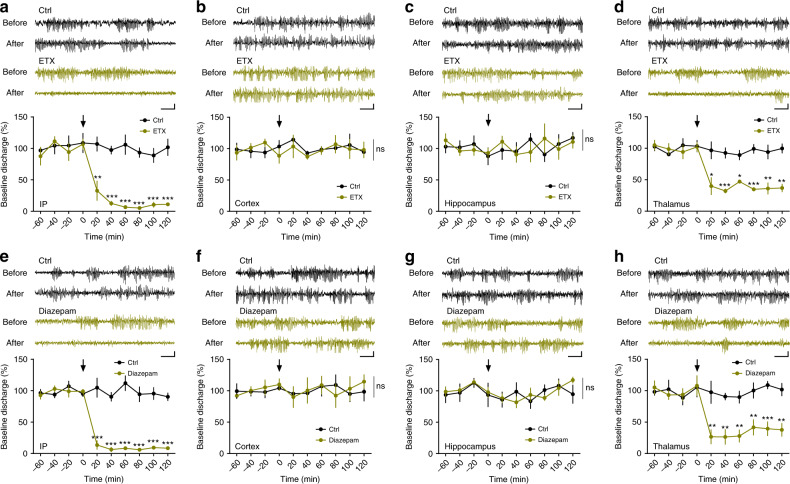
Absence seizure in NLG2 KO mice involves GABAergic transmission in the thalamus. **a** Sample traces of EEG recordings and summary graph showing significant cessation of SWDs in NLG2 KO mice with systemic injection of ETX (200 mg/kg) compared to control (Ctrl, 0.3% tween 80) (*p* < 0.0001, ETX: *n* = 8, Ctrl: *n* = 8, significant difference at each post-injection time point). **b**–**d** Sample traces of EEG recordings and summary graph of SWDs in NLG2 KO mice locally injected with either 1 μl 100 mg/ml ETX or 0.3% tween 80 control solvent (Ctrl) to the cortex (**b**), hippocampus (**c**), or thalamus (**d**) showing significant cessation of SWDs in thalamus injected mice (*p* < 0.0001, ETX: *n* = 5, Ctrl: *n* = 4, significant difference at each post-injection time point), but not in cortex (*p* = 0.5226, ETX: *n* = 5, Ctrl: *n* = 8) or hippocampus (*p* = 0.7673, ETX: *n* = 7, Ctrl: *n* = 7) injected mice. **e** Sample traces and summary graph showing significant cessation of SWDs in NLG2 KO mice systemically injected with 1 mg/kg diazepam compared to saline (Ctrl) (*p* < 0.0001, Diazepam: *n* = 6, Ctrl: *n* = 7, significant difference at each post-injection time point). **f**–**h** Sample traces and summary graphs of SWDs in NLG2 KO mice locally injected with either 1 μl 5 mg/ml diazepam or saline (Ctrl) to the cortex (**f**), hippocampus (**g**), or thalamus (**h**) showing significant cessation of SWDs in the thalamus injected mice (*p* < 0.0001, Diazepam: *n* = 6, Ctrl: *n* = 7, significant difference at each post-injection time point), but not in the cortex (*p* = 0.9265, Diazepam: *n* = 6, Ctrl: *n* = 8) or hippocampus (*p* = 0.5791, Diazepam: *n* = 5, Ctrl: *n* = 7) injected mice. Arrow indicates the time for injection. Scale bars: 3 s/100 μV. Data in **a**–**h** represent mean ± SEM. ns nonsignificant. *p < 0.05, **p < 0.01, ***p < 0.001 with two-sided *t*-test. Source data are provided as a Source Data file.

To determine whether NLGs are involved in epileptic seizures, we performed cortical EEG recordings in freely moving Neuroligin 1 (NLG1) KO, NLG2 KO, Neuroligin 3 (NLG3) KO mice and their wild-type (WT) littermates. The motor activity of the mice was monitored by simultaneous electromyographic (EMG) recordings. These recordings were maintained continuously for 48 h allowing for the assessment of EEG activities in all three behavior states (i.e., wake, NREM sleep, and REM sleep). As shown in Fig. 1a, the NLG2 KO mice exhibited spontaneous spike-wave discharges (SWDs) that were associated with behavioral arrest episodes (Supplementary Fig. 1). The SWDs and behavioral arrests were not observed in WT, NLG1 KO, or NLG3 KO mice, indicating these phenotypes were specific to NLG2 KO mice (Fig. 1b–d, Supplementary Fig. 1). The SWDs occurred regularly and frequently accounting for ~13% of the entire 48 h recording (i.e., 1 episode of SWD every ~30 s), mainly from 5–8 Hz with a mean frequency of 5.7016 ± 0.1189 Hz and each episode lasting average 4.1320 ± 0.3358 s (Fig. 1e). Interestingly, the SWDs were predominantly observed during the wake and REM sleep, but not during NREM sleep (Fig. 1f). The power-spectral analysis showed that NLG2 KO mice exhibited significantly increased strength in the theta range (5–8 Hz) during wake and REM sleep but not during NREM sleep (Fig. 1g). The increased theta activity in wake and REM sleep in NLG2 KO mice persisted over a 24 h period (Fig. 1h, average of 2-day recordings). These recurrent SWDs (e.g., 5–8 Hz accompanied by behavioral arrests) are consistent with absence seizures. To confirm this, we systemically (i.e., intraperitoneally—IP) injected ethosuximide (ETX), a highly specific anti-absence seizure drug, to the KO mice. ETX abolished SWDs in all tested mice whereas control vehicle had no effect (Fig. 2a). Taken together, these results indicate that the deletion of NLG2 causes absence seizure-like SWDs in mice.

### NLG2 KO mice are impaired in thalamic GABAergic transmission

**Fig. 3:**
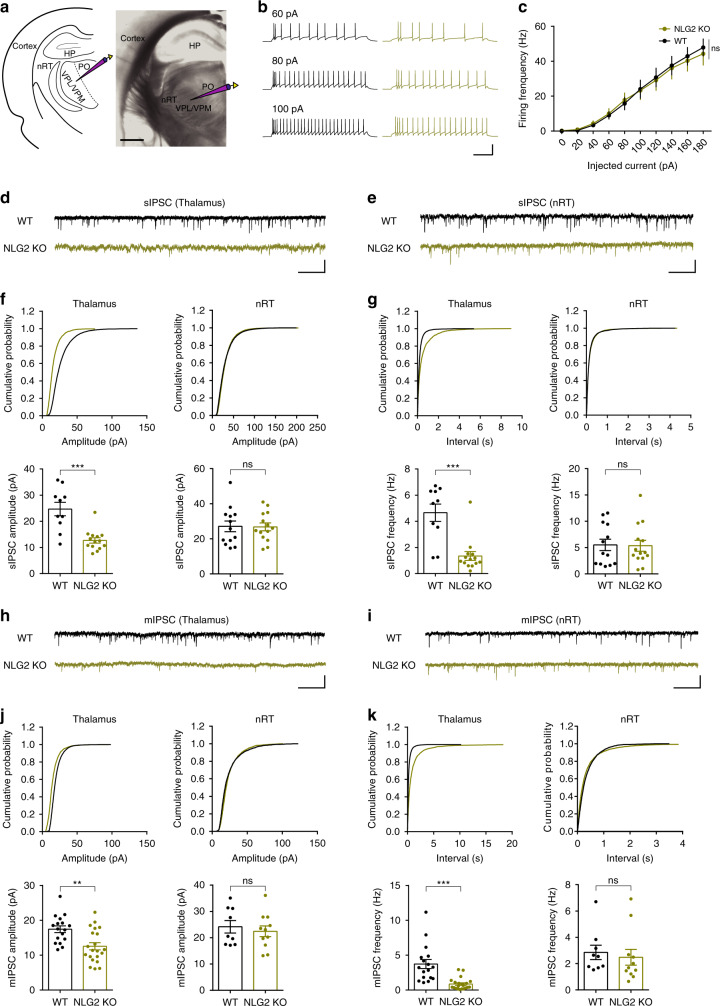
NLG2 KO thalamic neurons exhibit impaired GABAergic transmission. **a** Positioning of whole-cell recording electrode in the ventrobasal (VB) thalamus. HP hippocampus, VPL/VPM ventral posterolateral/ventral posteromedial nucleus, PO posterior nucleus. Image is a representative of five replicates. Scale bar: 1 mm. **b** Representative traces showing responses to 60, 80, and 100 pA current injections in WT (left) and NLG2 KO (right) thalamic neurons initially held at −60 mV. Scale bar: 0.2 s/60 mV. **c** Summary graph of spike frequency of thalamic neurons in response to various depolarizing currents showing no differences (two-way ANOVA, *p* = 0.787) in WT (*n* = 14) and NLG2 KO (*n* = 10) mice. **d**, **e** Sample sIPSC traces of thalamic (**d**) and nRT (**e**) neurons. Scale bars: 1 s/20 pA. **f** Cumulative histograms (top) and summary data (bottom) showing a significant reduction in sIPSC amplitude in thalamic neurons (left: *p* < 0.0001, NLG2 KO: *n* = 14, WT: *n* = 10), but not in nRT neurons (right: *p* = 0.9131, NLG2 KO: *n* = 14, WT: *n* = 13) in NLG2 KO compared to WT mice. **g** Cumulative histograms (top) and summary data (bottom) showing a significant reduction in sIPSC frequency in thalamic neurons (left: *p* < 0.0001, NLG2 KO: *n* = 14, WT: *n* = 10), but not in nRT neurons (right: *p* = 0.9726, NLG2 KO: *n* = 14, WT: *n* = 13), in NLG2 KO compared to WT mice. **h**, **i** Sample mIPSC traces of thalamic (**h**) and nRT (**i**) neurons. Scale bars: 1 s/20 pA. **j** Cumulative histograms (top) and summary data (bottom) showing a significant reduction in mIPSC amplitude in thalamic neurons (left: *p* = 0.0017, NLG2 KO: *n* = 21, WT: *n* = 17), but not in nRT neurons (right: *p* = 0.5879, NLG2 KO: *n* = 11, WT: *n* = 9), in NLG2 KO compared to WT mice. **k** Cumulative histograms (top) and summary graphs (bottom) showing a significant reduction in mIPSC frequency in thalamic neurons (left: *p* < 0.0001, NLG2 KO: *n* = 21, WT: *n* = 17), but not in nRT neurons (right: *p* = 0.8472, NLG2 KO: *n* = 11, WT: *n* = 9), in NLG2 KO compared to WT mice. Data in **c**, **f**, **g**, **j**, and **k** represent mean ± SEM. Two-sided *t*-test was used for **f**, **g**, **j**, and **k**. *p < 0.05, **p < 0.01, ***p < 0.001. ns nonsignificant. Source data are provided as a Source Data file.

Absence seizures are characterized by hypersynchronous cortical activity thought to be generated within the thalamocortical circuit^16–18,34^. Somatosensory cortex, ventrobasal thalamus, and nRT are particularly important in thalamorcortical circuit^35–38^. The cortex and thalamus are connected by reciprocal long-range excitatory projections whereas the interposed nRT is made of GABA-expressing neurons that project to the ventrobasal nuclei of the thalamus, but not to the cortex^36,39^. The disruption in the E/I balance within the thalamocortical circuitry is thought to underlie the SWDs, absence seizures and other forms of epileptic behaviors^16–18,36,40^. Therefore, it is possible that the SWDs in NLG2 KO mice are related to abnormalities of this circuitry. To test this hypothesis, we first performed EEG recordings from multiple brain regions simultaneously. The SWDs were observed in both the cortex and thalamus, but not in the hippocampus (Supplementary Fig. 2). To determine whether the SWDs originate in the cortex or thalamus, we locally injected ETX specifically to the cortex, thalamus or hippocampus. As shown in Fig. 2b–d, only local injections of ETX to the thalamus significantly decreased the proportion of SWDs, suggesting a critical role of the thalamus in the generation of SWDs in NLG2 KO mice. Thus, for the rest of the study we focused on the thalamus to investigate the underlying cellular and molecular mechanisms. Because NLG2 is a potent positive regulator of GABAergic synaptic transmission^14,27–31^, it is possible that the SWDs in NLG2 KO mice were caused by impairments in GABAergic transmission. To test this, we systemically injected diazepam, a positive allosteric modulator of GABA_A_ receptor, to the KO mice and found that diazepam abolished the SWDs to the same degree as ETX (Fig. 2e). In addition, local injections of diazepam to the thalamus, but not to the cortex or hippocampus, decreased the proportion of SWDs (Fig. 2f–h), suggesting that the abnormal SWDs in NLG2 KO mice may be due to impaired GABAergic transmission in the thalamus. To directly investigate this possibility, we performed whole-cell patch-clamp recordings from the ventrobasal thalamic (including ventral posterolateral and ventral posteromedial nucleus-VPL/VPM), nRT and somatosensory cortical layer 5/6 neurons (Fig. 3, Supplementary Figs. 3–5). We first compared various membrane properties of these neurons, including the intrinsic neuronal excitabilities, and found no differences between WT and NLG2 KO mice (Fig. 3a–c, Supplementary Fig. 4a–c, Supplementary Fig. 5a–c, Supplementary Table 1). We then compared synaptic transmission by recording spontaneous excitatory postsynaptic current (sEPSC), spontaneous inhibitory postsynaptic current (sIPSC), and miniature inhibitory postsynaptic current (mIPSC). We found no differences between genotypes in sEPSC, sIPSC, or mIPSC in either nRT (Fig. 3e–g, i–k; Supplementary Fig. 4d–f) or cortical (Supplementary Fig. 5d–f) neurons. In contrast, the thalamic neurons in NLG2 KO mice exhibited a significant reduction in both frequency and amplitude of sIPSC and mIPSC (Fig. 3d, f, g, h, j, k). No changes in sEPSC were observed in the thalamic neurons of the KO mice (Supplementary Fig. 3). Since the nRT projection to the thalamic neurons represents the main inhibitory input to the ventrobasal thalamus, our results suggest that deficits at the nRT-thalamic GABAergic synapse may underlie the reduced inhibitory synaptic transmission and SWDs in the thalamus. Because the thalamus is a large heterogenous brain area, we also recorded sEPSC and sIPSC from neurons in the posterior (PO) nucleus of the thalamus but found no significant differences between WT and NLG2 KO mice (Supplementary Fig. 6), suggesting that the ventrobasal thalamic VPL/VPM, but not the PO region, is the main target of the NLG2 effect.

### ETX alleviates behavior deficits in NLG2 KO mice

**Fig. 4:**
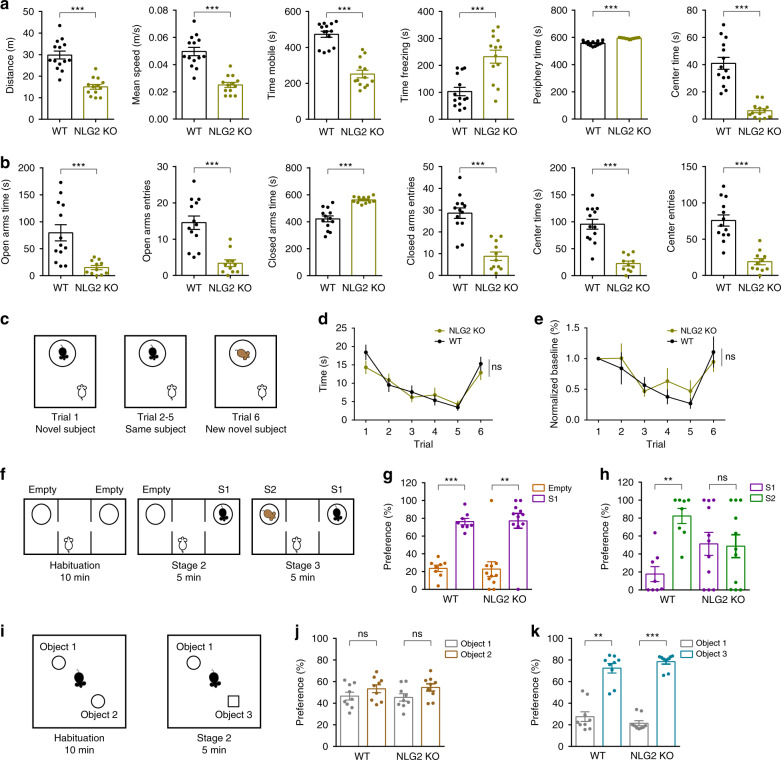
Reduced locomotion, increased anxiety, and impaired social memory in NLG2 KO mice. **a** Open field test showing decreased travel distance (*p* < 0.0001), travel speed (*p* < 0.0001), and mobile time (*p* < 0.0001), increased freezing time (*p* < 0.0001), increased time spent in periphery zone (*p* < 0.0001), and decreased time spent in center zone (*p* < 0.0001) in NLG2 KO (*n* = 13) compared with WT mice (*n* = 14). **b** Elevated plus maze test showing decreased time spent in open arms (*p* = 0.0009), decreased open arms entries (*p* < 0.0001), increased time spent in closed arms (*p* < 0.0001), decreased closed arm entries (*p* < 0.0001), decreased time spent in center (*p* < 0.0001), and decreased center entries (*p* < 0.0001) in NLG2 KO (*n* = 11) compared with WT mice (*n* = 13). **c** Schematic diagram of the five-trial habituation/dishabituation assay. **d** Summary data of the five-trial assay showing similar memory acquisition during trials 1–5 and preference for a novel mouse in trial 6 (two-way ANOVA, *p* = 0.4640) in WT (*n* = 18) and NLG2 KO mice (*n* = 18). **e** Normalized interaction time of (**d**) showing no differences (two-way ANOVA, *p* = 0.5322) between WT (*n* = 18) and NLG2 KO (*n* = 18) mice. **f** Schematic diagram of the three-chamber social interaction test. **g** Normalized interaction time showing similar preference for S1 over empty cage during stage 2 of the three-chamber social test in WT (*n* = 8) and NLG2 KO mice (*n* = 11) (WT: *p* = 0.0002, NLG2 KO: *p* = 0.0083). **h** Impaired preference for S2 over S1 during stage 3 of the three-chamber social interaction test in NLG2 KO (*n* = 11) compared to WT mice (*n* = 8) (WT: *p* = 0.0061, NLG2 KO: *p* = 0.9237). **i** Schematic diagram of the novel object recognition test. **j** Novel object recognition test showing similar preference for object 1 and object 2 in WT (*n* = 9) and NLG2 KO mice (*n* = 9) (WT: *p* = 0.3748, NLG2 KO: *p* = 0.2149). **k** Increased preference for object 3 over object 1 during stage 2 in both WT (*n* = 9) and NLG2 KO mice (*n* = 9) (WT: *p* = 0.001, NLG2 KO: *p* < 0.0001). Data in **a**, **b**, **d**, **e**, **g**, **h**, **j**, and **k** represent mean ± SEM. Two-sided *t*-test was used for data in **a**, **b**, **g**, **h**, **j**, and **k**. *p < 0.05, **p < 0.01, ***p < 0.001. ns nonsignificant. Source data are provided as a Source Data file.

**Fig. 5:**
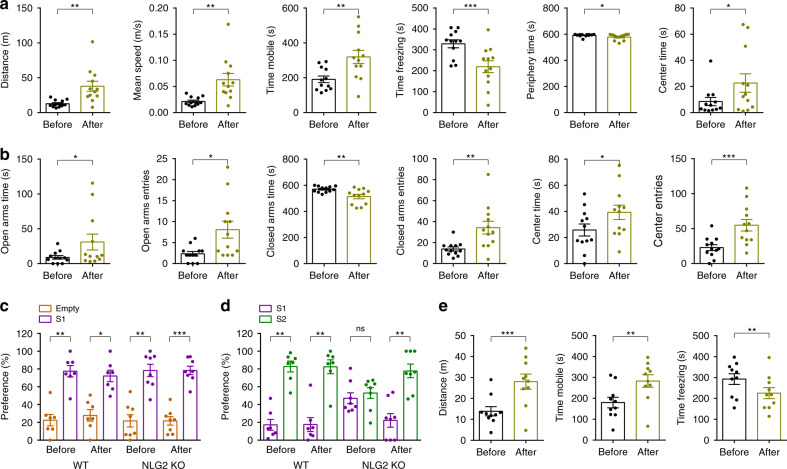
ETX ameliorates behavior deficits in NLG2 KO mice. **a** Open field test showing increased travel distance (*p* = 0.0022), increased travel speed (*p* = 0.0022), increased mobile time (*p* = 0.001), decreased freezing time (*p* = 0.0005), decreased time spent in periphery zone (*p* = 0.0165), and increased time spent in center zone (*p* = 0.0207) after systemic injections of ETX compared to before injections in NLG2 KO mice (*n* = 12). **b** Elevated plus maze test showing increased time spent in open arms (*p* = 0.0484), increased open arm entries (*p* = 0.0100), decreased time spent in closed arms (*p* = 0.0055), increased closed arm entries (*p* = 0.0036), increased time spent in center (*p* = 0.0172), and increased center entries (*p* = 0.0007) after systemic injections of ETX compared to before injections in NLG2 KO mice (*n* = 12). **c** Three-chamber social interaction test showing normal preference for S1 over empty cage during stage 2 after systemic injections of ETX in NLG2 KO mice (before treatment: *p* = 0.0045, after treatment: *p* = 0.0005, *n* = 8). **d** Rescue of preference for S2 over S1 during stage 3 of the three-chamber social interaction test by ETX treatment in NLG2 KO mice (before treatment: *p* = 0.6516; after treatment: *p* = 0.0084) in NLG2 KO mice (*n* = 8). **e** Open field test showing increased travel distance (*p* = 0.0005), increased mobile time (*p* = 0.0037), and decreased freezing time (*p* = 0.008) after local injections of ETX to the thalamus in NLG2 KO mice (n = 10). Data in **a**–**e** represent mean ± SEM. *p < 0.05, **p < 0.01, ***p < 0.001 with two-sided *t*-test. ns nonsignificant. Source data are provided as a Source Data file.

Previous studies showed that NLG2 KO mice spent less time in the center area in an open field, suggesting an anxiety-like behavior^41^, but the underlying mechanism is unknown. Clinical data indicate that anxiety is associated with epilepsy and up to 60% of patients with chronic epilepsy have various mood disorders including depression and anxiety^42–44^. Associations were also found between the anxiety disorders and ASD^45^. These results suggest that the SWDs in NLG2 KO mice might be responsible for the behavior changes in these KO mice. To test this possibility, we first confirmed the behavior deficits of NLG2 KO mice. In an open field test, the NLG2 KO mice showed a decrease in total travel distance, mean speed, mobile time, and an increase in freezing time compared to WT littermates (Fig. 4a). This reduced locomotor activity in the KO mice is consistent with their increased behavior arrests observed in the EEG/EMG recording (Fig. 1a, Supplementary Fig. 1). NLG2 KO mice also spent more time in the periphery zone and less time in the center zone compared to WT mice (Fig. 4a). In an elevated plus maze test, NLG2 KO mice spent less time and entries in the open arms and center zone, and more time in the closed arms (Fig. 4b). These results are consistent with previous studies^41^ and suggest an increased anxiety-like behavior in the KO mice. In a five-trial social habituation and dishabituation test, both WT and KO mice showed a gradual reduction in interaction time across five successive trials with the same stranger mouse and a significant increase in interaction time with a new stranger mouse in trial six (Fig. 4c–e), suggesting normal social habituation/dishabituation behavior in the KO mice. In the three-chamber social interaction test, both WT and NLG2 KO mice spent more time interacting with a stranger mouse (S1) over an empty cage (stage 2), suggesting normal sociability (Fig. 4f, g). However, during stage 3 of the test, only WT, but not NLG2 KO mice, showed a preference to a novel stranger mouse (S2), suggesting impaired social recognition memory in the KO mice (Fig. 4f, h). In the novel object recognition test, both NLG2 KO and WT showed a similar preference to a new object (Fig. 4i–k), suggesting that the KO mice can recognize novelty and form memory. Therefore, for the rest of the study we focused on the open field, elevated plus maze and three-chamber social interaction tests to further investigate whether the behavioral deficits in NLG2 KO mice were caused by SWDs and impaired GABAergic transmission at the nRT-thalamic circuit. To test the role of the SWDs, we repeated the behavior tests before and after systemic injections of ETX at 200 mg/kg, the same dose shown to completely eliminate the SWDs in the KO mice (Fig. 2a). In the open field test, ETX significantly increased total travel distance, mean speed, mobile time, and time spent in center zone, and significantly decreased freezing time and time spent in the periphery zone in NLG2 KO mice (Fig. 5a). In the elevated plus maze test, ETX also significantly increased open arm entries, time spent in open arms, center entries and time spent in center arms, and significantly decreased time spent in closed arms in the KO mice (Fig. 5b). In the three-chamber social interaction test, although ETX had no effect on sociability in stage 2, it significantly enhanced preference to S2 over S1 during the stage 3 memory test in NLG2 KO mice (Fig. 5c, d). To confirm that the thalamus is involved, we locally injected ETX to the thalamus and performed the open field test. As shown in Fig. 5e, local injection of ETX to the thalamus significantly increased total travel distance, mobile time and significantly decreased freezing time in NLG2 KO mice, consistent with a critical role of thalamic SWDs in these behavioral changes in the KO mice. To test the possibility that ETX might ameliorate the behavioral deficits of NLG2 KO mice through mechanisms independent of the SWDs, we first performed ETX injection experiments in WT mice but found that ETX had no effects on locomotion, anxiety and social recognition memory (Fig. 5c, d, Supplementary Fig. 7). Second, we tested the effect of ETX in PAK3 transgenic mice, which exhibit similar social recognition memory deficits but have no seizures^46^. As shown in Supplementary Fig. 8, ETX treatment did not rescue the social memory deficits in PAK3 transgenic mice. Finally, we tested the effect of ETX in the FMR1 KO mice, a widely used mouse model for syndromic ASD that show social recognition memory deficits^47^. The FMR1 KO mice also have increased susceptibility to seizures, which differ from absence seizures^48^. As shown in Supplementary Fig. 9, ETX also did not improve the social memory impairments in these mice. Taken together, these results suggest that ETX alleviates the behavioral deficits of NLG2 KO mice through blocking the SWDs. To investigate how ETX might block the SWDs in NLG2 KO mice, we tested the effect of ETX on the intrinsic neuronal properties and IPSCs of the thalamic neurons in brain slices. As shown in Supplementary Fig. 10, ETX (500 uM) did not affect sIPSCs or action potentials induced by depolarizing currents in either WT or KO mice, but it reduced the number of action potentials in the rebound burst firing in both genotypes. Thus, ETX disrupts the SWDs likely through reducing burst firing of thalamic and/or nRT neurons, consistent with its action on T-type Ca^2+^ channels^18^.

### Optical activation of the nRT-thalamic pathway

**Fig. 6:**
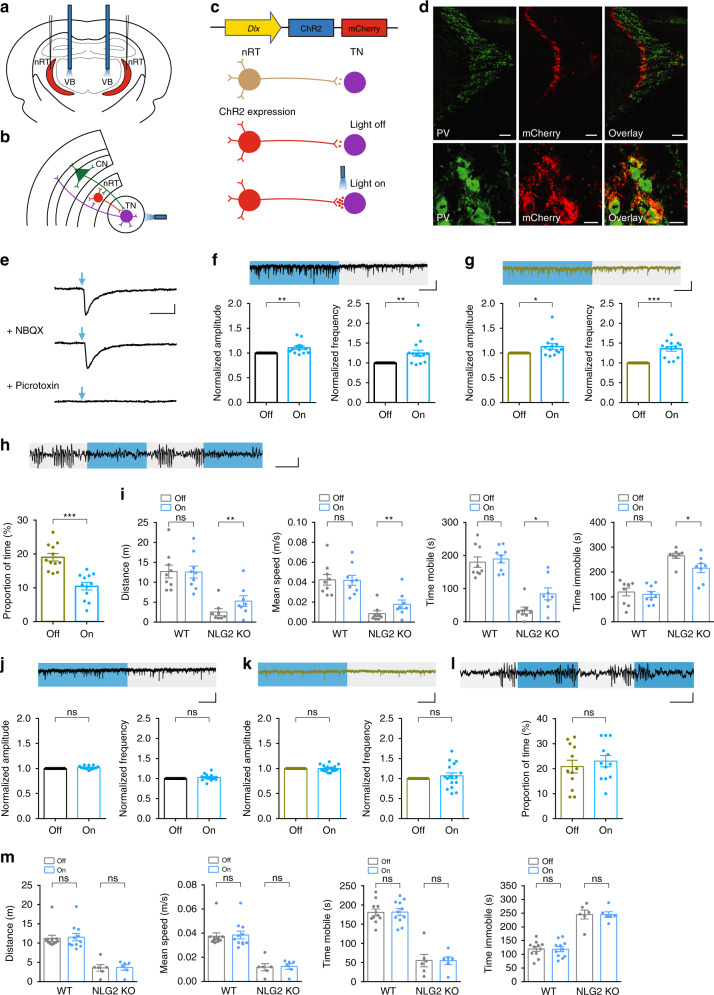
Optical activation of the nRT-thalamic circuit reduces synaptic and behavior deficits in NLG2 KO mice. **a** Optical fiber location in ventrobasal thalamus (VB) and virus injection site in nRT. **b** Schematic of the optical stimulation of the nRT-thalamic circuit. CN cortical neuron, TN thalamic neuron. **c** Viral ChR2-mCherry construct, expression and optical activation of the nRT-thalamic terminal. **d** Immunostaining images of ChR2-mCherry (red) and PV+ neurons (green) in the nRT region. Scale bars: 100 μm (top), 10 μm (bottom). Images are representatives of three replicates. **e** Whole-cell recording of thalamic neurons of ChR2-mCherry infected mice, showing IPSCs evoked by the 455 nm blue-light illumination (1 ms) and blocked by picrotoxin but not NBQX and APV. Scale bar: 20 ms/100 pA. **f** Increased sIPSC amplitude (*p* = 0.0098) and frequency (*p* = 0.0074) with blue-light on compared to blue-light off in WT mice injected with ChR2-mCherry viruses (*n* = 13). Scale bar: 1 s/20 pA. **g**, Increased sIPSC amplitude (*p* = 0.0418) and frequency (*p* < 0.0001) with blue-light on compared to blue-light off in NLG2 KO mice injected with ChR2-mCherry viruses (*n* = 13). Scale bar: 1 s/20 pA. **h** Decreased SWDs with blue-light on compared to blue-light off in NLG2 KO mice (*n* = 12) injected with ChR2-mCherry viruses (*p* < 0.0001). Scale bar: 2 s/100 μV. **i** Increased travel distance (*p* = 0.0040), speed (*p* = 0.0026), mobile time (*p* = 0.0197), and decreased immobile time (*p* = 0.0198) with alternate 5 s blue-light on and off compared to blue-light off in NLG2 KO mice injected with ChR2-mCherry viruses (*n* = 8). **j** Similar sIPSC amplitude (*p* = 0.0695) and frequency (*p* = 0.2639) with blue-light on compared to blue-light off in WT mice (*n* = 14) injected with GFP control viruses. Scale bar: 1 s/20 pA. **k** Similar sIPSC amplitude (*p* = 0.9756) and frequency (*p* = 0.3797) with blue-light on compared to blue-light off in NLG2 KO mice (*n* = 17) injected with GFP control viruses. Scale bar: 1 s/20 pA. **l** Similar SWDs with blue-light on compared to blue-light off (*p* = 0.1978)) in NLG2 KO mice (*n* = 12) injected with GFP control viruses. Scale bar: 2 s/100 μV. **m** Similar travel distance (WT: *p* = 0.812, NLG2 KO: *p* = 0.8903), speed (WT: *p* = 0.8254, NLG2 KO: *p* = 0.86), mobile time (WT: *p* = 0.9461, NLG2 KO: *p* = 0.9796), and immobile time (WT: *p* = 0.9454, NLG2 KO: *p* = 0.9807) with alternate 5 s blue-light on and off compared to blue-light off in WT (*n* = 11) and NLG2 KO mice (*n* = 6) injected with GFP control viruses. Data in **f**–**m** represent mean ± SEM. *p < 0.05, **p < 0.01, ***p < 0.001 with two-sided paired *t*-test. ns nonsignificant. Source data are provided as a Source Data file.

Our data that IPSCs recorded from the thalamic neurons were impaired (Fig. 3) and that the local injection of diazepam to the thalamus blocked SWDs (Fig. 2h) suggest that the impaired GABAergic transmission at the nRT-thalamic circuit may be responsible for the SWDs and behavioral deficits in NLG2 KO mice. To directly test this possibility, we used an optogenetic approach to specifically activate the nRT-thalamic synapse. We injected adeno-associated viruses (AAV) expressing channelrhodopsin-mCherry (ChR2-mCherry) or control GFP to the nRT. The ChR2-mCherry or GFP was under the control of the mouse *Dlx* promotor to restrict its expression to the inhibitory neurons within the nRT^49^. Optic fibers were implanted bilaterally above the ventrobasal thalamus to allow for the optical stimulation of the nRT terminals onto the thalamic neurons via a continuous delivery of 473 nm blue light (Fig. 6a–c). Immunostaining experiments showed that mCherry was colocalized with a subset of parvalbumin (PV)-positive neurons in the nRT (Fig. 6d). First, we identified the thalamic neurons that were innervated by the ChR2-expressing presynaptic terminals by the presence of IPSCs evoked by blue-light optical stimulation and confirmed that these IPSCs were blocked by picrotoxin (100 μM), but not by NBQX (10 μM) and APV (50 μM) (Fig. 6e) in acute brain slices. We then recorded sIPSCs from these identified neurons with or without 5 s epoch of continuous blue-light stimulation. As shown in Fig. 6f, g, the blue-light illumination increased both the frequency and amplitude of sIPSC in WT and NLG2 KO mice compared to no light illumination. Therefore, optical stimulation of the nRT terminals enhanced GABAergic transmission in the thalamic neurons. To test whether this enhanced synaptic transmission affected the SWDs in NLG2 KO mice, we recorded EEG signals in NLG2 KO mice expressing ChR2-mCherry and found the same optical stimulation (5 s epoch of continuous blue-light illumination) reduced the proportion of SWDs compared to no light illumination (Fig. 6h). To extend this effect on behavior, we compared the locomotor activity with or without the optical stimulation in the open field test. As shown in Fig. 6i, the blue-light illumination increased the total travel distance, mean speed and mobile time, and decreased the arrest time in NLG2 KO, but not in WT mice. In contrast, 5 s epoch of continuous blue-light stimulation had no effects on thalamic slice sIPSCs (Fig. 6j, k), the SWDs (Fig. 6l) or locomotor activity (Fig. 6m) in animals infected with control GFP virus in either WT or NLG2 KO mice. Similarly, the blue-light stimulation had no effects on IPSCs and locomotor activities in animals that only had optical fiber implantation, without any virus injection (Supplementary Fig. 11). In summary, these results suggest that optogenetic activation of the nRT-thalamic pathway is able to partially rescue GABAergic transmission, SWDs, and behavior arrests in NLG2 KO mice.

### Viral expression of NLG2 in thalamic neurons in NLG2 KO mice

**Fig. 7:**
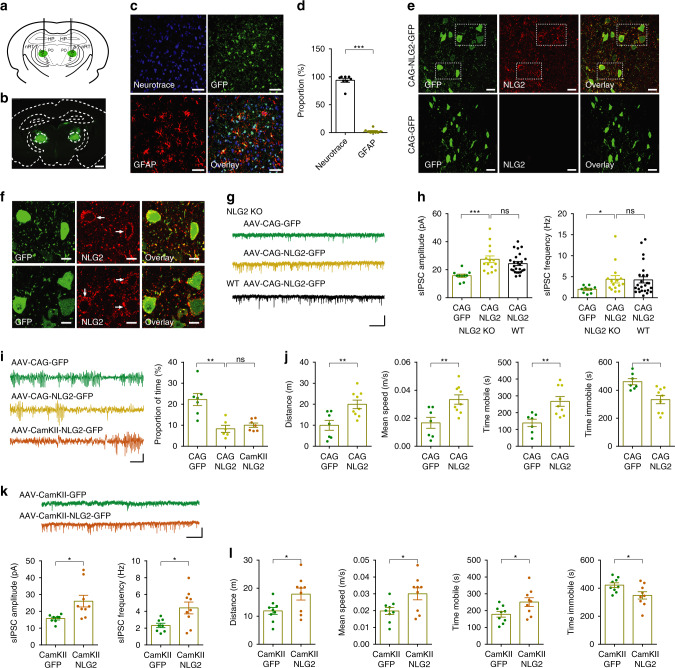
Reintroducing NLG2 to ventrobasal thalamus reduces synaptic and behavior deficits in NLG2 KO mice. **a** Injection sites of AAV-NLG2-GFP viruses in the ventrobasal thalamus. **b** Expression of AAV-NLG2-GFP. Scale bar: 1 mm. Images are representatives of five replicates. **c** Expression of NLG2-GFP (green), Neurotrace (blue) and GFAP (red) in cortical sections infected with AAV-NLG2-GFP viruses. Scale bars: 50 μm. Images are representatives of three replicates. **d** Summary graph of cortical staining showing NLG2-GFP colocalized with Neurotrace but not GFAP (*p* < 0.0001, *n* = 8). **e** Expression of NLG2 (red) in NLG2 KO mice injected with CAG-NLG2-GFP, but not in NLG2 KO mice injected with CAG-GFP control viruses. Scale bars: 20 μm. Images are representatives of six replicates. **f** High-magnification images showing expression of NLG2 (red, arrows) on thalamic neuronal surface (green) in NLG2 KO mice injected with CAG-NLG2-GFP viruses. Scale bars: 10 μm. Images are representatives of six replicates. **g** Sample traces of sIPSCs in NLG2 KO and WT mice injected with CAG-NLG2-GFP or CAG-GFP viruses. Scale bar: 1 s/20 pA. **h** Increased sIPSC amplitude (*p* = 0.0009) and frequency (*p* = 0.0437) in NLG2 KO thalamic neurons expressing CAG-NLG2-GFP (*n* = 15) compared to CAG-GFP (*n* = 10). Similar sIPSC amplitude (*p* = 0.2363) and frequency (*p* = 0.8983) between WT (*n* = 25) and NLG2 KO (*n* = 15) thalamic neurons expressing CAG-NLG2-GFP. **i** Decreased SWDs (*p* = 0.0019) in NLG2 KO mice virally expressing CAG-NLG2-GFP (*n* = 6) compared to CAG-GFP control (*n* = 7), and a similar reduction (*p* = 0.3858) in SWDs with viral expression of CAG-NLG2-GFP (*n* = 6) compared with CamKII-NLG2-GFP (*n* = 7). Scale bar: 2 s/100 μV. **j** Increased travel distance (*p* = 0.006), speed (*p* = 0.0058), mobile time (*p* = 0.0048), and decreased immobile time (*p* = 0.0048) in NLG2 KO mice injected with CAG-NLG2-GFP (*n* = 9) compared to control CAG-GFP viruses (*n* = 7). **k** Increased amplitude (*p* = 0.0153) and frequency (*p* = 0.0185) in NLG2 KO thalamic neurons virally expressing CamKII-NLG2-GFP (*n* = 9) compared to CamKII-GFP control (*n* = 8). Scale bar: 1 s/20 pA. **l** Increased travel distance (*p* = 0.0265), speed (*p* = 0.0235), mobile time (*p* = 0.0315), and decreased immobile time (*p* = 0.0316) in NLG2 KO mice injected with CamKII-NLG2-GFP (*n* = 9) compared to control CamKII-GFP viruses (*n* = 9). Data in **d**, **h**–**l** represent mean ± SEM. *p < 0.05, **p < 0.01, ***p < 0.001 with two-sided paired *t*-test. ns nonsignificant. Source data are provided as a Source Data file.

To determine whether the lack of NLG2 in the postsynaptic neurons of the nRT-thalamic pathway is responsible for the impaired GABA transmission and absence seizures in NLG2 KO mice, we reintroduced the WT NLG2 back to the KO mice by injecting AAV expressing NLG2 fused to GFP, under the control of a general CAG promoter, specifically to the ventrobasal thalamus (Fig. 7a). Immunostaining experiments 4 weeks after the injection showed that the expression of NLG2-GFP was restricted to the ventrobasal thalamus (Fig. 7b) and largely colocalized with the neuronal marker Neurotrace in NLG2 KO mice (Fig. 7c, d). The NLG2-GFP, but not control GFP, infected KO mice showed an increased level of NLG2 (Fig. 7e) whose expression was mainly seen on the neuronal surface (Fig. 7f). To determine whether the expression of NLG2-GFP affected GABAergic transmission, we recorded sIPSC from the thalamic neurons in brain slices prepared from either WT or NLG2 KO mice 4 weeks after the viral injection. As shown in Fig. 7g, h, NLG2 KO and WT neurons infected with NLG2-GFP showed similar levels of sIPSC frequency and amplitude, which were significantly higher than those from NLG2 KO neurons infected by control GFP, indicating that the impaired GABAergic transmission was rescued in NLG2 KO mice. To determine the effect of NLG2-GFP expression on the SWDs, we performed EEG recordings in NLG2 KO mice 4 weeks after the viral injection. As shown in Fig. 7i, the SWDs were significantly decreased in NLG2-GFP infected NLG2 KO mice compared to GFP infected KO mice. To determine the effect of NLG2-GFP expression on the behavior arrests of the KO mice, we performed the open field test and showed that the total travel distance, mean speed, and mobile time were increased, whereas the immobile time was decreased in NLG2 KO mice injected with NLG2-GFP compared to control GFP alone (Fig. 7j). We also repeated the above experiments with separate AAV viruses where the expression of NLG2-GFP or GFP was under the control of the excitatory neuronal marker CamKII promoter and found that the expression of NLG2-GFP, but not GFP control, in the thalamic neurons of NLG2 KO mice rescued GABAergic transmission (Supplementary Fig. 12, Fig. 7k), reduced the SWDs (Fig. 7i), increased travel distance, mean speed, and mobile time and decreased the immobile time (Fig. 7l) in the KO mice. Therefore, the reintroduction of WT NLG2 to the ventrobasal thalamic neurons is sufficient to restore GABA transmission, and partially rescue absence seizures and behavior arrests in NLG2 KO mice. Consistent with no changes in inhibitory synaptic transmission in the PO region of the thalamus (Supplementary Fig. 6), the expression of NLG2-GFP in this region did not rescue the SWDs or motor deficits (Supplementary Fig. 13).

## Discussion

In this study, we revealed a role for NLG2 in electrographic seizures, behavior arrests, and underlying synaptic mechanisms. We show that NLG2 KO mice exhibit abnormal 5–8 Hz SWDs and concurrent motor freezing. These SWDs and behavior arrests are blocked by ETX and diazepam, administered either systematically or locally to the thalamus, and therefore likely represent a form of absence seizures. In addition, we show that inhibitory synaptic transmission in NLG2 KO mice is impaired in the ventrobasal thalamic neurons, but not in the cortical or nRT neurons, and that enhancing synaptic transmission, either by optical activation of the nRT-thalamic terminals or re-expression of NLG2 in the thalamic neurons, decreases the SWDs and motor freezing in the KO mice. These results provide strong evidence that NLG2-mediated GABAergic transmission is a powerful regulator of thalamocortical network activities related to absence seizures and may provide a molecular mechanism underlying high rates of comorbidity of ASDs with increased epileptic seizures and anxiety.

An important finding of the present study is that the absence seizure-like SWDs are specific to NLG2 KO mice. No epileptic EEG signals were observed in NLG1 KO or NLG3 KO mice. The reason for this unique phenotype in NLG2 KO mice is unknown but may reflect its specific role in inhibitory synaptic inhibition and E/I balance. It is known that NLG2 is exclusively localized at inhibitory synapses and exerts potent effects on the development and function of these synapses^14,27–31,50,51^. The seizure phenotype in the NLG2 KO mice is consistent with several previous studies showing the involvement of related molecules in both seizures and ASD. It was reported that patients with mutations in neurexin gene, a presynaptic partner of NLGs, exhibit 65% recurrent ASD phenotype and 43% recurrent seizures^52^. In addition, mutations in contactin associated protein-like 2 gene (CNTNAP2), a member of the neurexin superfamily, are linked to childhood-onset epilepsy along with intellectual disabilities and autism^50,53^. Mouse models of CNTNAP2 deficiency show hyperactivity and seizures^54^. Another related protein CNTNAP4 is also associated with ASD and enriched in cortical interneurons^50,55,56^ and CNTNAP4 KO mice show autistic-like repetitive behaviors and epileptiform-like activity^50,57^. However, despite these studies, there remains a paucity of direct evidence to support the role of NLGs in linking epileptic seizures and ASD. Our data that NLG2 KO mice exhibit both absence seizure-like SWDs and autism-related behavior provide strong evidence in this regard. Thus, NLG2 KO mice may provide a useful mouse model for studying seizures and ASD and underlying mechanisms.

The present study also reveals the circuits and synaptic mechanisms by which NLG2 regulates SWDs and behavior. The thalamocortical network has been studied extensively and is widely regarded as a key player that generates rhythmic neuronal activities involved in both physiological processes as well as neurodevelopmental and neurological disorders, including epilepsy and ASD^16–18^. The thalamic neurons can generate autonomous, 1–2 Hz rhythmic oscillations, and through reciprocal synaptic communication with the nRT neurons, 7–14 Hz sleep spindle oscillations, that are thought to play a role in promoting slow-wave sleep^18^. The thalamus is also involved in cognitive processes including memory^58–61^. Recent studies also implicated the thalamic circuits in ASD. Human studies show changes in thalamocortical connectivity in ASD patients^62–64^ and genetic deletions of ASD-linked genes result deficits in the nRT and thalamic neurons and ASD-like phenotypes^61,65^. Our results are consistent with the view that the thalamus is engaged in both seizures and ASD. Although NLG2 KO mice exhibit SWDs in both the cortex and thalamus that are temporally correlated, the SWDs appear to originate from or at least critically depend on the thalamus because local injections of ETX or diazepam to the thalamus, but not to the cortex, diminished the SWDs. The reason for a partial blockade of the SWDs by these local injections is not known but may be due to the extent of drug diffusion. A previous study also reported that local application of ETX to the ventrobasal thalamus reduced, but not completely blocked SWDs in a rat model of absence epilepsy^66^. It is also possible that other brain regions such as cortical neurons may contribute to the SWDs in NLG2 KO mice as these neurons can generate SWDs independently of the thalamus^67,68^.

The nRT-thalamus projection provides powerful inhibition to the thalamic neurons in the ventrobasal thalamus^18^. Several previous studies have shown that perturbations of presynaptic function of this projection by manipulating the excitability or synaptic strength of the nRT neurons affect the neuronal rhythmic activities of the thalamic neurons and promote the generation of absence seizures^36,40^. However, few studies address the role of the postsynaptic properties and underlying molecular regulation of this pathway. We show that both sIPSC and mIPSC are dramatically reduced in the thalamic neurons in NLG2 KO mice. Importantly, excitatory synaptic transmission is not altered in the KO mice. In addition, synaptic strength or neuronal excitability of either somatosensory cortical or nRT neurons is not affected. These results suggest that the deletion of NLG2 causes SWDs and absence seizures likely by affecting inhibitory synaptic transmission in the thalamic neurons, but not in the cortical or nRT neurons. This conclusion is also supported by the observation that reintroducing NLG2 back to the thalamic neurons is sufficient to restore inhibitory transmission and decrease SWDs and behavioral arrests. These results together with the observation that optical activation of the nRT terminals onto the thalamic neurons can disrupt the SWDs and behavioral arrests suggest that postsynaptic NLG2 represents a key factor to regulate synaptic strength at the nRT-thalamic pathway and that its perturbation is sufficient to cause SWDs and recurrent absence seizures.

It is interesting to note that a previous study by Gilson and colleagues showed that inhibitory transmission (including mIPSCs) in the somatosensory cortical region is impaired in NLG2 KO mice^30^, whereas we found no significant impairments. This apparent discrepancy could be explained by the differences in the age of the mice used (2–3 weeks in the Gibson et al. study versus 6–18 weeks in the present study) and/or recorded neurons (layer 4 in the Gibson et al. study versus layer 5/6 neurons in the present study). In the Gibson et al. study it was also found that the IPSCs from the PV+ projection, but not from somatostatin positive projection, were reduced^30^. These results suggest that the effect of the NLG2 deletion is complex and may be regulated by both neuronal types and developmental stages.

Taken together, our results are consistent with a loss of feed-forward inhibition model for SWDs generation in NLG2 KO mice (Supplementary Fig. 14). In this model, a reduction in fast forward inhibition in the thalamic neurons causes hyperexcitability of thalamic-cortical circuit due to reciprocal excitatory input between thalamic and cortical neurons, resulting in the initiation of SWDs. Hyperactive thalamic neurons subsequently overactivate nRT neurons, causing them to fire bursts of action potentials and inhibit thalamic neurons, which in turn fire bursts of action potentials upon recovery from inhibition. This sequence of events leads to oscillation in the cortico-thalamo-cortical loop to maintain SWDs. The increased inhibition to thalamic neurons is possible because although fast inhibition mediated by synaptic GABA_A_ receptors is reduced in the thalamic neurons, there is no evidence that the tonic inhibition mediated by extrasynaptic GABA_A_ or GABA_B_ receptors is impaired in NLG2 KO mice.

Interestingly, transgenic mice overexpressing NLG2 in the entire brain also showed abnormal bilateral SWDs of 6–8Hz^33^, apparently similar to those observed in NLG2 KO mice. Therefore, one unresolved question is how deletion and overexpression of the same gene result in similar phenotype. Although the nature of SWDs in NLG2 transgenic mice remains to be further analyzed to confirm they are the same as those in the KO mice, it is possible that deletion and overexpression of NLG2 affect different populations of neurons. For example, overexpression of NLG2 may selectively increase GABAergic transmission in the nRT neurons, resulting in impaired synaptic inhibition to the thalamic neurons and increased excitability of these neurons, as occurred in the KO mice. It would important to determine synaptic strength in the nRT, thalamic, and cortical neurons in these transgenic mice.

An unsolved question is how NLG2 alters the inhibitory transmission in nRT-thalamic pathway? Previous studies have shown that NLG2 is localized at the inhibitory post synapse and regulates the recruitment of GABA_A_ receptors to the cell surface and GABAergic synaptic strength through NLG2-Gephyrin-Collybistin complex^14,27^. NLG2 is expressed in the postsynaptic membrane of the thalamic neurons, therefore it is possible that the deletion of NLG2 may cause reduction of synaptic GABA_A_ receptors, thus reduced synaptic response. This is consistent with the data that the GABA_A_ receptor allosteric modulator diazepam blocks SWDs in the KO mice. Another remaining question is how the deletion of NLG2 specifically impairs GABAergic transmission in the thalamic neurons, but not nRT and cortical neurons. It is possible that the levels of NLG2 expression in these neurons are different so that its loss may have differential effects. Another interesting question involves the tonic inhibitory transmission in the thalamic neurons. It is possible that although synaptic GABA_A_ receptors are reduced, extrasynaptic GABA_A_ or GABA_B_ receptors may not be altered or even enhanced, which promotes burst firing of the thalamic and nRT neurons and therefore maintaining the SWDs.

Previous studies showed that NLG2 KO mice exhibit decreased motor activity and coordination, increased anxiety, decreased social interaction and pain sensitivity^28,33,41^. We have confirmed these behavioral changes, and importantly revealed that the reduced locomotor activity, anxiety-like behavior, and impaired social memory can be ameliorated by global injection of the anti-absence seizure drug ETX. The fact that ETX has no effects in WT and two other ASD mouse models, that do not have the SWDs or absence seizures, suggests that the effects of ETX in NLG2 KO mice is likely mediated through blocking the SWDs. The data that optogenetic activation of the nRT-thalamic pathway or overexpression of WT NLG2 in the thalamic neurons reduces the SWDs and motor deficits in NLG2 KO mice also support this idea. However, it is important to note that ETX treatment (particularly the local injections in the thalamus) as well as optogenetic activation or re-expression of WT NLG2 only partially rescue motor deficits in NLG2 KO mice. Although the partial rescue in these experiments could be due to the extent of drug diffusion as mentioned earlier or the fact that only a fraction of nRT or thalamic neurons are infected, resulting in incomplete blockade of the SWDs, we cannot rule out the possibility that ETX may also act through other mechanisms independent of the SWDs and/or other brain regions also contribute to these behavioral deficits in NLG2 KO mice. Nevertheless, our study provides a molecular link between ASD phenotype and absence seizures through a NLG2-mediated inhibition at the nRT-thalamic synapse.

## Methods

### Animals

All mice used in this study were housed on a 12 h light 12 h dark cycle (8am–8pm) with 40–60% humidity, 22 °C ambient temperature and food and water ad libitum. NLG1 KO (#008136), NLG2 KO (#008139), and NLG3 KO (#008394) mice were obtained from the Jackson Laboratory. These mice were initially generated in 129 Sv × C57BL/6 mixed genetic background and upon arrival in the lab, they were backcrossed to C57BL/6 for a few generations. Therefore, the experimental mice used in this study were still considered to be on a 129 Sv × C57BL/6 mixed background. The following primers (ACGT, Toronto, ON, CA) were used for genotyping NLG KO mice: NLG1 KO: 5′-GTGAGCTGAATCTTATGGTTAGATGGG-3′ (common), 5′-CGAGAGTCAGGTAAATTGAACACCAC-3′ (WT reverse) and 5′-GAGCGCGCGCGGCGGAGTTGTTGAC-3′ (KO reverse); NLG2 KO: 5′-GTCTCAGTAAGCTTATTTGAGAAGCCAA-3′ (common), 5′-CTCTGGGCCTTCTCAGGACTGTAC-3′ (WT reverse) and 5′-GAGCGCGCGCGGCGGAGTTGTTGAC-3′ (KO reverse); NLG3 KO: 5′-ATGGGTGAGTTGTCCTTAGGC-3′ (common), 5′-GGGAGTGACTTGCTAGACAAG-3′ (WT forward) and 5′-CCATGTCACTACATGCTCTG-3′ (KO forward). FMR1 KO (#004624) and its control WT mice with the FVB background (#004828) were also obtained from the Jackson Laboratory. The tTA/PAK3 transgenic mouse model in a C57BL/6 J genetic background was described previously^46^. The following primers (ACGT, Toronto, ON, CA) were used for genotyping PAK3 transgenic mice: tTA-1 sense: 5′-CGCTGTGGGGCATTTTACTTTAG-3′, tTA-2 antisense: 5′-CATGTCCAGATCGAAATCGTC, mPAK3-GFP sense: 5′-GAACAGTAACAACCGAGACTC-3′ and mPAK3-GFP antisense: 5′-GGTGACTGCATCAAAACCCAC-3′. All the experimental mice were the littermates derived from heterozygous breeders, except that FMR1 KO and its control WT mice were obtained from homozygous breeders obtained from the Jackson Laboratory. Adult KO or transgenic mice and their aged (6–18 weeks) and sex matched WT littermates were used. All the experimental protocols used in this study were approved by the Lab Animal Use Committee of the Hospital for Sick Children, Toronto, Canada. The experimenters were blind to the genotype or treatment of the mice.

### Surgical procedures

For EEG electrode implantation, adult mice (12–18 weeks old) were anesthetized by IP administration of ketamine (100 mg/kg) and xylazine (10 mg/kg) prior to surgery. Mice were placed in a stereotaxic frame and three monopolar stainless-steel electrodes (wire diameter 0.010 in, length 1.15–1.25 mm, Roanoke, WA, USA) for EEG recordings and two insulated stainless-steel wires (wire diameter 0.011 mm, Cooner Wire, Chatsworth, CA, USA) for EMG recordings were prepared for stereotaxic surgery. Two electrodes were inserted through holes made in the skull over left frontal lobe (1.7 mm lateral to midline, 1.5 mm anterior to bregma) and right parietal lobe (1.7 mm lateral to midline, 1.0 anterior to lambda), and an additional electrode was inserted over cerebellum as reference. The two EMG electrodes were implanted into the neck muscle. For EEG recordings with cannula infusion and optical fibre insertion (see below), the stainless-steel wires were implanted through the three holes drilled in the left and right frontal cortex with coordinates ±1.7 mm medio-lateral (ML), +1.5 mm anterior-posterior (AP) and cerebellum with same coordinates. For multiple depth electrode EEG recordings, seven electrodes with a length of 1.15, 2.1, 3.35, and 1.5 mm for frontal cortex, hippocampus, thalamus and cerebellum, respectively, were stereotaxically implanted into the left and right frontal cortex with coordinates ±3 mm ML, +1.35 mm AP; left and right thalamus with coordinates ±1.35 mm ML, −0.6 AP; left and right hippocampus with coordinates ±1.5 mm ML, −2.35 mm AP; and one cerebellum as reference with coordinates 0 mm ML, −6.25 mm AP. Dental acrylic (Lang Dental, Wheeling, IL, USA) was used to stabilize the electrodes and wires. Following surgery, the animals were monitored until complete recovery from anesthesia. Anafen (5 mg/kg) was administered for postoperative pain control and 1 ml 0.9% saline was given for fluid loading. All animals had a minimum 7 days postoperative recovery period before experiments commenced.

For drug infusion cannula implantation, the skull of the mice was surgically exposed and two holes were drilled bilaterally to target the somatosensory frontal cortex (ML: ±3 mm, AP: +1.35 mm), hippocampus (ML: ±1.5 mm, AP: −2.35 mm), or ventrobasal thalamus (ML: ±1.5 mm, AP: −1.7 mm). In each hole drilled, a single guide cannula (diameter of 0.7 mm, made with 22 G TW needle, Becton Dickinson, Franklin Lakes, NJ, USA) with a length of 1.15, 2.1, and 3.5 mm for somatosensory cortex, hippocampus, and ventrobasal thalamus, respectively, was inserted slowly to corresponding coordinates, and capped with a cannula stopper (diameter of 0.3 mm, made with 30 G TW needle, Becton Dickinson, Franklin Lakes, NJ, USA). Dental acrylic was used to secure the cannulas. Surgically operated animals were recovered for a minimum 7 days before experiments. For local administration of diazepam (5 mg/ml, Sandoz, QC, CA) and ethosuximide (100 mg/ml, dissolved in 0.3% tween 80, Sigma–Aldrich, ON, CA), drugs were infused through a 30 G TW needle (Becton Dickinson, Franklin Lakes, NJ, USA) with a volume of 1 μl at a rate of 1 μl/minute using a Dual Syringe Infusion Pump (KD Scientific, Holliston, Massachusetts, USA). Cannula infusion of ETX or diazepam was performed 30 min prior to behavioral testing.

For viral injections and optical fibre placement, the AAV2/5-mDlx-GFP-Fishell-1 (titer 2 × 10^13^), AAV2/5-mDlx-ChR2-mCherry-Fishell-3 (titer 2 × 10^13^), AAV2/DJ-CAG-GFP (titer 5 × 10^13^), AAV2/DJ-CAG-Neuroligin 2-T2A-GFP (titer 2 × 10^13^), AAV2/DJ-CamKII-GFP (titer 1.3 × 10^13^), and AAV2/DJ-CamKII-Neuroligin 2-T2A-GFP (titer 1.3 × 10^13^) viruses were generated and acquired from Canadian Neurophotonics Platform (Quebec, QC, CA). All viruses were aliquoted and stored at −80 °C before use. Mouse skull was surgically exposed and the AAV2/DJ-CAG-GFP, AAV2/DJ-CAG-Neuroligin 2-T2A-eGFP, AAV2/DJ-CamKII-GFP, and AAV2/DJ-CamKII-Neuroligin 2-T2A-GFP viruses were injected into the ventrobasal thalamus (ML ±1.5 mm, AP −1.7 mm, DV −3.5 mm) and PO thalamus (ML ±1.2 mm, AP −1.9 mm, DV −3.25 mm) using a back-filled glass micropipette connected by tubing to a 10 μl Hamilton syringe. Injected volume (0.6 μl) and flow rate (100 nl/min) were controlled by a precision pump (World Precision Instruments, Sarasota, FL, USA). The coordinates for injecting 0.4 μl AAV2/5-mDlx-GFP-Fishell-1 and AAV2/5-mDlx-ChR2-mCherry-Fishell-3 to the nRT were 0.9 mm posterior to bregma, 1.9 mm lateral to midline, and 3 mm below the cortical surface. For optical fibre placement, custom made optical fibres (200 um diameter, 0.39NA) were secured to ceramic ferrules (230 um bore size, 1.25 um outer diameter) and bilaterally implanted above the ventrobasal thalamus (ML: ±1.8 mm, AP: −1.5 mm, DV: −3.5 mm). Optical fibres were fixed to skull with dental acrylic. All virus injected mice were recovered for at least 3–4 weeks to allow for NLG2 and ChR2 expression before experiments commenced. The virally encoded gene expression and the placement of optical fibres were confirmed by immunohistochemical staining.

### EEG recordings

Prior to recordings the mice were placed in a plexiglass cage, attached to EEG head wires and connected to Grass Instruments (Warwick, RI, USA) to allow the habituate in the recording cage. All mice were freely moving during the recording. The EEG and EMG signals were recorded, amplified and filtered from the bandwidth of 1 Hz low-frequency filter and 70 Hz high-frequency filter. For the 48 h continuous EEG-EMG recordings, baseline recording was produced after 3 days of habituation in the recording chamber. For the rest of EEG recordings, baseline recording was produced after 1 h habituation. The EEG and EMG signals were visually analyzed with 60 Hz notch filter. The gain setting of the EEG/EMG display signals were increased to 2-fold if necessary. The numbers and duration of SWDs were quantified with the following criteria: at least 1 s duration (the time interval between the onset and offset of the abnormal EEG activity), at least twice the amplitude of the background EEG signal. Two abnormal events separated by less than 0.5 s were counted as one SWD. For correlation analysis of the EEG and EMG amplitude, the data were obtained in consecutive 1 s epoch from randomly selected 160 s continuous recording signals. Average amplitude of each 1 s synchronized EEG and EMG traces were measured using GrassLab (Grass Instruments, Warwick, RI, USA). For EEG frequency power analysis, MatLab (MathWorks Natick, MA, USA) was used to subtract EEG signals for Fast Fourier Transformation (FFT). Only frequencies below 56 Hz were subjected to FFT following the background noise filtering. The filter setting used for high frequency is 0.55 Hz, band stop are 0.45 Hz and 60 Hz. Total EEG power (μV2) density was calculated between 1 and 56 Hz for each 4 seconds epoch, and the power density for each frequency bin was presented as a percentage of the average total EEG powers from 1 to 56 Hz. Pearson’s correlation analysis was used to analyze the correlation between the amplitude of synchronized EEG and EMG signals. For the SWDs and behavior arrest analysis, the EEG traces and mouse behavior in the EEG recording chamber were video recorded for at least 30 min after 1 h habituation. The movement of the mice was analysed in consecutive 1 s epoch from each SWDs within a continuous 30 min EEG recording. Freezing behavior refers to lack of any whole-body movement. Drug treatments by IP injection (1 mg/kg diazepam dissolved in saline, 200 mg/kg ETX dissolved in 0.3% tween 80) or cannula infusion were applied after 90 min baseline and recorded for additional 120 min after drug application. The control mice were treated with saline or 0.3% tween 80 in the same manner. For mice with ChR2 expression in the nRT neurons, synaptic activation was achieved by 473 nm blue light generated by a diode-pumped solid-state laser (Laserglow, Toronto, ON, CA) and delivered through the optical fibres bilaterally implanted above the ventrobasal thalamus (see above). The output of the laser was controlled by a waveform generator (Keysight technologies, CA, USA). Optical stimulation with 5 s epoch light on (473 nm, 25–35 mW) and 5 s epoch light off were alternately applied for 5 min during EEG recordings in freely moving mice. The efficacy of optical stimulation was measured at the end of the optical fibers before connecting to the animals.

### Behavior test

For the open field test, a 40 × 40 × 40 cm open box made from clear plexiglass was used. The open field box was divided into the peripheral zone and the central zone (20 × 20 cm). Each subject mouse will be given 10 min (5 min in optogenetic experiments) to freely explore the box. The total travel distance, mean speed, mobile and immobile time, freezing time and time spent in each zone were recorded. Optical stimulation with 5 s epoch light on (473 nm, 25–35 mW) and 5 s epoch light off were alternately applied during open field test in ChR2-expressing mice. For the ETX treatment, tests were done 30 min prior to and after the systemic or local injection. Data were analyzed using ANY-maze software (IL, USA).

The elevated plus maze was composed of two open arms (39.5 × 5 cm) and two closed arms of the same size with 10 cm high walls. The apparatus was placed 50 cm above the ground. The tested mice were individually placed in the center and allow for 10 min free exploration. The entries to and time spent in the open arms, center zone and closed arms were recorded. For the ETX treatment, tests were done 30 min before and after the IP injection. Data were analyzed using ANY-maze software (IL, USA).

For the five-trial social interaction test, the subject mouse was placed in a chamber (45 cm long × 20 cm wide) and presented with a same sex juvenile, strange mouse in a cylindrical wired cage (8 cm diameter, 17 cm high) with bars spaced 1 cm apart. Six consecutive 1 min trials with a 30–45 s inter-trial interval were tested for each subject. On the last trial, a novel stranger juvenile mouse of the same sex was presented in the cage. The amount of interaction was recorded as the sniff time when the animal oriented its nose within 2 cm of the stranger mouse in the wired cage. The normalized baseline values were calculated by dividing the amount of interaction in each trial (2–6) to that of trial 1. Data were analyzed using ANY-maze software (IL, USA).

The apparatus for the social recognition test consisted of three chambers (20 cm long × 45 cm wide × 30 cm high) connected by removable partitions in the plexiglass walls, which allowed animals to freely moving between the chambers. Mice were handled twice a day, 3 days before the test. Prior to the day of test, the handled mice were each habituated to the empty apparatus for 10 min. During the test, stranger mice were contained in a cylindrical wired cage (8 cm diameter, 17 cm high) with bars spaced 1 cm apart placed in left and/or right chamber. The middle chamber was left empty all the time. Each test session consisted of three stages: stage 1: 10 min habituation stage with two empty cages; stage 2: 5 min sociability test with an unencountered stranger mouse (S1) and an empty cage; stage 3: 5 min social memory test with the previously encountered stranger (S1) and a second novel stranger (S2). Each stage was separated by a 45 s–1 min interval. The amount of interaction was recorded using sniff time when the animal oriented its nose within 2 cm of the mouse contained in the wired cage. Data were analyzed as a percentage time spent investigating the target cage over the total time interacting with either cage using ANY-maze software (IL, USA).

For the novel object recognition test, a 40 × 40 × 40 cm clear Plexiglass box (same as the one used in open field test) was used. Two objects were placed 10 cm from the wall in the two diagonal corners of the clear Plexiglass box. Test mice were placed in the middle of the box and allowed to explore two identical objects (object 1 and object 2) for 10 min in stage 1 (habituation stage). Mice were then placed in the same clear Plexiglass box with the familiar object 1 and a novel object 3 for 5 min in stage 2. Two stages were separated by a 45 s–1 min interval. Data were analyzed as a percentage time spent investigating a particular object over the total time interacting with either objects using ANY-maze software (IL, USA).

### Slice electrophysiology

The mouse brains were quickly removed and immersed in ice-cold slicing solution saturated with 95% O_2_/5% CO_2_ containing (in mM) 75 sucrose, 120 NaCl, 2.5 KCl, 1.3 MgSO_4_, 1.0 NaH_2_PO_4_, 26 NaHCO_3_, 1.25 CaCl_2_, and 11 D-glucose. Horizontal 350 um thalamic slices containing the ventrobasal thalamus and nRT were prepared using a vibratome (VT 1200 s, Leica, Concord, ON, CA). Slices were recovered at 32 °C for 45 min to 1 h and then stored at room temperature, in ACSF saturated with 95% O_2_/5% CO_2_ containing (in mM): 120 NaCl, 3.0 KCl, 1.2 MgSO_4_, 1.0 NaH_2_PO_4_, 26 NaHCO_3_, 2.0 CaCl_2_, and 11 D-glucose. A single slice was transferred to a submersion chamber perfused with 95% O_2_/5% CO_2_ saturated ACSF. Perfusion flow rate was at 2 ml per min. The cortical, nRT, and thalamic neurons were visualized using an infrared differential interference contrast microscope (Zeiss Axioscope 2). All recordings were done with glass pipettes (3–4 MΩ) filled with intracellular solution. For sEPSC recordings, the intracellular solution contained (in mM) 130 CsMeSO_4_, 5 NaCl, 1 MgCl_2_, 0.05 EGTA, 10 HEPES, 3 Mg-ATP, 0.3 Na_3_-GTP, and 5 QX-314 (pH 7.25; 280-300 mOsm) and cells were clamped at −70 mV and recorded with 100 uM picrotoxin throughout the experiments. For IPSC recordings, the intracellular solution contained (in mM) 125 KCl, 1 MgCl_2_, 0.02 EGTA, 10 HEPES, 3 Mg-ATP and 0.5 Na3-GTP (pH 7.25) (280-300 mOsm) and cells were clamped at −70 mV and recorded with 10 μM NBQX and 50 μM APV. One micromolar TTX was added during mIPSC recordings. Cell series resistance was monitored by applying a −3 mV voltage step at the end of response and the experiment was excluded from analysis if the resistance changed more than 20%. Intrinsic firing properties were recorded in a current clamp mode with current injections (1 s, 20 pA steps from −300 pA to 180 pA, 10 s intervals), with an initial holding potential at −60 mV. The number of action potentials was measured for each depolarizing or hyperpolarizing current step. For optogenetic experiments, IPSCs were evoked by blue-light pulses (455 nm, 1–4 mW) delivered using a collimated LED (Thorlabs, Newton, NJ, USA) through a ×40 objective lens. Optical stimulation with 5 s epoch light on and 5 s epoch light off was alternately applied during spontaneous recordings in slices prepared from ChR2-expressing mice. The recorded cortical neurons were from the layer 5/6 of the somatosensory cortex and the recorded thalamic neurons were from the VPL/VPM region of the ventrobasal thalamus. Sample slice images were collected on a Leica M205 FA microscope using the LAS-X (Leica Microsystems, Buffalo Grove, IL, USA). All data acquisition and analysis were done using pClamp 10.6 (Clampex), pClampfit (Axon Instruments, Sunnyvale, CA, USA) and MiniAnalysis (Synaptosoft, Fort Lee, NJ, USA). In all electrophysiological experiments, *n* represents the number of neurons or slices and at most two slices per animal were used.

### Histology and immunohistochemistry

Mice were anesthetized using 10% chloral hydrate and perfused with 1 × phosphate-buffered saline (PBS) followed by 4% paraformaldehyde (PFA). Each brain was dissected and postfixed in 4% PFA for 10 h, and then transferred to 30% sucrose in PBS solution until it was fully saturated. The brain was sliced to 20 μm coronal cryostat sections at −20 °C (Leica CM1950, Concord, ON, CA). For cresyl violet staining, 0.5% cresyl violet acetate (Sigma–Aldrich, ON, CA) was dissolved in distilled water, added glacial acetic acid (Sigma–Aldrich, ON, CA). The sections were incubated in 70% ethanol bath for 5 min and washed twice before and after 20 min cresyl violet staining. The washed sections were then incubated in a serial (95%, 95%, 100%, 100%) ethanol baths in succession for dehydration. For immunohistochemistry staining, sections were washed with PBS, incubated in blocking solution (0.3% Triton, 5% BSA in 1 × PBS) for 1 h, incubated with primary antibodies overnight at 4 °C, washed with PBS and then secondary antibodies (dissolved in 0.05% Triton, 5% BSA in 1 × PBS) at room temperature for 2 h. The stained coverslips were mounted to coverslips using the VectaShield hard-set antifade mounting medium containing DAPI (Vector, Burlingame, CA, USA). Images were collected on a Nikon A1R confocal microscope using the NIS-Elements and analyzed using NIS-Element Viewer (Nikon Instruments, Melville, NY, USA) and ImageJ (NIH). Primary antibodies used for immunohistochemical analyses included anti-GFP (Aves Lab, 1:500), anti-Neuroligin 2 (Synaptic System, 1:500), anti-Parvalbumin (Sigma–Aldrich, 1:1000), anti-GFAP (Cell Signaling Technology, 1:500). Neurotrace (Thermo Fisher Scientific, 1:250) was added in conjunction with corresponding secondary antibodies. The following secondary antibodies were used: donkey anti-chicken IgG, Alexa Fluor 488 (Jackson ImmunoResearch Laboratories, 1:1000), donkey anti-rabbit IgG, Alexa Fluor 568 (Invitrogen, 1:1000), goat anti-mouse IgG, Alexa Fluor 488 (Invitrogen, 1:1000), and goat anti-mouse IgG, Alexa Fluor 546 (Invitrogen, 1:1000).

### Statistical analyses

All the data in the figures were presented as mean ± SEM and statistically evaluated by independent two-tailed *t*-tests, paired *t*-tests, one-way ANOVA, two-way ANOVA or repeated measures two-way ANOVA, wherever appropriate, followed by Post-hoc Holm–Sidak’s multiple comparisons. *p* < 0.05 was considered as significant and indicated with *p < 0.05, **p < 0.01, ***p < 0.001. Independent sample size (*n*) represented number of animals in EEG recordings, behavioral tests and western blot, and number of neurons in electrophysiological recordings. All analyses were conducted using GraphPad PRISM (GraphPad Software, La Jolla, CA, USA) and SigmaPlot (Systat Software Inc., San Jose, CA, USA).

### Reporting summary

Further information on research design is available in the Nature Research Reporting Summary linked to this article.

## Supplementary information

Supplementary Information

Peer Review File

Reporting Summary

## Data Availability

All relevant data in this study are included within the article and supplementary information. The source data underlying Figs. 1e–h, 2a–h, 3c, f, g, j, k, 4a, b, d, e, g, h, j, k, 5a–e, 6f–m and 7d, h–l and Supplementary Figs. 1a, c–f, 3b, c, 4c, e, f, 5c–f, 6b–d, 7a, b, 8a, b, 9a, b, 10a–e, 11a, b and 13b, c are provided as a Source Data file. Source Data are provided with the paper. All requests for raw data and materials are promptly reviewed by the Hospital for Sick Children to verify whether the request is subject to any intellectual property or confidentiality obligations. Any materials that can be shared will be released via a Material Transfer Agreement. Source data are provided with this paper.

## Source data

Source Data

## References

[1] Gyawali, S. & Patra, B.N. Autism spectrum disorder: trends in research exploring etiopathogenesis. *Psychiatry Clin. Neurosci*. 10.1111/pcn.12860 (2019). 10.1111/pcn.12860 31077508

[2] Sanchack KE, Thomas CA (2016). Autism spectrum disorder: primary care principles. Am. Fam. Physician.

[3] Belmonte MK, Bourgeron T (2006). Fragile X syndrome and autism at the intersection of genetic and neural networks. Nat. Neurosci..

[4] Cortesi F, Giannotti F, Ivanenko A, Johnson K (2010). Sleep in children with autistic spectrum disorder. Sleep. Med..

[5] Ghaziuddin M, Ghaziuddin N, Greden J (2002). Depression in persons with autism: implications for research and clinical care. J. Autism Dev. Disord..

[6] Jang J (2013). Rates of comorbid symptoms in children with ASD, ADHD, and comorbid ASD and ADHD. Res Dev. Disabil..

[7] Mannion A, Leader G (2014). Epilepsy in autism spectrum disorder. Res. Autism Spectr. Disord..

[8] Lee BH, Smith T, Paciorkowski AR (2015). Autism spectrum disorder and epilepsy: disorders with a shared biology. Epilepsy Behav..

[9] Jeste SS (2011). The neurology of autism spectrum disorders. Curr. Opin. Neurol..

[10] Spence SJ, Schneider MT (2009). The role of epilepsy and epileptiform EEGs in autism spectrum disorders. Pediatr. Res..

[11] Frye RE (2016). Neuropathological mechanisms of seizures in autism spectrum disorder. Front. Neurosci..

[12] Polsek D, Jagatic T, Cepanec M, Hof PR, Simic G (2011). Recent developments in neuropathology of autism spectrum disorders. Transl. Neurosci..

[13] Rubenstein JL, Merzenich MM (2003). Model of autism: increased ratio of excitation/inhibition in key neural systems. Genes Brain Behav..

[14] Sudhof TC (2008). Neuroligins and neurexins link synaptic function to cognitive disease. Nature.

[15] Eichler SA, Meier JC (2008). E-I balance and human diseases—from molecules to networking. Front. Mol. Neurosci..

[16] McCormick DA, Contreras D (2001). On the cellular and network bases of epileptic seizures. Annu. Rev. Physiol..

[17] Maheshwari A, Noebels JL (2014). Monogenic models of absence epilepsy: windows into the complex balance between inhibition and excitation in thalamocortical microcircuits. Prog. Brain Res..

[18] Fogerson PM, Huguenard JR (2016). Tapping the brakes: cellular and synaptic mechanisms that regulate thalamic oscillations. Neuron.

[19] Dean C, Dresbach T (2006). Neuroligins and neurexins: linking cell adhesion, synapse formation and cognitive function. Trends Neurosci..

[20] Lise MF, El-Husseini A (2006). The neuroligin and neurexin families: from structure to function at the synapse. Cell Mol. Life Sci..

[21] Etherton M (2011). Autism-linked neuroligin-3 R451C mutation differentially alters hippocampal and cortical synaptic function. Proc. Natl Acad. Sci. USA.

[22] Sun C (2011). Identification and functional characterization of rare mutations of the neuroligin-2 gene (NLGN2) associated with schizophrenia. Hum. Mol. Genet..

[23] Parente DJ (2017). Neuroligin 2 nonsense variant associated with anxiety, autism, intellectual disability, hyperphagia, and obesity. Am. J. Med. Genet. A.

[24] Baudouin SJ (2012). Shared synaptic pathophysiology in syndromic and nonsyndromic rodent models of autism. Science.

[25] Radyushkin K (2009). Neuroligin-3-deficient mice: model of a monogenic heritable form of autism with an olfactory deficit. Genes Brain Behav..

[26] Tabuchi K (2007). A neuroligin-3 mutation implicated in autism increases inhibitory synaptic transmission in mice. Science.

[27] Poulopoulos A (2009). Neuroligin 2 drives postsynaptic assembly at perisomatic inhibitory synapses through gephyrin and collybistin. Neuron.

[28] Liang J (2015). Conditional neuroligin-2 knockout in adult medial prefrontal cortex links chronic changes in synaptic inhibition to cognitive impairments. Mol. Psychiatry.

[29] Babaev O (2016). Neuroligin 2 deletion alters inhibitory synapse function and anxiety-associated neuronal activation in the amygdala. Neuropharmacology.

[30] Gibson JR, Huber KM, Sudhof TC (2009). Neuroligin-2 deletion selectively decreases inhibitory synaptic transmission originating from fast-spiking but not from somatostatin-positive interneurons. J. Neurosci..

[31] Chubykin AA (2007). Activity-dependent validation of excitatory versus inhibitory synapses by neuroligin-1 versus neuroligin-2. Neuron.

[32] Jedlicka P (2011). Increased dentate gyrus excitability in neuroligin-2-deficient mice in vivo. Cereb. Cortex.

[33] Hines RM (2008). Synaptic imbalance, stereotypies, and impaired social interactions in mice with altered neuroligin 2 expression. J. Neurosci..

[34] Crunelli V, Leresche N (2002). Childhood absence epilepsy: genes, channels, neurons and networks. Nat. Rev. Neurosci..

[35] Kostopoulos GK (2001). Involvement of the thalamocortical system in epileptic loss of consciousness. Epilepsia.

[36] Paz JT (2011). A new mode of corticothalamic transmission revealed in the Gria4(-/-) model of absence epilepsy. Nat. Neurosci..

[37] Bernardo KL, Woolsey TA (1987). Axonal trajectories between mouse somatosensory thalamus and cortex. J. Comp. Neurol..

[38] Pinault D, Bourassa J, Deschenes M (1995). The axonal arborization of single thalamic reticular neurons in the somatosensory thalamus of the rat. Eur. J. Neurosci..

[39] Zhang L, Jones EG (2004). Corticothalamic inhibition in the thalamic reticular nucleus. J. Neurophysiol..

[40] Bertaso F (2008). PICK1 uncoupling from mGluR7a causes absence-like seizures. Nat. Neurosci..

[41] Blundell J (2009). Increased anxiety-like behavior in mice lacking the inhibitory synapse cell adhesion molecule neuroligin 2. Genes Brain Behav..

[42] Beyenburg S, Mitchell AJ, Schmidt D, Elger CE, Reuber M (2005). Anxiety in patients with epilepsy: systematic review and suggestions for clinical management. Epilepsy Behav..

[43] Jackson MJ, Turkington D (2005). Depression and anxiety in epilepsy. J. Neurol. Neurosurg. Psychiatry.

[44] Vazquez B, Devinsky O (2003). Epilepsy and anxiety. Epilepsy Behav..

[45] White SW, Roberson-Nay R (2009). Anxiety, social deficits, and loneliness in youth with autism spectrum disorders. J. Autism Dev. Disord..

[46] Leung C (2018). Activation of entorhinal cortical projections to the dentate gyrus underlies social memory retrieval. Cell Rep..

[47] Kazdoba TM, Leach PT, Silverman JL, Crawley JN (2014). Modeling fragile X syndrome in the Fmr1 knockout mouse. Intractable Rare Dis. Res..

[48] Berry-Kravis E (2002). Epilepsy in fragile X syndrome. Dev. Med. Child Neurol..

[49] Dimidschstein J (2016). A viral strategy for targeting and manipulating interneurons across vertebrate species. Nat. Neurosci..

[50] Nelson SB, Valakh V (2015). Excitatory/inhibitory balance and circuit homeostasis in autism spectrum disorders. Neuron.

[51] Varoqueaux F, Jamain S, Brose N (2004). Neuroligin 2 is exclusively localized to inhibitory synapses. Eur. J. Cell Biol..

[52] Bena F (2013). Molecular and clinical characterization of 25 individuals with exonic deletions of NRXN1 and comprehensive review of the literature. Am. J. Med. Genet. B Neuropsychiatr. Genet..

[53] Strauss KA (2006). Recessive symptomatic focal epilepsy and mutant contactin-associated protein-like 2. N. Engl. J. Med..

[54] Penagarikano O (2011). Absence of CNTNAP2 leads to epilepsy, neuronal migration abnormalities, and core autism-related deficits. Cell.

[55] Fernandez T (2004). Disruption of contactin 4 (CNTN4) results in developmental delay and other features of 3p deletion syndrome. Am. J. Hum. Genet..

[56] Roohi J (2009). Disruption of contactin 4 in three subjects with autism spectrum disorder. J. Med. Genet..

[57] Karayannis T (2014). Cntnap4 differentially contributes to GABAergic and dopaminergic synaptic transmission. Nature.

[58] Bolkan SS (2017). Thalamic projections sustain prefrontal activity during working memory maintenance. Nat. Neurosci..

[59] Halassa MM, Kastner S (2017). Thalamic functions in distributed cognitive control. Nat. Neurosci..

[60] Schmitt LI, Halassa MM (2017). Interrogating the mouse thalamus to correct human neurodevelopmental disorders. Mol. Psychiatry.

[61] Krol A, Wimmer RD, Halassa MM, Feng G (2018). Thalamic reticular dysfunction as a circuit endophenotype in neurodevelopmental disorders. Neuron.

[62] Nair A, Treiber JM, Shukla DK, Shih P, Muller RA (2013). Impaired thalamocortical connectivity in autism spectrum disorder: a study of functional and anatomical connectivity. Brain.

[63] Schuetze M (2016). Morphological alterations in the thalamus, striatum, and pallidum in autism spectrum disorder. Neuropsychopharmacology.

[64] Woodward ND, Giraldo-Chica M, Rogers B, Cascio CJ (2017). Thalamocortical dysconnectivity in autism spectrum disorder: an analysis of the Autism Brain Imaging Data Exchange. Biol. Psychiatry Cogn. Neurosci. Neuroimaging.

[65] Wells MF, Wimmer RD, Schmitt LI, Feng G, Halassa MM (2016). Thalamic reticular impairment underlies attention deficit in Ptchd1(Y/-) mice. Nature.

[66] Richards DA (2003). Targeting thalamic nuclei is not sufficient for the full anti-absence action of ethosuximide in a rat model of absence epilepsy. Epilepsy Res..

[67] Meeren HK, Pijn JP, Van Luijtelaar EL, Coenen AM, Lopes da Silva FH (2002). Cortical focus drives widespread corticothalamic networks during spontaneous absence seizures in rats. J. Neurosci..

[68] Polack PO (2007). Deep layer somatosensory cortical neurons initiate spike-and-wave discharges in a genetic model of absence seizures. J. Neurosci..

