# Supplementary material for: Neuroligin 2 regulates absence seizures and behavioral arrests through GABAergic transmission within the thalamocortical circuitry
Source: Nat Commun. 2020 Jul 27;11:3744. doi: 10.1038/s41467-020-17560-3 (PMC7385104; doi:10.1038/s41467-020-17560-3)
Supplement: Supplementary file 1 — Supplementary Information [file 41467_2020_17560_MOESM1_ESM.pdf]

# Supplementary Information

**“Neuroligin 2 regulates absence seizures and behavioral arrests through GABAergic transmission within the thalamocortical circuitry” by Cao et al.**

**Supplementary Fig. 1**

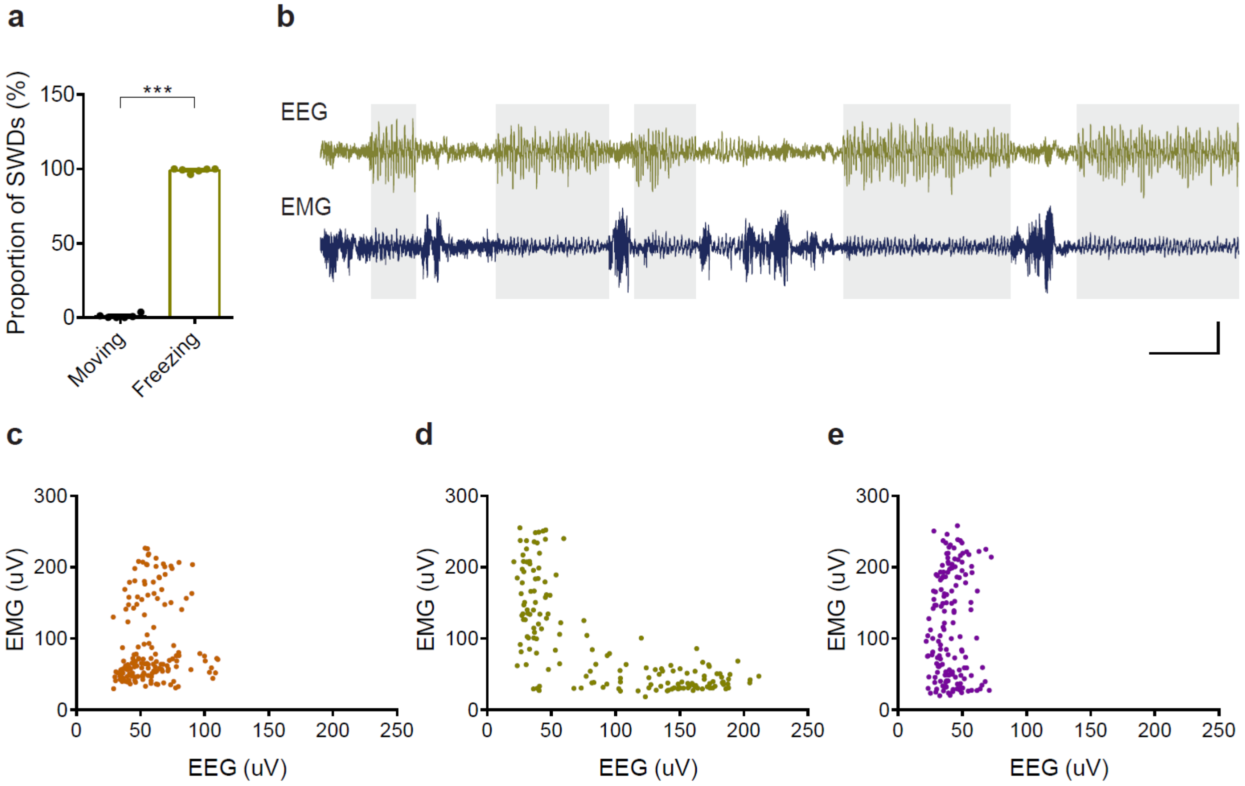

**Supplementary Fig. 1. Correlation analysis of EEG and EMG recordings and behavior arrests**

**a**, Summary graph showing the proportion of behavior moving and freezing associated with SWDs in NLG2 KO mice ($n = 6$, two-sided t test, $p < 0.0001$). Moving refers to any whole-body movement. Freezing refers to lack of any whole-body movement but including minor head movement.

**b**, Representative EEG and EMG traces showing the correlation of SWDs and behavior arrests (shaded areas) in NLG2 KO mice. Scale bar: 3 s/100 μV.

**c**, Correlation analysis showing no significant correlation between EEG and EMG amplitude in NLG1 KO mice (Pearson's correlation analysis, $r = 0.0956$, $p = 0.2293$, XY pairs = 160).

**d**, Correlation analysis showing significant correlation between EEG and EMG amplitude in NLG2 KO mice (Pearson's correlation analysis, $r = -0.7215$, $p < 0.0001$, XY pairs = 160).

**e**, Correlation analysis showing no significant correlation between EEG and EMG amplitude in NLG3 KO mice (Pearson's correlation analysis, $r = 0.0815$, $p = 0.3056$, XY pairs = 160).

Data in **a** represent mean ± SEM. ***$p < 0.001$. Source data are provided as a Source Data file.

**Supplementary Fig. 2.**

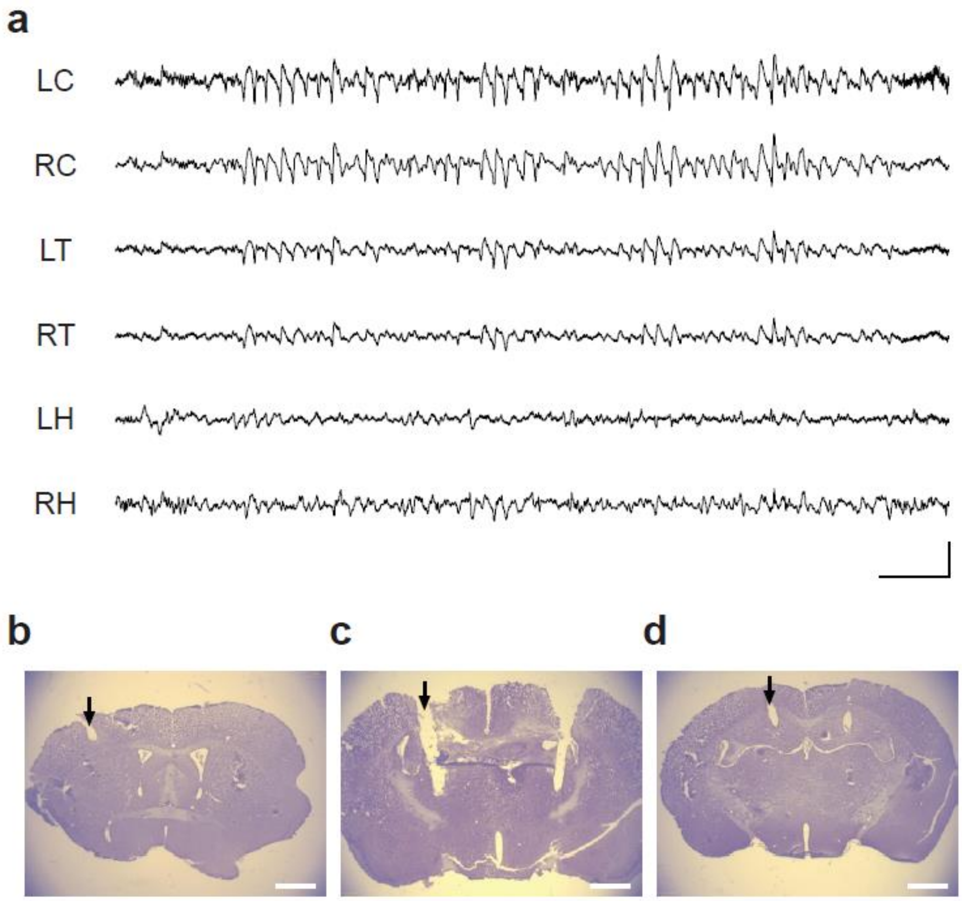


**Supplementary Fig. 2. EEG recordings in different brain regions**

**a**, Sample EEG recordings showing SWDs in the cortex and thalamus, but not in the hippocampus. LC: left cortex, RC: right cortex, LT: left thalamus, RT: right thalamus, LH: left hippocampus; RH, right hippocampus. Scale bar: 1 s/200 μV. Recordings traces are representatives of 4 replicates.

**b-d**, Cresyl violet staining showing the track of electrodes for recording the cortex (**b**), thalamus (**c**) and hippocampus (**d**), respectively. Scale bars: 1 mm. Images are representatives of 4 replicates.

**Supplementary Fig. 3**

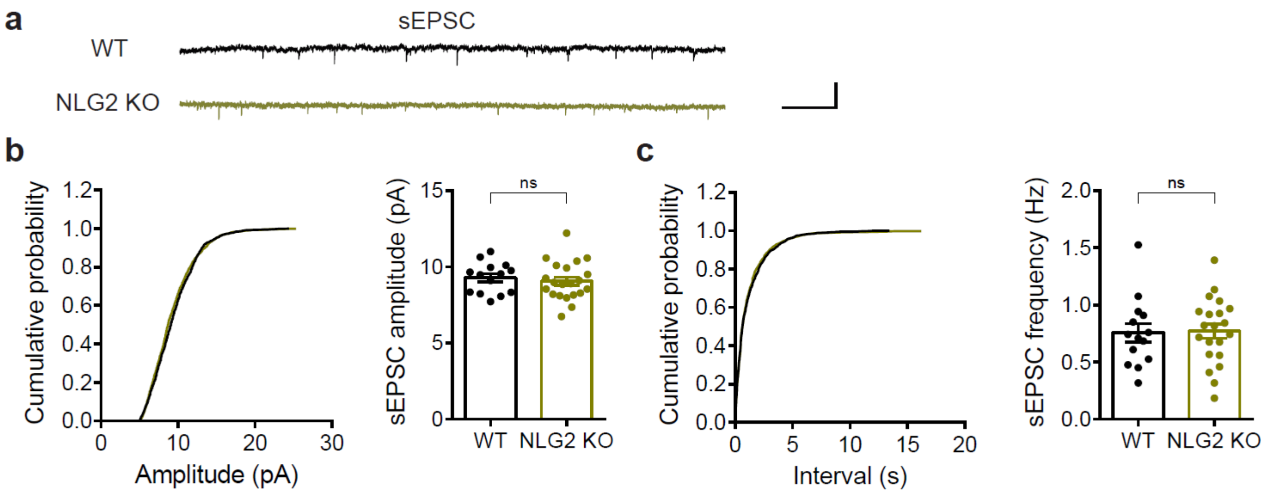


**Supplementary Fig. 3. Normal sEPSC in thalamic neurons in NLG2 KO mice**

**a**, Sample traces of sEPSC in WT and NLG2 KO thalamic neurons. Scale bar: 1 s/20 pA.

**b**, Cumulative probability histograms and summary graphs showing no differences in sEPSC amplitude ($p = 0.5870$) between WT ($n = 14$) and NLG2 KO ($n = 21$) thalamic neurons.

**c**, Cumulative probability histograms and summary graphs showing no differences in sEPSC frequency ($p = 0.8854$) between WT ($n = 14$) and NLG2 KO ($n = 21$) thalamic neurons.

Data represent mean ± SEM. ns = non-significant. Two-sided t test was used for **b** and **c**. Source data are provided as a Source Data file.

**Supplementary Fig. 4**

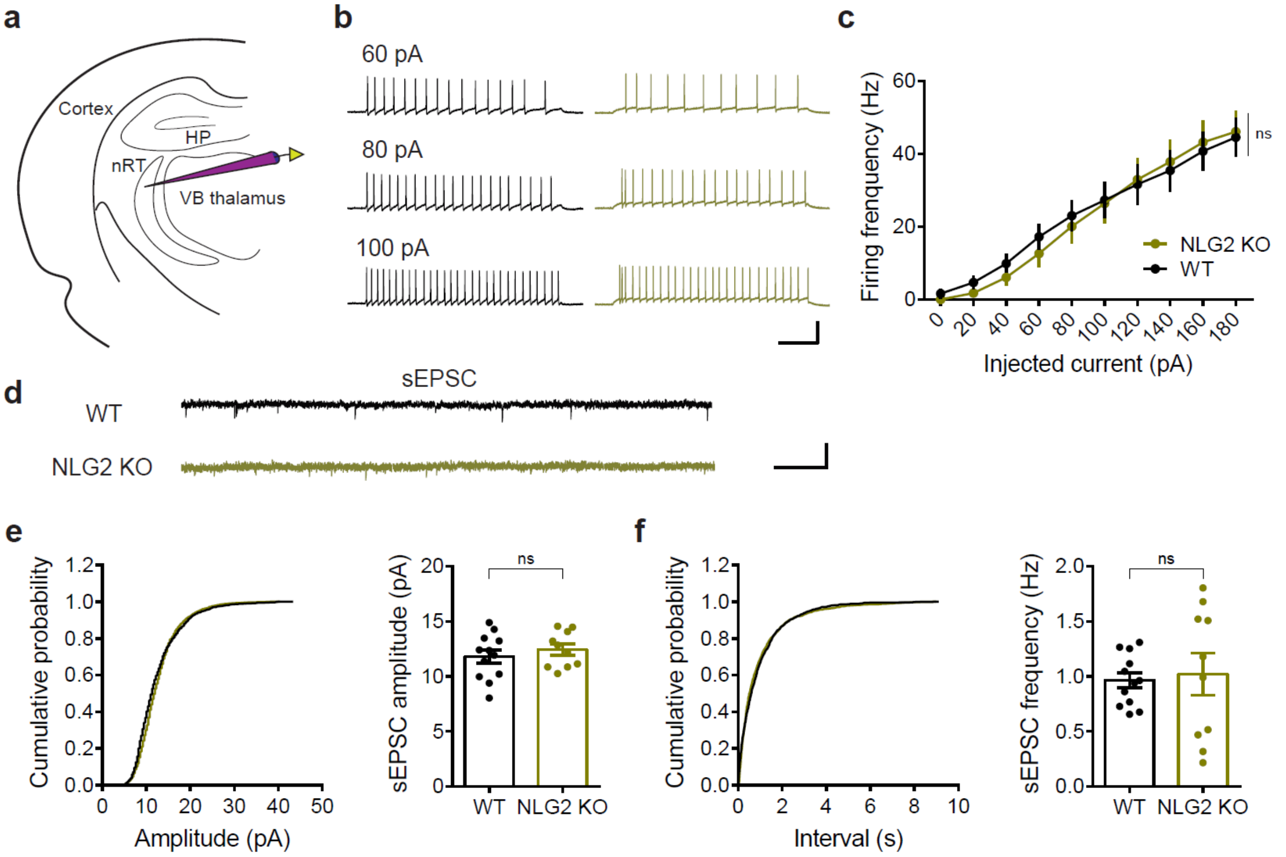

**Supplementary Fig. 4. Normal intrinsic excitabilities and sEPSC in nRT neurons in NLG2 KO mice**

**a**, Positioning of the whole-cell recording electrode in the nRT.

**b**, Representative traces showing responses to 60, 80 and 100 pA of depolarizing current injections in WT (left) and NLG2 KO (right) nRT neurons initially held at -60 mV. Scale bar: 0.2 s/60 mV.

**c**, Summary graph showing spike frequency in response to various depolarizing currents. No difference was seen between WT (n = 10) and NLG2 KO (n = 13) mice (two-way ANOVA, p = 0.6597).

**d**, Sample traces of sEPSC in WT and NLG2 KO nRT neurons. Scale bar: 1 s/20 pA.

**e**, Cumulative probability histograms and summary graph of sEPSC events showing no differences in sEPSC amplitude (p = 0.3081) between WT (n = 14) and NLG2 KO (n = 10) nRT neurons.

**f**, Cumulative probability histograms and summary graph of sEPSC events showing no differences in sEPSC frequency (p = 0.5358) between WT (n = 14) and NLG2 KO (n = 10) nRT neurons.

Data in **c**, **e** and **f** represent mean ± SEM. ns = non-significant. Two-sided t -test was used for **e** and **f**. Source data are provided as a Source Data file.

**Supplementary Fig. 5**

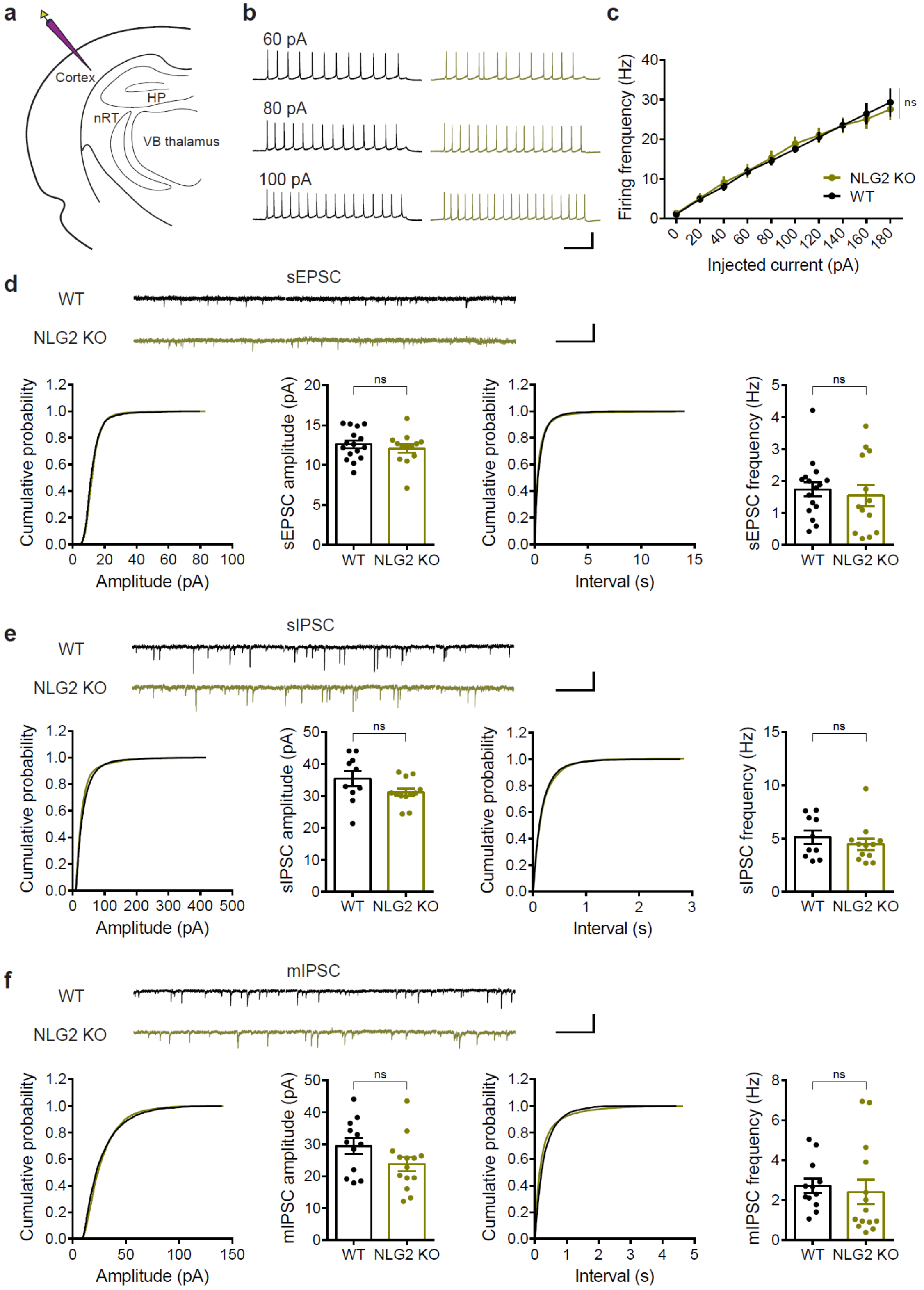

**Supplementary Fig. 5. Normal intrinsic excitabilities and synaptic transmission in the cortical layer 5/6 neurons**

**a**, Positioning of the whole-cell recording electrode in the somatosensory cortex.

**b**, Representative traces showing responses to 60, 80 and 100 pA of depolarizing current injections in WT (left) and NLG2 KO (right) cortical neurons initially held at -60 mV. Scale bar: 0.2 s/60 mV.

**c**, Summary graph showing spike frequency in response to various depolarizing currents. No difference was seen between WT (n = 13) and NLG2 KO (n = 12) mice (two-way ANOVA, p = 0.8908).

**d**, Sample traces, cumulative probability histograms and summary graphs of sEPSC events showing no differences in sEPSC amplitude (p = 0.9169) and frequency (p = 0.8327) between WT (n = 16) and NLG2 KO (n = 12) cortical neurons. Scale bar: 1 s/20 pA.

**e**, Sample traces, cumulative probability histograms and summary graph of sIPSC events showing no differences in sIPSC amplitude (p = 0.3101) and frequency (p = 0.4242) between WT (n = 12) and NLG2 KO (n = 15) cortical neurons. Scale bar: 1 s/20 pA.

**f**, Sample traces, cumulative probability histograms and summary graph of mIPSC events showing no differences in mIPSC amplitude (p = 0.2534) and frequency (p = 0.9776) between WT (n = 12) and NLG2 KO (n = 14) cortical neurons. Scale bar: 1 s/20 pA.

Data in **c-f** represent mean ± SEM. ns = non-significant. Two-sided t -test was used for **d-f**. Source data are provided as a Source Data file.

**Supplementary Fig. 6**

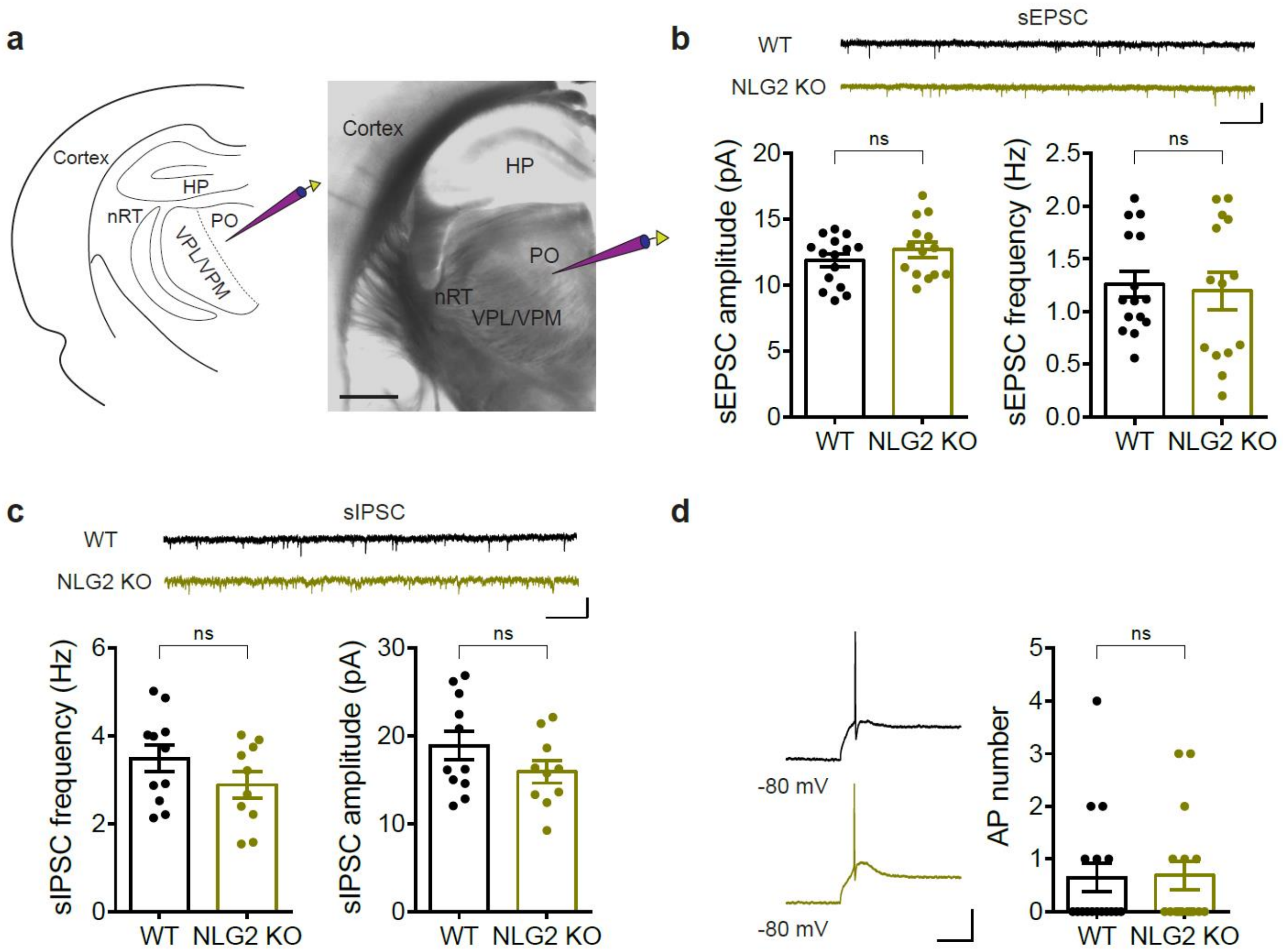

**Supplementary Fig. 6. Synaptic transmission and burst firing in the PO neurons of the thalamus**

**a**, Positioning of the whole-cell recording electrode in the PO region of the thalamus. Scale bar: 1 mm.

**b**, Sample traces and summary graphs showing no differences in sEPSC amplitude ($p = 0.2705$) and frequency ($p = 0.2897$) between WT ($n = 15$) and NLG2 KO ($n = 14$) PO neurons. Scale bar: 1 s/20 pA.

**c**, Sample traces and summary graph showing no differences in sIPSC amplitude ($p = 0.1737$) and frequency ($p = 0.1763$) between WT ($n = 11$) and NLG2 KO ($n = 10$) PO neurons. Scale bar: 1 s/20 pA.

**d**, Sampling traces and summary graph showing similar rebound bursting firing upon hyperpolarization at -80mV in WT ($n = 17$) and NLG2 KO ($n = 16$) PO neurons ($p = 0.9164$).

Data in **b-d** represent mean ± SEM. ns = non-significant. Two-sided t -test was used for **b-d**. Source data are provided as a Source Data file.

**Supplementary Fig. 7**

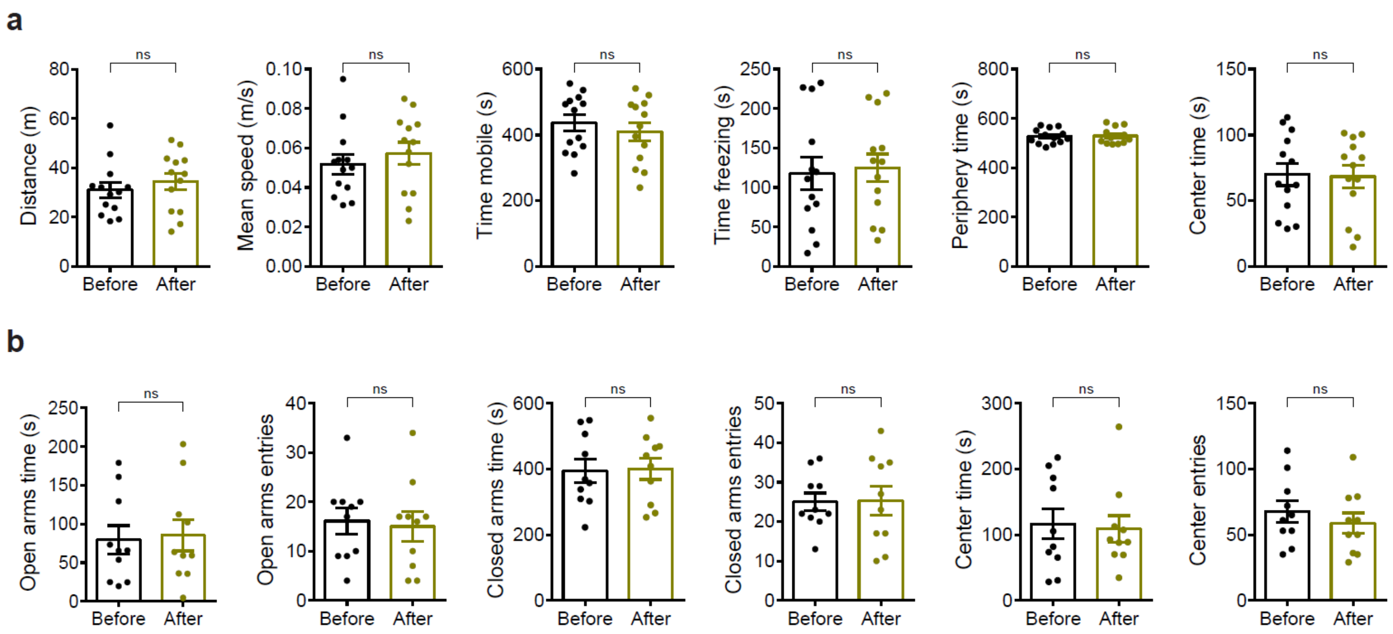

**Supplementary Fig. 7. Anti-absence seizure drug ETX has no effect on WT mice in the open field and elevated plus maze test**

**a**, Open field test showing similar travel distance (p = 0.4772), travel speed (p = 0.4833), mobile time (p = 0.5539), freezing time (p = 0.8073), time spent in periphery zone (p = 0.9018), and time spent in center zone (p = 0.8903) after systemic injections of ETX compared to before injections in WT mice (n = 13).

**b**, Elevated plus maze test showing similar time spent in open arms (p = 0.7839), open arm entries (p = 0.7961), time spent in closed arms (p = 0.8830), closed arm entries (p = 0.9291), time spent in center (p = 0.7794), and center entries (p = 0.4888) after systemic injections of ETX compared to before injections in WT mice (n = 10).

Data represent mean ± SEM. ns = non-significant. Two-sided t -test was used. Source data are provided as a Source Data file.

**Supplementary Fig. 8**

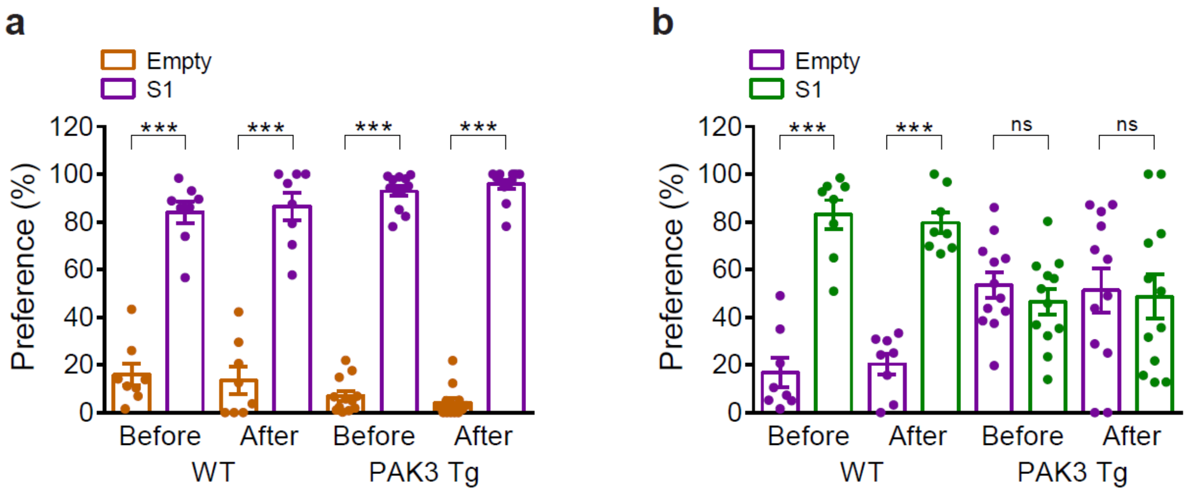


**Supplementary Fig. 8. Anti-absence seizure drug ETX has no effect on the impaired social recognition memory deficits in PAK3 transgenic mouse model**

**a**, Three-chamber social interaction test showing normal preference for S1 over empty cage during stage 2 before and after systemic injections of ETX in WT (before treatment: p = 0.0002, after treatment: p = 0.0003, n = 8) and PAK3 Tg (PAK3 transgenic) mice (before treatment: p < 0.0001, after treatment: p < 0.0001, n = 12).

**b**, Normal preference for S2 over S1 in WT and impaired preference for S2 over S1 in PAK3 Tg mice during stage 3 before and after ETX treatment (WT mice before treatment: p = 0.0009, after treatment: p = 0.0003, n = 8; PAK3 Tg mice before treatment: p = 0.5266, after treatment: p = 0.886, n = 12).

Data represent mean ± SEM. ***p < 0.001. ns = non-significant. Two-sided t -test was used. Source data are provided as a Source Data file.

**Supplementary Fig. 9**

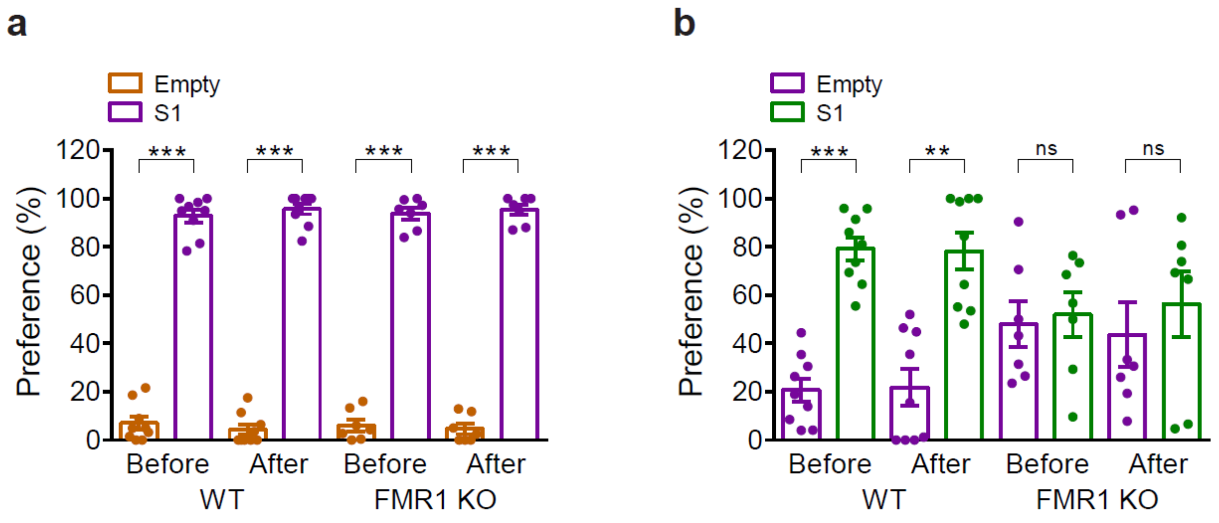


**Supplementary Fig. 9. Anti-absence seizure drug ETX has no rescuing effect on the impaired social recognition deficits in FMR1 KO mice**

**a**, Three-chamber social interaction test showing normal preference for S1 over empty cage during stage 2 before and after systemic injections of ETX in WT (before treatment: $p < 0.0001$, after treatment: $p < 0.0001$, $n = 9$) and FMR1 KO mice (before treatment: $p < 0.0001$; after treatment: $p < 0.0001$; $n = 7$).

**b**, Normal preference for S2 over S1 in WT and impaired preference for S2 over S1 in FMR1 KO mice during stage 3 before and after systemic injections of ETX (WT mice before treatment: $p = 0.0003$, after treatment: $p = 0.0058$, $n = 9$; FMR1 KO mice before treatment: $p = 0.8359$; after treatment: $p = 0.6543$, $n = 7$).

Data represent mean ± SEM. **$p < 0.01$, ***$p < 0.001$. ns = non-significant. Two-sided t test. Source data are provided as a Source Data file.

**Supplementary Fig. 10**

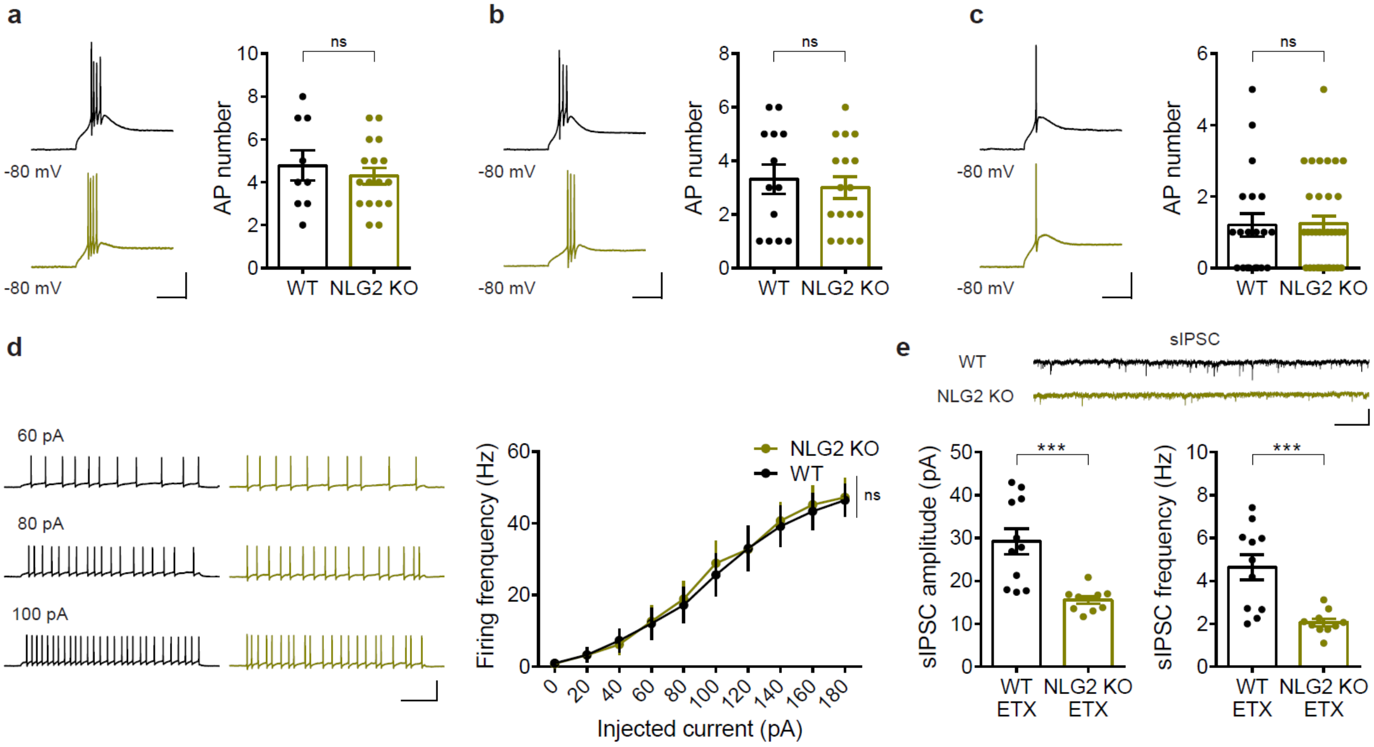

**Supplementary Fig. 10. Anti-absence seizure drug ETX reduces hyperpolarization induced burst firing, but has no effect on the impaired sIPSC in NLG2 KO thalamic neurons**

**a,** Sampling traces and summary graph showing similar number of action potentials in the rebound burst firing upon hyperpolarization at -80mV in WT (n = 9) and NLG2 KO (n = 17) nRT neurons (p = 0.5134). Scale bar: 100 ms/30 mV.

**b**, Sampling traces and summary graph showing similar number of action potentials in the rebound burst firing upon hyperpolarization at -80mV in WT (n = 13) and NLG2 KO (n = 17) thalamic neurons (p = 0.6464). Scale bar: 100 ms/30 mV.

**c**, Sampling traces and summary graph showing similar number of action potentials in the rebound burst firing after ETX treatment in WT (n = 20) and NLG2 KO (n = 34) thalamic neurons (p = 0.9259). Scale bar: 100 ms/30 mV. There is significant reduction in the number of action potentials in both WT (p = 0.0012) and NLG2 KO (p = 0.0001) neurons after the ETX treatment compared to no ETX treatment.

**d**, Representative traces and summary graph showing spike frequency in response to various depolarizing currents after ETX treatment in thalamic neurons. No difference was seen between WT (n = 11) and NLG2 KO (n = 10) mice (two-way ANOVA, p = 0.6898). Scale bar: 0.2 s/60 mV.

**e**, Summary graphs showing impaired sIPSC amplitude (p = 0.0005) and frequency (p = 0.0008) in NLG2 KO (n = 10) thalamic neurons compared to WT (n = 11) thalamic neurons after ETX treatment. Scale bar: 1 s/20 pA.

Data represent mean ± SEM. ***p < 0.001. ns = non-significant. Two-sided t test was used for **a-c** and **e**. Source data are provided as a Source Data file.

**Supplementary Fig. 11**

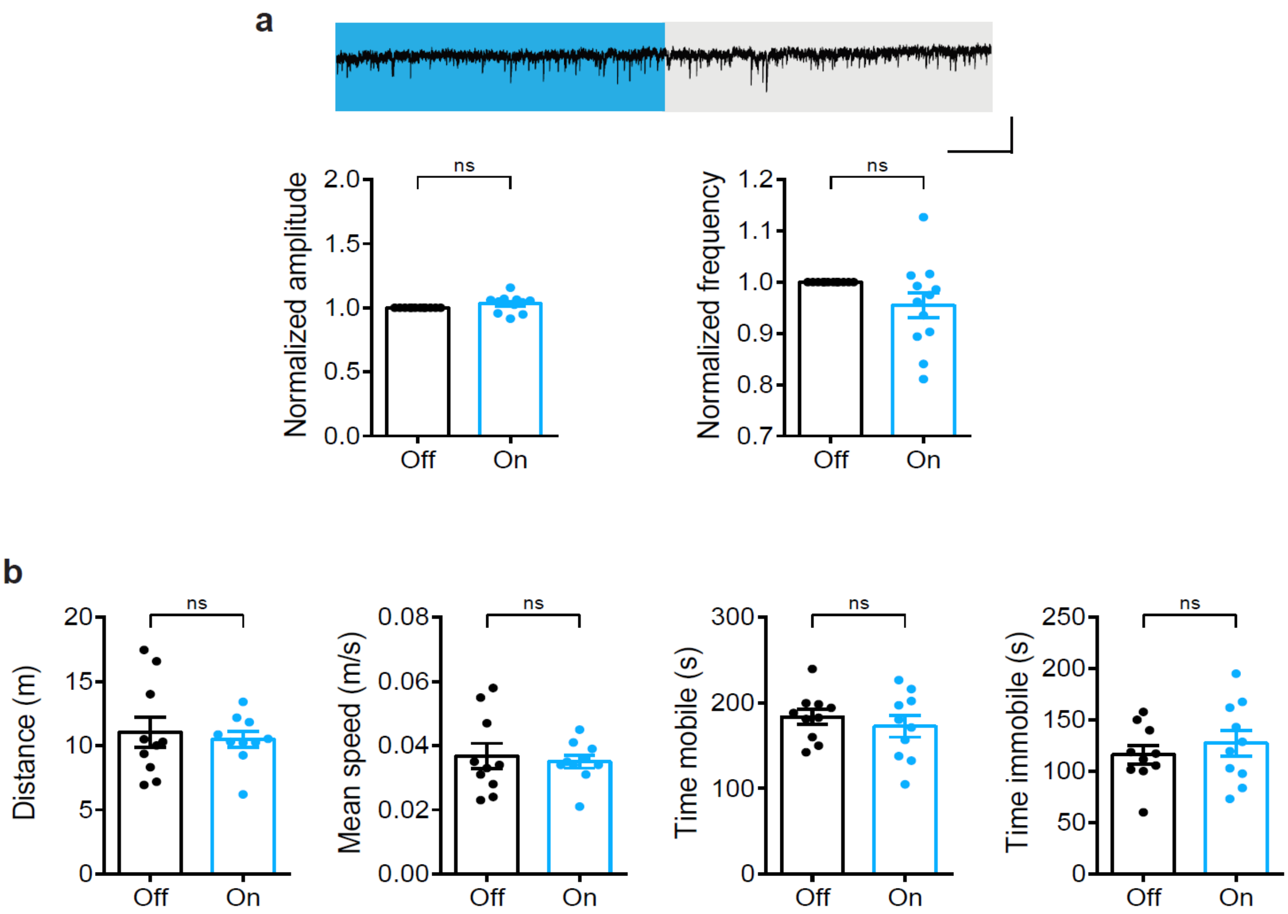

**Supplementary Fig. 11. Blue light illumination has no effect on the sIPSC and mobility behavior in WT mice without viral injections**

**a**, Sample traces and summary graph of sIPSC events showing similar sIPSC amplitude ($p = 0.1228$) and frequency ($p = 0.0916$) with blue-light on compared to blue-light off in WT mice ($n = 12$) with only optical fiber implantation. Scale bar: 1 s/20 pA.

**b**, Open field test showing similar travel distance ($p = 0.6108$), travel speed ($p = 0.6233$), mobile time ($p = 0.3025$) and immobile time ($p = 0.3023$) with alternate 5 s blue-light on and off compared to blue-light off in WT mice ($n = 10$) with only optical fiber implantation.

Data represent mean ± SEM. ns = non-significant. Two-sided t -test was used. Source data are provided as a Source Data file.

**Supplementary Fig. 12**

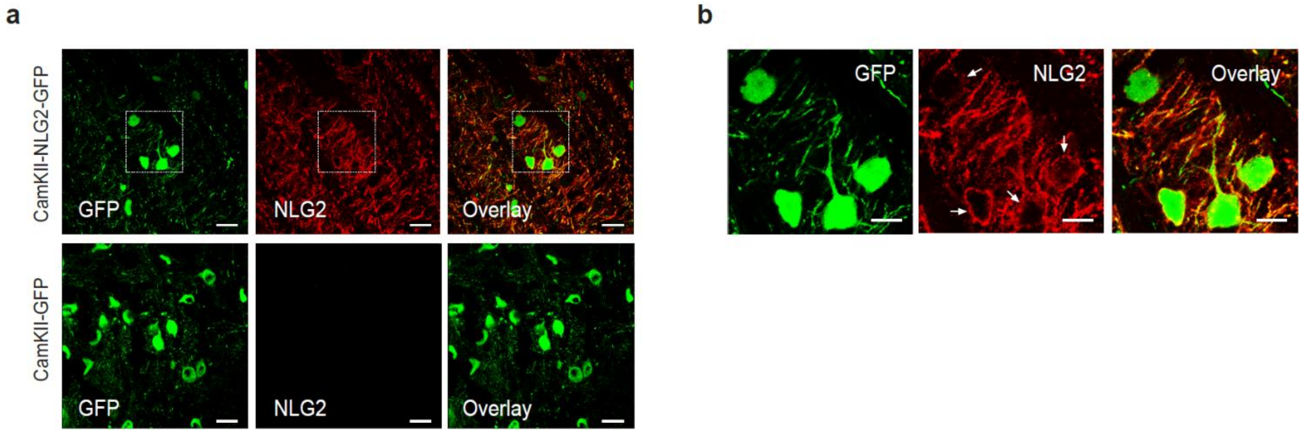


**Supplementary Fig. 12. Expression of CamKII-NLG2-GFP and CamKII-GFP virus in NLG2 KO mice**

**a**, Immunostaining images showing the expression of NLG2 (red) in CamKII-NLG2-GFP virus infected thalamic neurons compared to no NLG2 expression in control CamKII-GFP virus infected thalamic neurons in NLG2 KO mice. Scale bars: 20 μm. Images are representatives of 3 replicates.

**b**, High magnification images showing surface expression of NLG2 (red) in GFP+ thalamic neurons in NLG2 KO mice injected with CamKII-NLG2-GFP virus. Scale bars: 10 μm. Images are representatives of 3 replicates.

**Supplementary Fig. 13**

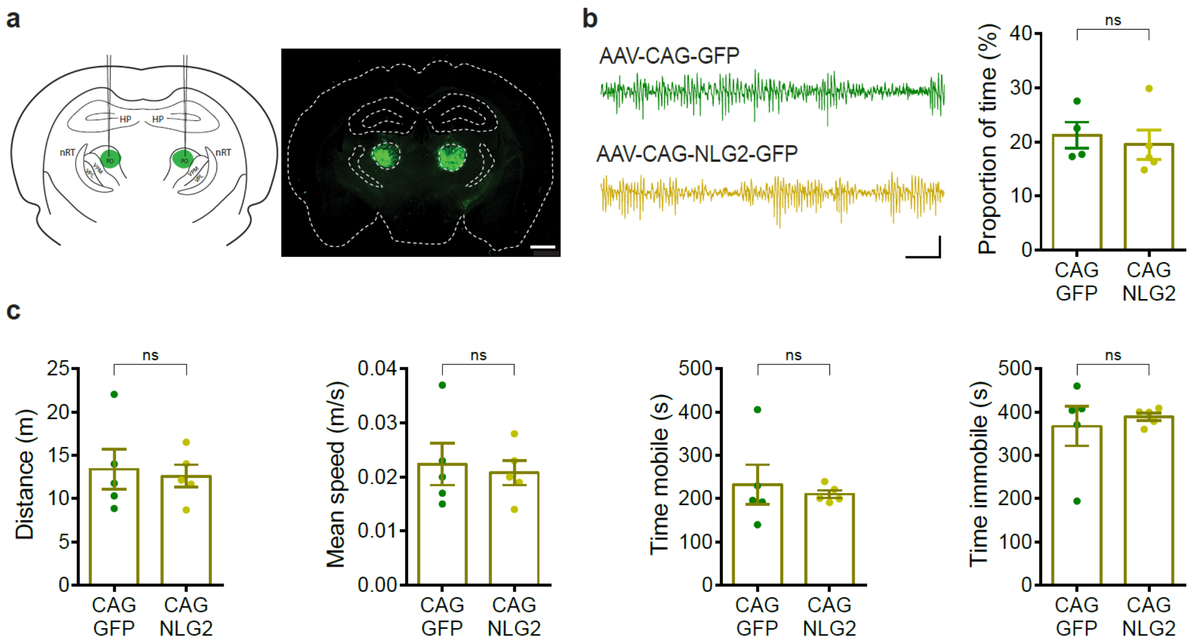


**Supplementary Fig. 13. Re-introducing NLG2 to the PO region of the thalamus does not reduce SWDs and behavior arrests in NLG2 KO mice**

**a**, Injection sites of AAV-NLG2-GFP expressing viruses in the PO region of the thalamus.

**b**, Representative EEG traces and summary graph of SWDs in NLG2 KO mice showing similar proportion of SWDs with the viral expression of CAG-NLG2-GFP (n = 5) and CAG-GFP control (n = 4) in the PO region (p = 0.6465). Scale bar: 2 s/100 μV.

**c**, Open field test showing similar travel distance (p = 0.7733), travel speed (p = 0.7329), mobile time (p = 0.7329) and immobile time (p = 0.6463) in NLG2 KO mice injected with CAG-NLG2-GFP (n = 5) compared to control CAG-GFP viruses (n = 5) in PO region of the thalamus. Scale bar: 1 mm.

Data represent mean ± SEM. ns = non-significant. Two-sided t -test was used. Source data are provided as a Source Data file.

**Supplementary Fig. 14**

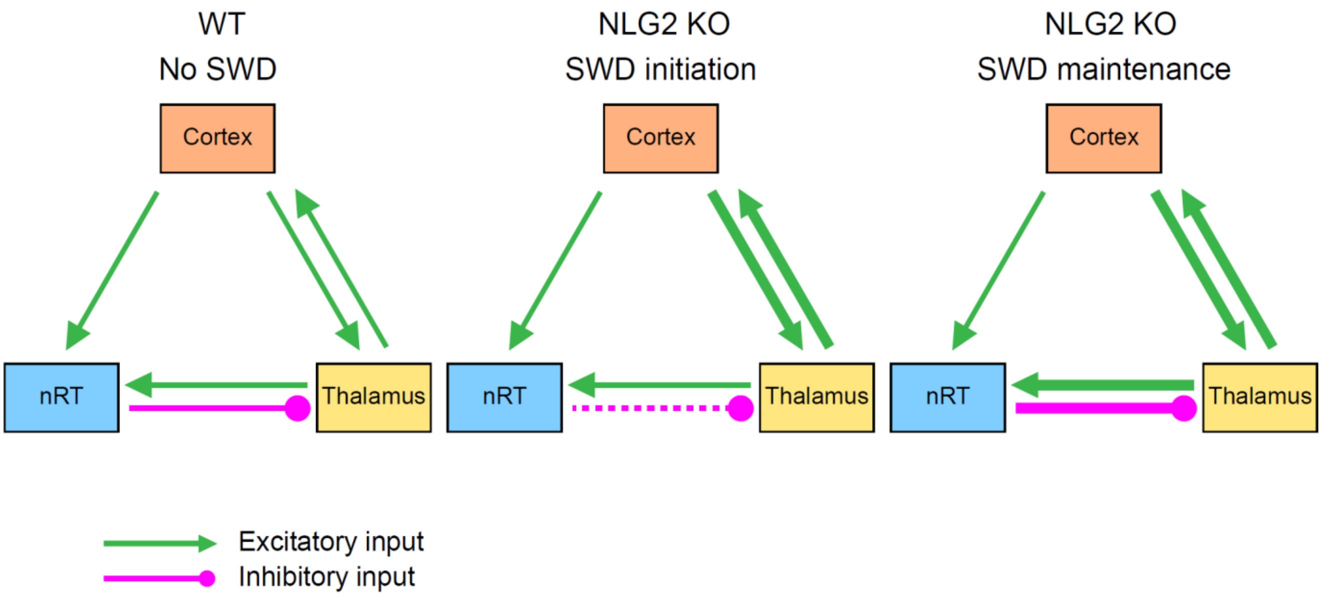


**Supplementary Fig. 14. Hypothetic model for SWD generation in NLG2 KO mice**

In NLG2 KO mice, the reduction in the fast feed-forward inhibition in thalamic neurons causes hyperexcitability of thalamic-cortical circuit due to reciprocal excitatory input between thalamic and cortical neurons, resulting in the initiation of SWDs. Hyperactive thalamic neurons subsequently over-activate nRT neurons, causing them to fire bursts of action potentials and inhibit thalamic neurons, which in turn fire bursts of action potentials from recovery from inhibition. This sequence of events leads to oscillation in the cortico-thalamo-cortical loop to maintain SWDs. The increased inhibition to thalamic neurons is possible because although fast inhibition mediated by synaptic $GABA_A$ receptors is reduced in thalamic neurons, there is no evidence that tonic inhibition mediated by extra-synaptic $GABA_A$ or $GABA_B$ receptors inhibition is weakened in NLG2 KO mice.

## Supplementary Table 1

| Passive and active electric membrane properties of cortical (CN), nRT and thalamic neurons (TN) in WT and NLG2 KO mice | | | | | | | |
|---|---|---|---|---|---|---|---|
| | Cm (pF) | Vm (mV) | Rm (mΩ) | AP threshold (mV) | AP amplitude (mV) | AP duration (ms) | cell # |
| WT-CN | 64.5±2.8 | -60.6±1.9 | 124.3±8.6 | -56.7±2.0 | 81.5±2.9 | 2.6±0.1 | 22 |
| KO-CN | 65.7±4.4 | -60.1±1.5 | 124.8±11.1 | -57.0±1.6 | 81.2±3.0 | 2.7±0.2 | 27 |
| p-value | ns | ns | ns | ns | ns | ns | |
| | | | | | | | |
| | Cm (pF) | Vm (mV) | Rm (mΩ) | AP threshold (mV) | AP amplitude (mV) | AP duration (ms) | cell # |
| WT-nRT | 66.2±4.1 | -55.3±3.0 | 114.8±7.9 | -48.5±1.7 | 79.2±3.5 | 2.6±0.1 | 12 |
| KO-nRT | 65.5±1.8 | -56.4±1.6 | 115.0±7.1 | -48.2±1.4 | 77.6±3.6 | 2.4±0.1 | 19 |
| p-value | ns | ns | ns | ns | ns | ns | |
| | | | | | | | |
| | Cm (pF) | Vm (mV) | Rm (mΩ) | AP threshold (mV) | AP amplitude (mV) | AP duration (ms) | cell # |
| WT-TN | 65.8±3.3 | -57.5±1.7 | 115.0±15.7 | -49.6±1.3 | 77.5±2.8 | 2.5±0.1 | 24 |
| KO-TN | 64.5±3.3 | -58.4±1.7 | 112.6±21.8 | -51.2±1.3 | 79.1±4.2 | 2.6±0.1 | 12 |
| p-value | ns | ns | ns | ns | ns | ns | |

**Supplementary Table 1. Passive and active electric membrane properties of the cortical (CN), nRT and thalamic (TN) neurons showing no differences between WT and NLG2 KO mice.**

The membrane capacity (Cm), resting membrane potential (Vm), membrane resistance (Rm), action potential (AP) threshold, amplitude and duration were similar in the CT, nRT and TC neurons between WT and NLG2 KO mice (two-sided t test, ns = non-significant, $p > 0.05$). Data represent mean ± SEM.
