# Supplementary material for: Neuroligin 2 regulates absence seizures and behavioral arrests through GABAergic transmission within the thalamocortical circuitry
Source: Nat Commun. 2020 Jul 27;11:3744. doi: 10.1038/s41467-020-17560-3 (PMC7385104; doi:10.1038/s41467-020-17560-3)
Supplement: Supplementary file 3 — Reporting Summary [file 41467_2020_17560_MOESM3_ESM.pdf]

nature research

Corresponding author(s): Zhengping Jia

Last updated by author(s): Jul 5, 2020

Reporting Summary

Nature Research wishes to improve the reproducibility of the work that we publish. This form provides structure for consistency and transparency in reporting. For further information on Nature Research policies, see Authors & Referees and the Editorial Policy Checklist.

Statistics

For all statistical analyses, confirm that the following items are present in the figure legend, table legend, main text, or Methods section.

n/a	Confirmed	
☐	☒	The exact sample size (*n*) for each experimental group/condition, given as a discrete number and unit of measurement
☐	☒	A statement on whether measurements were taken from distinct samples or whether the same sample was measured repeatedly
☐	☒	The statistical test(s) used AND whether they are one- or two-sided<br>*Only common tests should be described solely by name; describe more complex techniques in the Methods section.*
☐	☒	A description of all covariates tested
☐	☒	A description of any assumptions or corrections, such as tests of normality and adjustment for multiple comparisons
☐	☒	A full description of the statistical parameters including central tendency (e.g. means) or other basic estimates (e.g. regression coefficient) AND variation (e.g. standard deviation) or associated estimates of uncertainty (e.g. confidence intervals)
☐	☒	For null hypothesis testing, the test statistic (e.g. *F*, *t*, *r*) with confidence intervals, effect sizes, degrees of freedom and *P* value noted<br>*Give P values as exact values whenever suitable.*
☒	☐	For Bayesian analysis, information on the choice of priors and Markov chain Monte Carlo settings
☒	☐	For hierarchical and complex designs, identification of the appropriate level for tests and full reporting of outcomes
☐	☒	Estimates of effect sizes (e.g. Cohen's *d*, Pearson's *r*), indicating how they were calculated

Our web collection on statistics for biologists contains articles on many of the points above.

Software and code

Policy information about availability of computer code

Data collection	PClamp 10.6 (Clampex), GrassLab, ANYmaze 4.94h beta, NIS-Elements 5.11, LAS-X 3.4.1
Data analysis	Clampfit 11.0.3, GrassLab, Mini Analysis 6.0.7, ImageJ 1.52a (NIH), NIS-Element Viewer 4.5 , GraphPad PRISM 6 and 8, SigmaPlot 10 and 12, MatLab R2012a

For manuscripts utilizing custom algorithms or software that are central to the research but not yet described in published literature, software must be made available to editors/reviewers. We strongly encourage code deposition in a community repository (e.g. GitHub). See the Nature Research guidelines for submitting code & software for further information.

Data

Policy information about availability of data

All manuscripts must include a data availability statement. This statement should provide the following information, where applicable:

- Accession codes, unique identifiers, or web links for publicly available datasets
- A list of figures that have associated raw data
- A description of any restrictions on data availability

The authors declare that all relevant data in this study are included within the article and supplementary information. The source data underlying Figures 1e-h, 2a–h, 3c, f, g, j, k, 4a, b, d, e, g, h, j, k,5a-e, 6f-m, and 7d, h-l and Supplementary Figures 1a, c-f, 3b, c, 4c, e, f, 5c-f, 6b-d, 7a, b, 8a, b, 9a, b, 10a-e, 11a, b, and 13b, c are provided as a Source Data file. Source Data are provided with the paper. All requests for raw data and materials are promptly reviewed by the Hospital for Sick Children to verify whether the request is subject to any intellectual property or confidentiality obligations. Any materials that can be shared will be released via a Material Transfer Agreement.

Field-specific reporting

Please select the one below that is the best fit for your research. If you are not sure, read the appropriate sections before making your selection.

☒ Life sciences ☐ Behavioural & social sciences ☐ Ecological, evolutionary & environmental sciences

For a reference copy of the document with all sections, see nature.com/documents/nr-reporting-summary-flat.pdf

Life sciences study design

All studies must disclose on these points even when the disclosure is negative.

Sample size	The number of experiments were based on our previous publications on EEG recordings (Snead et al., J Neurosci. 2000, 20:6218-24), slice recordings, immunostaining and behavioral tests (Zhou et al.,Nat Neurosci. 2018, 21:50-62).
Data exclusions	No data exclusion except for those animals that died or disabled before the completion of the experiment.
Replication	Immunostaining and bright field sample images are representative of at least three individual experiments. Replication numbers in all other experiments are indicated in figure legends.
Randomization	All allocation was random. The experimenters did not know the genotype or treatment groups during the experiment.
Blinding	Investigators were blinded to the sample allocation, experimentation and data analysis

Reporting for specific materials, systems and methods

We require information from authors about some types of materials, experimental systems and methods used in many studies. Here, indicate whether each material, system or method listed is relevant to your study. If you are not sure if a list item applies to your research, read the appropriate section before selecting a response.

Materials & experimental systems

n/a	Involved in the study
☐	☒ Antibodies
☒	☐ Eukaryotic cell lines
☒	☐ Palaeontology
☐	☒ Animals and other organisms
☒	☐ Human research participants
☒	☐ Clinical data

Methods

n/a	Involved in the study
☒	☐ ChIP-seq
☒	☐ Flow cytometry
☒	☐ MRI-based neuroimaging

Antibodies

Antibodies used	Commercial antibodies were used in this study: anti-GFP (Aves Labs, Cat #GFP1010), anti-Neuroligin 2 (Synaptic System, Cat#129 203), anti-Parvalbumin (Sigma Aldrich, Cat#P3088, Clone PARV-19), anti-GFAP (Cell Signaling Technology, Cat#3670, Clone GA5, Lot Number 6).<br>The following secondary antibodies were used: donkey anti-chicken IgG, Alexa Fluor 488 (Jackson ImmunoResearch Laboratories, Cat#703-545-155), donkey anti-rabbit IgG, Alexa Fluor 568 (Invitroge, Cat#A10042), goat anti-mouse IgG, Alexa Fluor 488 (Invitrogen, Cat# A32723), and goat anti-mouse IgG, Alexa Fluor 546 (Invitroge, Cat# A-11030).
Validation	Validation information was based on the manufacturer's websites:<br>anti-GFP (Aves Labs, Cat #GFP1010) (https://www.aveslabs.com/collections/epitope-tag-6xhis-beta-gal-actin-and-gfp-antibodies/products/green-fluorescent-protein-gfp-antibody): The antibody was analyzed by western blot and immunohistochemistry using transgenic mice expressing the GFP gene.<br>anti-Neuroligin 2 (Synaptic System, Cat#129 203) (https://www.sysy.com/products/neuroligin2/facts-129203.php): The antibody reacts with human, rat, mouse, monkey, ape, cow. Specific for neuroligin 2, no cross reactivity to neuroligins 1, 3, 4. (K.O. verified), can be used for western blot, ELISA and immunostaining analysis.<br>anti-Parvalbumin (Sigma Aldrich, Cat#P3088, Clone PARV-19) (https://www.sigmaaldrich.com/catalog/product/sigma/p3088?lang=en&region=CA): The antibody reacts with a variety of species and used for immunohistochemistry in brain tissues.<br>anti-GFAP (Cell Signaling Technology, Cat#3670, Clone GA5, Lot Number: 6) (https://www.cellsignal.com/products/primary-antibodies/gfap-ga5-mouse-mab/3670?site-search-type=Products): The antibody detects endogenous levels of total GFAP protein from human, mouse and rat. Can be used for western blot, immunohistochemistry and immunofluorescence.

Animals and other organisms

Policy information about studies involving animals; ARRIVE guidelines recommended for reporting animal research

Laboratory animals	Neuroloigin (1,2,3, in the 129SvxC57BL/6 genetic background) KO mice, FMR1 KO mice and FVB mice were purchased from the Jackson Laboratory. PAK3 transgenic mice were made in house. The age of the mice ranged from 6-18 weeks with similar number of males and females. This information is indicated in the Methods of the paper.
Wild animals	No wild animals were used in the study.
Field-collected samples	No field collected samples were used in the study.
Ethics oversight	All animal protocols used in this study were approved by the Lab Animal Use Committee of the Hospital for Sick Children, Toronto, Canada

Note that full information on the approval of the study protocol must also be provided in the manuscript.
