# Supplementary material for: Neuroligin 2 regulates absence seizures and behavioral arrests through GABAergic transmission within the thalamocortical circuitry
Source: Nat Commun. 2020 Jul 27;11:3744. doi: 10.1038/s41467-020-17560-3 (PMC7385104; doi:10.1038/s41467-020-17560-3)
Supplement: Supplementary file 2 — Peer Review File [file 41467_2020_17560_MOESM2_ESM.docx]

Peer Review Information

**Journal:** Nature Communications

**Manuscript title:** Neuroligin 2 regulates absence seizures and behavioral arrests through GABAergic transmission within the thalamocortical circuitry

**Corresponding author name(s):** Dr Zhengping Jia

**Editorial notes:**

**none**

**Reviewer comments & decisions:**

**Reviewer comments, first version:**

Reviewer #1 (Remarks to the Author):

In the present study, Cao et al. investigate the consequences of deletion of the inhibitory synapse-specific adhesion protein Neuroligin 2 (NLG2) on neuronal activity in the thalamocortical circuitry. The authors report that NLG2 KO mice exhibit epileptiform spontaneous spike-wave discharges (SWDs) and behavioral arrests, which they interpret as absence seizures. They show that these seizures originate in the thalamus, and that they can be rescued by enhancing inhibitory synaptic transmission. Moreover, they show that deletion of NLG2 impairs inhibitory synaptic transmission in the thalamus but not somatosensory cortex or thalamic reticular nucleus (nRT). Finally, they report that enhancing synaptic transmission in the inhibitory connection from nRT -> thalamus or re-expressing NLG2 in thalamus is sufficient to partially normalize SWDs as well as several behavioral consequences of NLG2 deletion, indicating that this may represent a key mechanism in the function of NLG2.

Overall, this is a carefully conducted and detailed study on a topic of widespread interest to the neuroscience and molecular psychiatry community, and the manuscript is very well written. However, there are a number of issues that would need to be addressed in order to fully support the authors’ conclusions.

Specific comments:

1. In Figure 1a, the authors state that “Nlgn2 KO mice exhibited spontaneous spike-wave discharges (SWDs) that were associated with behavioral arrest episodes as indicated by reduced EMG signal”. However, there is no quantification of this effect, and the sample trace alone is not convincing, especially since it seems that that reduced EMG signal also occurs when there are no SWDs. Please provide statistical evidence that the SWDs are correlated with reduced EMG signal and with behavioral arrest. It would also be interesting to add the equivalent quantification for NLG1 and NLG3, e.g. in the supplement.

2. In Figures 2d and 2h, only a partial effect of local injection of ethosuximide (ETX) into thalamus is observed, whereas i.p. injections virtually abolish them. The authors claim that this is linked to the extent of drug diffusion (discussion line 297), but can they rule out that other brain regions also contribute to this effect? If not, this possibility should be discussed.

3. For the whole-cell patch clamp recordings reported in Figure 3 and Supplementary Figure 2, how did the authors specifically identify thalamocortical and corticothalamic neurons, respectively (lines 136-137)? Based on the schematic diagram and the methods description, it appears that they are patching all thalamic or all cortical neurons, not only those in the specified projections. If the specific projections were not labeled in any way, the sentence in line 136 should be modified to reflect that whole cell patch-clamp recordings were performed from cortical thalamic and nRT neurons.

4. The authors report seeing no change in mIPSC amplitude in somatosensory cortex of NLG2 KO mice (Supplementary Figure 2). How do they reconcile these findings with those from Gibson et al. 2009, showing that there is reduced mIPSC amplitude in somatosensory cortex of NLG2 KO mice (Figure 2)?

5. In Figure 4, NLG2 KO mice show normal social recognition memory in the five-trial social habituation test (Figure 4e,f, comparison trial 5 vs trial 6), but impaired social recognition memory in the three-chambered test (Figure 4h). What could be the reason for this discrepancy?

6. In Figure 4i-m, the authors show that i.p. administration of ETX ameliorates some of the behavioral abnormalities in NLG2 KO mice, and they conclude that thalamic SWDs are responsible for the impaired locomotor activity and anxiety behavior (lines 190 and 200). However, they do not show that blocking SWDs is the only effect of ETX, and in fact the behavioral consequences of ETX could be caused through other mechanisms. To strengthen the argument that the effects of ETX are caused by blocking SWDs, the authors should show the consequences of ETX in WT mice, which have no SWDs. By their logic, ETX should have no effect on the behavior of WT mice.

7. In addition, the effects of ETX on behavior in the open field test are minimal (Figure 4m) and do not reach anywhere near WT levels, indicating that the effect of ETX can only partially explain the anxiety and locomotor phenotype. The authors should therefore rephrase their conclusions to indicate that SWDs may be partially responsible for these phenotypes (lines 190, 200). The same holds true for the consequences of nRT -> thalamus activation through optogenetics (Figure 5i-l, line 232 – this should be rephrased accordingly).

8. In Figure 6c, the authors perform immunostaining for NLG2 to show that AAV-NLG2 is expressed. This immunostaining does not match the usual expression pattern of NLG2, which may be partly due to the fact that the authors use PFA-perfused sections rather than the post-fixation protocol which is usual for inhibitory postsynaptic proteins (see Schneider Gasser et al. 2006). The authors should confirm correct localization of AAV-NLG2 by performing co-staining with gephyrin or GABA(A)R antibodies, and they should include a negative control in which NLG2 is not expressed (e.g. GFP alone).

9. In Figure 6d, the authors claim that GFP does not colocalize with GFAP, but this does not match the representative image, in which there appear to be at least 5 GFP+ GFAP+ cells. Please provide quantification of the degree of colocalization of GFP with each marker.

Minor comments:

10. There are a number of relevant citations missing from the introduction, especially with respect to NLG2 function. In line 69, reduction of GABA receptors and GABA-mediated synaptic transmission has also been shown in Chubykin et al. 2007, Gibson et al. 2009, Liang et al. 2015, Babaev et al. 2016 etc. In line 72, genetic mutations in the NLG2 gene have also been associated with schizophrenia (Sun et al. 2011).

11. In the methods section, please include a description of the NLG1 and NLG3 KO lines in addition to the NLG2 KO line. Please also mention whether each of these lines were on a C57BL/6J or 6N background, or other. For the behavioral experiments, how many of the mice in each group were male and female?

12. In the statistics section, please confirm that the t-tests are two-tailed, or repeat statistical analysis to include two-tailed t-tests.

13. In Figure 1h, please scale the graph for the waking state such that the Y-axis begins at 0, to match the graphs for NREM and REM. In Figure 4j, please scale the graph for the closed arm time such that the Y-axis begins at 0, to match the other graphs.


Reviewer #2 (Remarks to the Author):

The manuscript by Cao et al. titled "Neuroligin 2 regulates absence seizures and behavioral arrests through GABAergic transmission within the thalamocortical circuitry' includes a series of experiments connecting thalamic GABAergic dysfunction with spike and wave discharges (SWDs) in the Neuroligin 2 knockout (KO) animal. The manuscript contains a variety of technical approaches, including slice electrophysiology, behavioral testing and optical manipulations. Overall, I thought the study was interesting, but some key claims are not supported by the presented data which need to be fixed. Also, the authors ought to be a bit more scholarly in acknowledging previous work that impinges on their study.

Major concerns:

1. Figures 1-3 make a good case for the authors to pursue the VB thalamus as a target for correcting SWDs in the NGL2 KO mouse. There are, however, a few issues with linking slice data to an EEG phoenomenon when a mesoscopic circuit description is lacking.

a. The recordings are said to be from the VB thalamus, but that is a big and heterogenous area. Are the cells from the VPM or PO? Does that have any bearing on the input/output function of these cells and are there systematic differences between WT and KO mice when circuit-specificity is considered?

b. What about bursting properties of these neurons? Are those affected in the KO?

c. What happens to the nRT in this animal? We only see evidence for changes in IPSCs (spontaneous and miniature), but it's unclear how much of it is related to changes in upstream inhibitory circuits (nRT or otherwise).

d. Does ethosuxamide normalize these measures in the slice?

2. I am not at all convinced by the connection between thalamic deficits, rescue and unifying ASD and SWD symptoms-- the main thesis that the authors are pushing. I think it's plausible, but not really shown here.

a. It is unclear to me why the authors think that ETX having an effect on their behavioral readouts suggest a link between SWDs and ASD symptoms. These can be completely independent effects exerted by ETX. Unless there is substantial work and some understanding of the underlying circuit dysfunction contributing to both pathological effects, how can one say that ETX is targeting a common mechanism? Could be that ETX has multiple independent effects. Unless the authors can rule that out, they must change their central thesis.

b. The NLG2 KO mice have a pretty specific phenotype in the three chamber social interaction, that may have something to do with social interactions or may not. How specific is this S1/S2 effects to social contexts? Maybe the animals simply have poorer memories because of SWDs and can't remember S1 vs. this being an ASD-related social deficit? I think much more work and thought needs to be put on this experiment and its link to thalamic deficits to see if there is something compelling here.

c. What are the parameters for the optogenetic 'rescue'? why do they work and what doesn't work? what can they tell us about therapeutically-viable strategies more generally?

d. Again, for NGL rescue in the last figure-- how much is this a VPM rescue vs. PO?

3. How general are NGL2 deficits in ASD? How applicable are the current findings (from a KO mouse) to ASD more generally?

4. Scholarly conduct: the authors do not cite previous work investigating the role of TRN in ASD, nor do the accompanying literature on TRN function in intact circuitry.


Reviewer #3 (Remarks to the Author):

This is a potentially very interesting report, suggesting that specific alterations in a single brain region, the thalamus, can result in epilepsy and social deficits in a mouse model of neuroligin 2 deficiency. This is striking, and unprecedented in many ways. Given that the results are unique and suggest a new model of epileptogenesis, the requirement for scientific rigor in supporting each new result is quite high. However, in several aspects the reported results do not meet this bar, so that despite the provocative conclusions, the paper is not ready for publication.

Specific points:

1) There is very little precedent for a *decrease* in synaptic inhibition in the thalamus causing absence seizures. The reduced frequency and amplitude of sIPSCs in VB thalamic neurons has not, to my knowledge, been reported before for an absence model. For example, see pubmed 16971676 and 19380748 which I believe show no change or an actual increase in gaba synapses. A theoretical model or at least framework to potentially explain these results should be provided.
2) Blockade of seizures by ethosuximide does not prove that the behavioral improves seen with ethosuximide were necessarily due to reduced seizures. This provocative and potentially very important result should be backed up by another method.
3) The mechanism through which both ko and overexpression of NLG2 produces seizures remains obscure.
4) The seizures themselves could be better characterized, as the illustrated examples are not as compelling as they might be. For example the relationship between seizures and EMG should be formally measured and reported. Further, the before and after control traces in figure 2a look distinct from each other.
5) In figure 3 it would be useful to show comparison of either cortical vs. VB or nRT vs VB to reflect differences in only VB.
6) Y tick labels in fig 4g and h are probably wrong.
7) Rescue of NLG seems to have relatively little effect, compared to KO. Effect sizes in 4m are small.
8) there are many controls missing from the optogenetic experiments. These include light only control (see pmid 31209378), virus with GFP/RFP but no ChR2. Effect sizes in fig 5f&g are quite modest. How can this explain the robust behavioral and seizure changes?
9) Controls for the overexpression results in figure 6 need to be delineated, and seem to be inadequate as is.
10) Previous studies, cited, seem to show that loss of NLGN2 reduces inhibition in cortex and thalamus. Some discussion is required to explain the results reported here that the defect in inhibition is restricted solely to VB neurons.
11) A justification for use of the CAG promoter for overexpression is required, vs. a neuron-specific promoter such as synapsin.
12) Methods do not describe the population of cortical neurons recorded.

**Author rebuttal, first version:**

**Reviewer #1 (Remarks to the Author):**

In the present study, Cao et al. investigate the consequences of deletion of the inhibitory synapse-specific adhesion protein Neuroligin 2 (NLG2) on neuronal activity in the thalamocortical circuitry. The authors report that NLG2 KO mice exhibit epileptiform spontaneous spike-wave discharges (SWDs) and behavioral arrests, which they interpret as absence seizures. They show that these seizures originate in the thalamus, and that they can be rescued by enhancing inhibitory synaptic transmission. Moreover, they show that deletion of NLG2 impairs inhibitory synaptic transmission in the thalamus but not somatosensory cortex or thalamic reticular nucleus (nRT). Finally, they report that enhancing synaptic transmission in the inhibitory connection from nRT -> thalamus or re-expressing NLG2 in thalamus is sufficient to partially normalize SWDs as well as several behavioral consequences of NLG2 deletion, indicating that this may represent a key mechanism in the function of NLG2.

Overall, this is a carefully conducted and detailed study on a topic of widespread interest to the neuroscience and molecular psychiatry community, and the manuscript is very well written. However, there are a number of issues that would need to be addressed in order to fully support the authors’ conclusions.

**Response:** Thank you very much for your positive assessment of the paper. We appreciate your suggestions and comments, which we have addressed as follows.

Specific comments:

1. In Figure 1a, the authors state that “Nlgn2 KO mice exhibited spontaneous spike-wave discharges (SWDs) that were associated with behavioral arrest episodes as indicated by reduced EMG signal”. However, there is no quantification of this effect, and the sample trace alone is not convincing, especially since it seems that that reduced EMG signal also occurs when there are no SWDs. Please provide statistical evidence that the SWDs are correlated with reduced EMG signal and with behavioral arrest. It would also be interesting to add the equivalent quantification for NLG1 and NLG3, e.g. in the supplement.

**Response:** As suggested, we have quantified the amount of behavior freezing during SWDs and analyzed the correlation between EEG and EMG amplitudes. The data are presented in Supplementary Fig. 1. The data indicate that the SWDs are tightly (99.038% of the SWDs) associated with behavioral arrests (lack of whole-body movement) in NLG2 KO mice and that there is a negative correlation between the EEG and EMG amplitudes in NLG2 KO mice, but not in NLG1 or 3 KO mice.

1

2. In Figures 2d and 2h, only a partial effect of local injection of ethosuximide (ETX) into thalamus is observed, whereas i.p. injections virtually abolish them. The authors claim that this is linked to the extent of drug diffusion (discussion line 297), but can they rule out that other brain regions also contribute to this effect? If not, this possibility should be discussed.

**Response:** Yes, we agree with the reviewer that we cannot rule out the possibility that other brain regions may also involved. We have discussed this possibility in the Discussion (page 15).

3. For the whole-cell patch clamp recordings reported in Figure 3 and Supplementary Figure 2, how did the authors specifically identify thalamocortical and corticothalamic neurons, respectively (lines 136-137)? Based on the schematic diagram and the methods description, it appears that they are patching all thalamic or all cortical neurons, not only those in the specified projections. If the specific projections were not labeled in any way, the sentence in line 136 should be modified to reflect that whole cell patch-clamp recordings were performed from cortical thalamic and nRT neurons.

**Response:** Yes, we agree with the reviewer that because the specific projections were not labeled, whole cell patch-clamp recordings were from all cortical (layer 5/6), thalamic (VPL/VPM), and nRT neurons. We have changed thalamocortical and corticothalamic neurons into thalamic and cortical neurons respectively throughout the manuscript.

4. The authors report seeing no change in mIPSC amplitude in somatosensory cortex of NLG2 KO mice (Supplementary Figure 2). How do they reconcile these findings with those from Gibson et al. 2009, showing that there is reduced mIPSC amplitude in somatosensory cortex of NLG2 KO mice (Figure 2)?

**Response:** Yes, we are aware of this discrepancy and this is likely due to the fact that the NLG2 deletion only affects certain inhibitory synapses. In the Gibson et al study (2009), it was found that mIPSCs from the parvalbumin positive (PV+) projection, but not from somatostatin positive (SST+) projection, were reduced. Because the PV+ neurons only account for a portion (40%) of all interneurons in the cortex (Rudy et al., 2011; Tremblay et al., 2016) and that we recorded mIPSCs from all inhibitory projections to layer 5/6 neurons, it is not surprising that no significant differences were detected between WT and NLG2 KO mice in our studies. Our results indicate that although inhibitory transmission is reduced at the PV+ input to the somatosensory cortical neurons in NLG2 KO mice, their overall inhibition is not be altered, possibly due to the presence of other inhibitory inputs (e.g., SST+). We have added this to the Discussion (page 16).

5. In Figure 4, NLG2 KO mice show normal social recognition memory in the five-trial social habituation test (Figure 4e,f, comparison trial 5 vs trial 6), but impaired social recognition memory in the three-chambered test (Figure 4h). What could be the reason for this discrepancy?

2

**Response:** Although these two tests both assess social interaction and recognition memory, they have different sensitivity and caveats. For example, the five-trial social test undergoes multiple, consecutive trials that may lead to habituation/fatigue, and therefore, it could be less sensitive to detect some impairments in the KO mice, as compared to the three-chamber test.

6. In Figure 4i-m, the authors show that i.p. administration of ETX ameliorates some of the behavioral abnormalities in NLG2 KO mice, and they conclude that thalamic SWDs are responsible for the impaired locomotor activity and anxiety behavior (lines 190 and 200). However, they do not show that blocking SWDs is the only effect of ETX, and in fact the behavioral consequences of ETX could be caused through other mechanisms. To strengthen the argument that the effects of ETX are caused by blocking SWDs, the authors should show the consequences of ETX in WT mice, which have no SWDs. By their logic, ETX should have no effect on the behavior of WT mice.

**Response:** We agree with this reviewer and therefore have performed additional experiments to strengthen our argument. First, as suggested, we performed ETX experiments in WT mice and shown that it did not have any affect on locomotor activity or social recognition memory (Supplementary Fig. 6, Fig. 5c, d). Second, we have performed ETX experiments in another mouse model (PAK3 transgenic mice) that show social memory deficits but without SWD (Leung et al., 2018), and again ETX had no effects on social memory deficits in this mouse model (Supplementary Fig. 7). Finally, we tested the effect of ETX in the FMR1 KO mice, a widely used mouse model for syndromic ASD that show social recognition memory deficits (Kazdoba et al., 2014). The FMR1 KO mice also have increased susceptibility to seizures but different from absence seizures (Berry-Kravis, 2002). As shown in Supplementary Fig. 8, ETX also did not improve the social memory impairments in these mice. These results, together with the fact that ETX’s therapeutic effect is highly specific to absence seizures, strengthen the idea that ETX affects locomotor activity and social memory via reducing SWDs in NLG2 KO mice. However, we cannot rule out the possibility that ETX may improves these behaviors independently of SWDs and we have raised this point in the Discussion (page 18, 19).

7. In addition, the effects of ETX on behavior in the open field test are minimal (Figure 4m) and do not reach anywhere near WT levels, indicating that the effect of ETX can only partially explain the anxiety and locomotor phenotype. The authors should therefore rephrase their conclusions to indicate that SWDs may be partially responsible for these phenotypes (lines 190, 200). The same holds true for the consequences of nRT -> thalamus activation through optogenetics (Figure 5i-l, line 232 – this should be rephrased accordingly).

**Response:** The IP injections of ETX actually had a robust effect on the behavioral deficits in NLG2 KO mice (Fig. 4a, b, g, h and Fig. 5a-d). However, we do agree with the reviewer that local injections of ETX in the thalamus only partially rescued the locomotor phenotype (Fig. 5e). Similarly, the optogenetic activation of the nRT-thalamic pathway or overexpression of WT NLG2 in the thalamic neurons also partially rescued the motor deficits in NLG2 KO mice (Fig. 6h, Fig. 7j). Although the partial recue in these experiments could be due to the extent of drug diffusion in the local drug injection experiment or the fact that only a fraction of nRT or thalamic

3

neurons are virally infected, resulting in incomplete blockade of the SWDs, we agree with the reviewer that there is a possibility that ETX may act through other mechanisms independent of the SWDs and/or that other brain regions also contribute to these behavioral deficits in NLG2 KO mice. We have raised this possibility in the Discussion (page 18, 19). We have also rephrased the text to indicate that SWDs or nRT-thalamic pathway may be partially responsible for the locomotor and anxiety phenotype in the KO mice.

8. In Figure 6c, the authors perform immunostaining for NLG2 to show that AAV-NLG2 is expressed. This immunostaining does not match the usual expression pattern of NLG2, which may be partly due to the fact that the authors use PFA-perfused sections rather than the post-fixation protocol which is usual for inhibitory postsynaptic proteins (see Schneider Gasser et al. 2006). The authors should confirm correct localization of AAV-NLG2 by performing co-staining with gephyrin or GABA(A)R antibodies, and they should include a negative control in which NLG2 is not expressed (e.g. GFP alone).

**Response:** As suggested we have performed immunostaining experiments using the protocol described in Schneider Gasser et al. 2006, but unfortunately in our hands, the result was not as clear as that using the PFA-perfused sections. We have also tried commercially available GABA(A)R and gephyrin antibodies but without success. Thus, we have performed additional experiments using the PFA-perfused sections. As shown in Fig. 7e and f, NLG2 is localized mostly on the cell surface of GFP positive neuronal cell bodies. In the GFP control infected NLG2 KO mice, no significant immunostaining signals for NLG2 were detected.

9. In Figure 6d, the authors claim that GFP does not colocalize with GFAP, but this does not match the representative image, in which there appear to be at least 5 GFP+ GFAP+ cells. Please provide quantification of the degree of colocalization of GFP with each marker.

**Response:** We are sorry for the confusion. The image in original Fig. 6d (now Fig. 7c in the revised paper) was obtained from the infected cortex because we rarely detected any GFAP+ cells in the thalamus. We present the cortical images to simply indicate the extent of viral infections in both neurons and astrocytes. We have now quantified the infection rate and the results are shown in Fig. 7d. In the cortex, 93.7% GFP+ cells are Neurotrace+ and 2.3% GFP+ cells are GFAP positive. We also counted the colocalization of GFP+ neuron and Neurotrace in the infected thalamus and found that 98.3% GFP+ cells are Neurotrace+. These results indicated that the majority of CAG-NLG2-GFP virus infected cells are neurons. We have now also repeated the rescue experiments with the CamKII-NLG2-GFP virus and the results are similar (Fig. 7k, l).

Minor comments:

10. There are a number of relevant citations missing from the introduction, especially with respect to NLG2 function. In line 69, reduction of GABA receptors and GABA-mediated synaptic transmission has also been shown in Chubykin et al. 2007, Gibson et al. 2009, Liang et

*4*

al. 2015, Babaev et al. 2016 etc. In line 72, genetic mutations in the NLG2 gene have also been associated with schizophrenia (Sun et al. 2011).

**Response:** Thank you very much for pointing out this oversight and we have now inserted these references.

11. In the methods section, please include a description of the NLG1 and NLG3 KO lines in addition to the NLG2 KO line. Please also mention whether each of these lines were on a C57BL/6J or 6N background, or other. For the behavioral experiments, how many of the mice in each group were male and female?

**Response:** We have added relevant information in the Methods section. The genetic background of these NLG KO mice is C57BL/6Jx129sv. For behavior tests, roughly half of each group were male or female, but the exact number was not presented. All this information has been included in the Methods section

12. In the statistics section, please confirm that the t-tests are two-tailed, or repeat statistical analysis to include two-tailed t-tests.

**Response:** Yes, the two tailed t-tests were used for comparing two groups and one- or two-way ANOVA were used for more than two groups followed by Post-hoc Holm-Sidak’s multiple comparisons

13. In Figure 1h, please scale the graph for the waking state such that the Y-axis begins at 0, to match the graphs for NREM and REM. In Figure 4j, please scale the graph for the closed arm time such that the Y-axis begins at 0, to match the other graphs.

**Response:** As suggested, we have made these changes**.**

**Reviewer #2 (Remarks to the Author):**

The manuscript by Cao et al. titled "Neuroligin 2 regulates absence seizures and behavioral arrests through GABAergic transmission within the thalamocortical circuitry' includes a series of experiments connecting thalamic GABAergic dysfunction with spike and wave discharges (SWDs) in the Neuroligin 2 knockout (KO) animal. The manuscript contains a variety of technical approaches, including slice electrophysiology, behavioral testing and optical manipulations. Overall, I thought the study was interesting, but some key claims are not supported by the presented data which need to be fixed. Also, the authors ought to be a bit more scholarly in acknowledging previous work that impinges on their study.

Major concerns:

1. Figures 1-3 make a good case for the authors to pursue the VB thalamus as a target for correcting SWDs in the NGL2 KO mouse. There are, however, a few issues with linking slice

5

data to an EEG phenomenon when a mesoscopic circuit description is lacking.

a. The recordings are said to be from the VB thalamus, but that is a big and heterogenous area. Are the cells from the VPM or PO? Does that have any bearing on the input/output function of these cells and are there systematic differences between WT and KO mice when circuit-specificity is considered?

**Response:** Based on the placement of our recording electrode in the slices (Fig. 3a), most of our thalamic recordings would be from the VPL/VPM neurons. However, we do not know from which projections these synaptic responses were generated, because we did not label specific inputs. Because the nRT neurons project to both the VP and PO neurons, it is possible that both neurons are impaired in inhibitory responses in NLG2 KO mice, and in this case, we expect systematic differences in the nRT-thalamic projections between WT and KO mice. It would be interesting in future studies to determine whether all or specific inhibitory pathways to the thalamic neurons are altered in NLG2 KO mice.

b. What about bursting properties of these neurons? Are those affected in the KO?

**Response:** We have now analyzed the bursting properties of the nRT and thalamic neurons, elicited by hyperpolarizing current injections and the results showed that they were not altered in NLG2 KO mice (Supplementary Fig. 9a, b).

c. What happens to the nRT in this animal? We only see evidence for changes in IPSCs (spontaneous and miniature), but it's unclear how much of it is related to changes in upstream inhibitory circuits (nRT or otherwise).

**Response:** Yes, we also recorded IPSCs, EPSCs and firing properties from the nRT (Fig. 3, Supplementary Fig. 4 and 9a) and somatosensory cortical layer 5/6 neurons (Supplementary Fig. 5) and found no differences between WT and NLG2 KO mice.

d. Does ethosuxamide normalize these measures in the slice?

**Response:** We have now performed additional experiments and shown that ETX treatment (500 uM) did not affect the firing frequency induced by depolarizing current injections (Supplementary Fig. 9d) or sIPSCs in WT or NLG2 KO slices. However, ETX reduced the action potential number in the rebound burst firing in both genotypes (Supplementary Fig. 9b and c). These results suggest that ETX blocks the SWDs in NLG2 KO mice likely through reducing the rebound burst firing upon hyperpolarization, but not through affecting IPSCs.

2. I am not at all convinced by the connection between thalamic deficits, rescue and unifying ASD and SWD symptoms-- the main thesis that the authors are pushing. I think it's plausible, but not really shown here.

a. It is unclear to me why the authors think that ETX having an effect on their behavioral readouts suggest a link between SWDs and ASD symptoms. These can be completely independent effects exerted by ETX. Unless there is substantial work and some understanding of

6

the underlying circuit dysfunction contributing to both pathological effects, how can one say that ETX is targeting a common mechanism? Could be that ETX has multiple independent effects. Unless the authors can rule that out, they must change their central thesis.

**Response:** Yes, we agree with this reviewer that ETX could affect SWDs and ASD

independently. However, our additional data suggest that these effects are linked to each other. . First, we performed ETX experiments in WT mice and shown that it did not have any affect on locomotor activity or social recognition memory (Supplementary Fig. 6, Fig. 5c, d). Second, we have performed ETX experiments in another mouse model (PAK3 transgenic mice) that show social memory deficits but without SWDs (Leung et al., 2018), and again ETX had no effects on social memory deficits in this mouse mode (Supplementary Fig. 7). Finally, we tested the effect of ETX in the FMR1 KO mice, a widely used mouse model for syndromic ASD that show social recognition memory deficits (Kazdoba et al., 2014). The FMR1 KO mice also have increased susceptibility to seizures but different from absence seizures (Berry-Kravis, 2002). As shown in Supplementary Fig. 8, ETX also did not improve the social memory impairments in these mice. These results, together with the fact that ETX’s therapeutic effect is highly specific to absence seizures, strengthen the idea that ETX affects locomotor activity and social memory via reducing SWDs in NLG2 KO mice. However, we cannot rule out the possibility that ETX may improves these behaviors independently of SWDs and we have raised this point in the Discussion (page 18, 19).

b. The NLG2 KO mice have a pretty specific phenotype in the three-chamber social interaction, that may have something to do with social interactions or may not. How specific is this S1/S2 effects to social contexts? Maybe the animals simply have poorer memories because of SWDs and can't remember S1 vs. this being an ASD-related social deficit? I think much more work and thought needs to be put on this experiment and its link to thalamic deficits to see if there is something compelling here.

**Response:** To determine whether NLG2 KO mice are impaired in general learning and memory we have now performed the novel objection recognition test and shown that there is no difference between WT and NLG2 KO mice (Fig. 4i-k). These results suggest that the KO mice can recognize novelty and form memory, in a similar temporal and spatial setting. Therefore, the memory defect in NLG2 KO mice is specific to social recognition memory.

c. What are the parameters for the optogenetic 'rescue'? why do they work and what doesn't work? what can they tell us about therapeutically-viable strategies more generally?

**Response:** We only presented the data using a protocol where optical stimulation with 5 s epoch continuous light on (473 nm, 25-35 mW) and 5 s epoch light off were alternately applied for 5 min in freely moving mice during EEG recordings or behavior tests. Other protocols (e.g., alternated 10 s epoch light off and 10 s epoch light on at 5 Hz or 10 Hz for 5 min) were also tested but shown to have no effects on the SWDs or locomotor activity, therefore they were not further investigated. The reason for the latter protocols not being effective is not known, but could be related to the intrinsic neuronal activity of the nRT neurons. The fact that 5 s epoch of

7

continuous blue-light stimulation is effective suggesting that tonic activation of the nRT neurons may be used as a therapeutic strategy to disrupt the SWDs.

d. Again, for NGL rescue in the last figure-- how much is this a VPM rescue vs. PO?

**Response:** Due to the lack of VPM and PO specific immunomarkers, we cannot be certain about the relative ratio of NLG2 expression in these neurons, but based on the expression pattern of GFP in our recorded slices, the neurons that were infected were VPL/VPM neurons. .

3. How general are NGL2 deficits in ASD? How applicable are the current findings (from a KO mouse) to ASD more generally?

**Response:** Most of gene mutations associated with ASD, including those found in the NLG2 gene, are rare mutations. However, because the NLG2 affects excitation/inhibition balance, a common mechanism targeted by many other ASD-causing gene mutations, the finding in the present study that deficits at the nRT projection to the thalamic neurons could also be applicable to other gene mutations**.**

4. Scholarly conduct: the authors do not cite previous work investigating the role of TRN in ASD, nor do the accompanying literature on TRN function in intact circuitry.

**Response:** Sorry for this oversight. We have now included description on the roles of nRT/TC in normal physiology (including slow wave sleep and sleep spindle) and in ASD in the Discussion (page 14, 15).

Reviewer #3 (Remarks to the Author):

This is a potentially very interesting report, suggesting that specific alterations in a single brain region, the thalamus, can result in epilepsy and social deficits in a mouse model of neuroligin 2 deficiency. This is striking, and unprecedented in many ways. Given that the results are unique and suggest a new model of epileptogenesis, the requirement for scientific rigor in supporting each new result is quite high. However, in several aspects the reported results do not meet this bar, so that despite the provocative conclusions, the paper is not ready for publication.

**Response:** We appreciate this reviewer’s comments that our results are unique and suggest a new model of absence seizures. As suggested, we have performed additional experiments to address the concerns raised by this reviewer, which are detailed below in response to specific points.

Specific points:

1) There is very little precedent for a *decrease* in synaptic inhibition in the thalamus causing absence seizures. The reduced frequency and amplitude of sIPSCs in VB thalamic neurons has not, to my knowledge, been reported before for an absence model. For example, see pubmed

8

16971676 and 19380748 which I believe show no change or an actual increase in gaba synapses. A theoretical model or at least framework to potentially explain these results should be provided.

**Response:** Yes, we agree that despite the fact that mutations in specific subunits GABAA receptors ( e.g., α1 subunit, Arain et al., 2012; γ2 subunit, Frugier et al., 2007; Tan et al., 2007)) have been found in human absence epilepsy, and that mice haploinsufficient for the GABAA receptor α1 subunit, recapitulated the human absence phenotype (Arain et al., 2012), the changes in synaptic inhibition in these animal models are highly region specific and a decrease in GABA transmission was only found the nRT or cortical neurons, but not in the thalamic neurons.

However, our results are in general consistent with a model of SWDs generated by a feedforward disinhibition (Maheshwari and Noebels, 2014) mediated by reduced GABAA receptors on the thalamic neurons (Supplementary Fig. 12). In this model, and given the role of cortical neurons in the early stage of SWDs (Meeren et al., 2014; Polack et al., 2007), reduced fast synaptic inhibition in the thalamic neurons causes thalamic and cortical hyperexcitability, leading to the initiation of absence oscillation. Hyperactive thalamic neurons activate nRT cells which in turn recruit tonic inhibition via activation of GABAB receptors or extra-synaptic GABAA receptors on the thalamic neurons therefore allowing for the maintenance of SWDs and absence seizures Also see the Discussion (page 17).

This model of SWDs is in principle similar to that observed in GluA4 KO mice,

where synaptic transmission is impaired only at the cortico-nRT excitatory synapse, resulting in reduced feedforward inhibition of the thalamus (Paz et al., 2011). Another similar example of reduced inhibition resulting in absence seizures is PICK1 KO mice (Bertaso et al., 2008). PICK1 interacts with a metabotropic glutamate receptor 7 that is localized presynaptically at the nRT-thalamic synapses to prevent thalamic hyperexcitability (Kyuyoung and Huguenard, 2014). PICK1 loss-of-function mutations lead to thalamic hyperexcitability that can then recruit the circuit into SWDs.

Compared to these two models, the NLG2 KO mouse model is unique in that SWDs are caused by reduced postsynaptic inhibitory responses, rather than the impaired excitability or neurotransmitter release of the nRT neurons.

2) Blockade of seizures by ethosuximide does not prove that the behavioral improves seen with ethosuximide were necessarily due to reduced seizures. This provocative and potentially very important result should be backed up by another method.

**Response:** Yes, we agree with this reviewer that ETX could affect SWDs and ASD independently. However, the results of our additional experiments strengthen our conclusion. First, we performed ETX experiments in WT mice and shown that it did not have any affect on locomotor activity or social recognition memory (Supplementary Fig. 6, Fig. 5c, d). Second, we have performed ETX experiments in another mouse model (PAK3 transgenic mice) that show social memory deficits but without SWDs (Leung et al., 2018), and again ETX had no effects on social memory deficits in this mouse mode (Supplementary Fig. 7). Finally, we tested the effect of ETX in the FMR1 KO mice, a widely used mouse model for syndromic ASD that show social recognition memory deficits (Kazdoba et al., 2014). The FMR1 KO mice also have increased

9

susceptibility to seizures but different from absence seizures (Berry-Kravis, 2002). As shown in Supplementary Fig. 8, ETX also did not improve the social memory impairments in these mice. These results, together with the fact that ETX’s therapeutic effect is highly specific to absence seizures, strengthen the idea that ETX affects locomotor activity and social memory via reducing SWDs in NLG2 KO mice. Our optogenetic and NLG2 overexpression experiments, where the disruption of SWDs is also linked to the behavioral rescue, also support that SWDs were an underlying mechanism for behavior arrests and social memory deficits. However, we cannot completely rule out the possibility that ETX may improves these behaviors independently of SWDs and we have raised this point in the Discussion (page 18, 19).

3) The mechanism through which both ko and overexpression of NLG2 produces seizures remains obscure.

**Response:** Yes, we agree with this reviewer that although NLG2 KO and overexpression both lead to seizures, the underlying mechanisms remain unknown and more experiments are needed. First, it is important to know whether the seizures in the NLG2 overexpressing mice are the same as in the KO mice. Then it is important to delineate the brain regions that are affected in these mice. It is possible that NLG2 overexpression causes increased inhibition specifically in the thalamic neurons that promote burst firing and SWDs. We have discussed this in the Discussion (page 17).

4) The seizures themselves could be better characterized, as the illustrated examples are not as compelling as they might be. For example, the relationship between seizures and EMG should be formally measured and reported. Further, the before and after control traces in figure 2a look distinct from each other.

**Response:** As suggested we have analyzed the amplitudes of EEG and EMG and shown that they are negatively corelated (Supplementary Fig. 1b-d). In addition, we also provided quantification showing that behavior freezing is closely associated with SWDs (Supplementary Fig. 1a). The before and after control traces in Fig. 2a have also been corrected.

5) In figure 3 it would be useful to show comparison of either cortical vs. VB or nRT vs VB to reflect differences in only VB.

**Response:** As suggested, we have moved some of the nRT data from the original supplementary figure to Fig 3e-k for direct comparisons between the thalamic and nRT neurons, which show that the reduction in IPSCs only occurs in the thalamic neurons.

6) Y tick labels in fig 4g and h are probably wrong.

**Response:** Thanks for pointing out these errors. These have been corrected.

7) Rescue of NLG seems to have relatively little effect, compared to KO. Effect sizes in 4m are small.

10

**Response:** The effect of NLG2 expression on IPSCs is robust as both the frequency and amplitude of infected NLG2 KO neurons are similar to those of WT neurons (Fig. 7h). However, the SWDs and behavior rescue, although significant, is only partial compared to WT mice (Fig. 7i, j). This is most likely due to the fact that the virus only infected some, but not all, thalamic neurons and therefore only a fraction of the thalamic neurons restore their normal function. It is also possible that the SWDs and nRT-thalamic circuit are partially responsible for the motor deficits and that other brain regions may also be involved. We have raised these possibilities in the Discussion (page 18, 19).

The sample size of Fig. 4m (now Fig. 5e in the revised paper) has been increased to 10.

8) there are many controls missing from the optogenetic experiments. These include light only control (see pmid 31209378), virus with GFP/RFP but no ChR2. Effect sizes in fig 5f&g are quite modest. How can this explain the robust behavioral and seizure changes?

**Response:** As suggested, we have now included the following control experiments:

(1). Light control only (with only optical fibers but no virus injections), which had no effects on sIPSCs or locomotor activity in the open field test in WT mice (Supplementary Fig. 10).
(2). Control virus with GFP but no ChR2, which also had no effects on sIPSCs, SWDs or locomotor activity in either WT or NLG2 KO mice (Fig. 6j-m).

We agree with the reviewer that optical activation of the nRT-thalamic terminals had small effects on sIPSCs (now Fig. 6f & g in the revised paper), but relatively larger effects on the SWDs. This suggests that small changes in GABAergic transmission at the nRT-thalamic synapse could have larger effects on the cortical-thalamic circuit level. But it is important to note that the optical activation also had small effects on locomotor activity (Fig. 6i).

9) Controls for the overexpression results in figure 6 need to be delineated, and seem to be inadequate as is.

**Response:** As suggested, we have added more control experiments

(1). Control AAV-CAG-GFP in immunostaining experiments showing no expression of NLG2 as compared to AAV-CAG-NLG2-GFP in NLG2 KO mice (Fig. 7e).
(2). Control AAV-CAG-GFP in slice recordings showing no effects on sIPSCs as compared to AAV-CAG-NLG2-GFP in NLG2 KO mice (Fig. 7g and h).
(3). Control AAV-CAG-GFP in EEG recordings showing no effects on the SWDs as compared to AAV-CAG-NLG2-GFP in NLG2 KO mice (Fig. 7i).
(4). Control AAV-CAG-GFP in open field test showing no effects on locomotor activity as compared to AAV-CAG-NLG2-GFP in NLG2 KO mice (Fig. 7j).

11

(5). We also repeated rescue experiments with two additional viruses: AAV-CamKII-NLG2-GFP and its control AAV-CamKII-GFP (where the expression of NLG2-GFP is under the control of the excitatory neuronal promoter CamKIIα) and showed that AAV-CamKII-NLG2-GFP significantly increased sIPSCs and locomotor activity in NLG2 KO mice (Fig. 7k, l).

10) Previous studies, cited, seem to show that loss of NLGN2 reduces inhibition in cortex and thalamus. Some discussion is required to explain the results reported here that the defect in inhibition is restricted solely to VB neurons.

**Response:** Yes, we are aware of the discrepancy and believe that this is likely due to the fact that the NLG2 deletion only affects certain inhibitory synapses. In the Gibson et al (2009) studies, it was found that mIPSCs from the parvalbumin positive (PV+) projection, but not from somatostatin positive (SST+) projection, were reduced. Because the PV+ neurons only account for a portion (40%) of all interneurons in the cortex (Rudy et al., 2011; Tremblay et al., 2016) and that we recorded mIPSCs from all inhibitory projections to layer 5/6 neurons, it is not surprising that no significant differences were detected between WT and NLG2 KO mice in our studies. Our results indicate that although inhibitory transmission is reduced at the PV+ input to the somatosensory cortical neurons in NLG2 KO mice, their overall inhibition is not be altered, possibly due to the presence of other inhibitory inputs (e.g., SST+). We have added this to the Discussion (page 16).

11) A justification for use of the CAG promoter for overexpression is required, vs. a neuron-specific promoter such as synapsin.

**Response:** The reason to initially choose the CAG promoter was that the virus was prepared more easily using this promoter. Based on our analysis, most of infected cells are neurons in both cortex and thalamus (Fig. 7a-f). Recently we have also obtained AAV viruses containing NLG2 under the control of the excitatory neuronal promoter CamKIIα (AAV-CamKII-NLG2-GFP and its control AAV-CamKII-GFP) and shown that viral expression of CaMKIIα-NLG2-GFP in the thalamic neurons also significantly increased IPSCs and locomotor activity in NLG2 KO mice (Fig. 7k, l).

12) Methods do not describe the population of cortical neurons recorded.

**Response:** We have now included a description of cortical neurons (Layer 5/6) that we recorded in the Methods.

**References:**

Arain, F.M., Boyd, K.L., Gallagher, M.J., 2012a. Decreased viability and absence-like epilepsy

in mice lacking or deficient in the GABAA receptor a1 subunit. Epilepsia

53, e161–e165.

12

Babaev O, Botta P, Meyer E, Müller C, Ehrenreich H, Brose N, Lüthi A, Krueger-Burg D. (2016) [Neuroligin 2 deletion alters inhibitory synapse function and anxiety-associated neuronal activation in the amygdala.](https://www.ncbi.nlm.nih.gov/pubmed/26142252) Neuropharmacology. 2016 Jan;100:56-65.

Bertaso, F., Zhang, C., Scheschonka, A., de Bock, F., Fontanaud, P., Marin, P., Huganir, R.L., Betz, H., Bockaert, J., Fagni, L., Lerner-Natoli, M., 2008. PICK1 uncoupling from mGluR7a causes absence-like seizures Nat. Neuroscience 11, 940–948. [http://dx.doi. org/10.1038/nn.2142.](http://dx.doi.org/10.1038/nn.2142.)

Berkovic, S.F., Sprengel, R., Petrou, S., 2007. Reduced cortical inhibition in a mouse

model of familial childhood absence epilepsy. Proc. Natl. Acad. Sci. U.S.A.

104, 17536–17541.

Berry-Kravis E. (2002). [Epilepsy in fragile X syndrome.](https://www.ncbi.nlm.nih.gov/pubmed/12418611) Dev Med Child Neurol. 44(11):724-8.

Bessaïh T, Bourgeais L, Badiu CI, Carter DA, Toth TI, Ruano D, Lambolez B, Crunelli V, Leresche N. (2006). [Nucleus-specific abnormalities of GABAergic synaptic transmission in a genetic model of absence seizures.](https://www.ncbi.nlm.nih.gov/pubmed/16971676) J Neurophysiol. 2006 Dec;96(6):3074-81

Chubykin AA, Atasoy D, Etherton MR, Brose N, Kavalali ET, Gibson JR, Südhof TC. (2007). [Activity-dependent validation of excitatory versus inhibitory synapses by neuroligin-1 versus neuroligin-2.](https://www.ncbi.nlm.nih.gov/pubmed/17582332) Neuron. 2007 Jun 21;54(6):919-31.

Crunelli V, Cope DW, Terry JR. (2011). [Transition to absence seizures and the role of GABA(A) receptors.](https://www.ncbi.nlm.nih.gov/pubmed/21889315) Epilepsy Res. 2011 Dec;97(3):283-9.

Frugier, G., Coussen, F., Giraud, M.-F., Odessa, M.-F., Emerit, M.B., Boue´-Grabot, E., Garret, M., 2007. A gamma 2(R43Q) mutation, linked to epilepsy in humans, alters GABAA receptor assembly and modifies subunit composition on the cell surface. J. Biol. Chem. 282, 3819–3828.

Gibson JR, Huber KM, Südhof TC (2009). [Neuroligin-2 deletion selectively decreases inhibitory synaptic transmission originating from fast-spiking but not from somatostatin-positive interneurons.](https://www.ncbi.nlm.nih.gov/pubmed/19889999) J Neurosci. 2009 Nov 4;29(44):13883-97.

Kazdoba TM, Leach PT, Silverman JL, Crawley JN. (2014) [Modeling fragile X syndrome in the Fmr1 knockout mouse.](https://www.ncbi.nlm.nih.gov/pubmed/25606362) Intractable Rare Dis Res. 3(4):118-33.

Kyuyoung, C.L., Huguenard, J.R., 2014. Modulation of short-term plasticity in the corticothalamic circuit by group III metabotropic glutamate receptors. J. Neurosci. 34, 675–687. <http://dx.doi.org/10.1523/JNEUROSCI.1477-13.2014.>

Leung C, Cao F, Nguyen R, Joshi K, Aqrabawi AJ, Xia S, Cortez MA, Snead OC 3rd, Kim JC, Jia Z. (2018). [Activation of entorhinal cortical projections to the dentate gyrus underlies social memory retrieval.](https://www.ncbi.nlm.nih.gov/pubmed/29791849) Cell Rep. 23(8):2379-2391.

13

Liang J, Xu W, Hsu YT, Yee AX, Chen L, Südhof TC. (2015). [Conditional neuroligin-](https://www.ncbi.nlm.nih.gov/pubmed/25824299)

[2 knockout in adult medial prefrontal cortex links chronic changes in synaptic inhibition to cogni](https://www.ncbi.nlm.nih.gov/pubmed/25824299)

[tive impairments.](https://www.ncbi.nlm.nih.gov/pubmed/25824299) Mol Psychiatry. 2015 Jul;20(7):850-9

Maheshwari A, Noebels JL. (2014). [Monogenic models of absence epilepsy: windows into the complex balance between inhibition and excitation in thalamocortical microcircuits.](https://www.ncbi.nlm.nih.gov/pubmed/25194492) Prog Brain Res. 2014;213:223-52.

Meeren, H.K.M., Pijn, J.P.M., Van Luijtelaar, E.L.J.M., Coenen, A.M.L. & Lopes da Silva, F.H. Cortical focus drives widespread corticothalamic networks during spontaneous absence seizures in rats. J. Neurosci. 22, 1480–1495 (2002).

Parente DJ, Garriga C, Baskin B, Douglas G, Cho MT, Araujo GC, Shinawi M. (2017). [Neuroligin 2 nonsense variant associated with anxiety, autism, intellectual disability, hyperphagia, and obesity.](https://www.ncbi.nlm.nih.gov/pubmed/27865048) Am J Med Genet A. 2017 Jan;173(1):213-216.

Paz, J.T., Bryant, A.S., Peng, K., Fenno, L., Yizhar, O., Frankel, W.N., Deisseroth, K., Huguenard, J.R., 2011. A new mode of corticothalamic transmission revealed in the Gria4(-/-) model of absence epilepsy. Nat. Neurosci. 14, 1167–1173. [http://dx.doi. org/10.1038/nn.2896.](http://dx.doi.org/10.1038/nn.2896.)

Polack, P.O. et al. Deep layer somatosensory cortical neurons initiate spike-and-wave discharges in a genetic model of absence seizures. J. Neurosci. 27, 6590–6599 (2007).

Rudy B, Fishell G, Lee S, Hjerling-Leffler J. (2011). [Three groups of interneurons account for nearly 100% of neocortical GABAergic neurons.](https://www.ncbi.nlm.nih.gov/pubmed/21154909) Dev Neurobiol. 2011 Jan 1;71(1):45-61.

Schofield CM, Kleiman-Weiner M, Rudolph U, Huguenard JR. (2009). [A gain in GABAA receptor synaptic strength in thalamus reduces oscillatory activity and absence seizures.](https://www.ncbi.nlm.nih.gov/pubmed/19380748) Proc Natl Acad Sci U S A. 2009 May 5;106(18):7630-5.

Sudhof TC. (2008). Neuroligins and neurexins link synaptic function to cognitive disease. Nature, 2008 455(7215):903–11

Sun C, Cheng MC, Qin R, Liao DL, Chen TT, Koong FJ, Chen G, Chen CH. (2011). [Identification and functional characterization of rare mutations of the neuroligin-2 gene (NLGN2) associated with schizophrenia.](https://www.ncbi.nlm.nih.gov/pubmed/21551456)

Hum Mol Genet. 2011 Aug 1;20(15):3042-51.

Tan, H.O., Reid, C.A., Single, F.N., Davies, P.J., Chiu, C., Murphy, S., Clarke, A.L., Dibbens, L., Krestel, H., Mulley, J.C., Jones, M.V., Seeburg, P.H., Sakmann, B.,

Tremblay R, Lee S, Rudy B. (2016). [GABAergic Interneurons in the Neocortex: From Cellular Properties to Circuits.](https://www.ncbi.nlm.nih.gov/pubmed/27477017) Neuron. 2016 Jul 20;91(2):260-92.

14

**Reviewer comments, second version:**

Reviewer #1 (Remarks to the Author):

The authors have addressed most of my concerns. A few minor points remain:

1. Regarding the discrepancy between the present study and Gibson et al. 2009, the authors claim that this is due to the fact that Gibson et al. showed reductions in IPSCs from parvalbumin-positive but not somatostatin-positive interneurons. This is true (although these results were obtained using paired recordings rather than optical stimulation as stated here in the discussion in line 353); however, Gibson et al. also measured mIPSCs (Figure 2) and identified a reduction in mIPSC amplitude. Therefore, the specificity of the effect of NLG2 for PV-positive synapses does not preclude identifying changes in mIPSCs. The authors should remove this explanation and instead include an explanation of the difference between their supplementary figure 5F and Gibson et al. figure 2, which are largely identical experiments.

2. In response to my question regarding the genetic background of the animals, the authors list C57BL/6Jx129sv in the response letter but C57BL/6J only in the manuscript. Please correct this discrepancy, since this may substantially affect the behavior.

3. Excellent commercial antibodies for gephyrin and the GABA(A)Rs are available from Synaptic Systems, which work very well with methanol-fixed tissue, the same protocol that is optimal for NLG2. This may help to confirm correct localization of exogenously expressed NLG2 in future studies.


Reviewer #2 (Remarks to the Author):

The authors did not address most of my comments experimentally and simply arguing their way through and saying that those are interesting ideas more suited for followup studies. No, they are comments on the current manuscript. As such, I cannot recommend publication at this point.


Reviewer #3 (Remarks to the Author):

This revised manuscript is markedly improved. The intriguing results of association between thalamic inhibitory defects, seizures, and ASD behaviors is now much better borne out. I have only a few additional comments.

1) The EMG/seizure data is convincing on a statistical basis (fIgure S1), yet the examples provided in figure 1A don't make the point very clearly. Please add a simple EEG/EMG trace, perhaps in figure S1, that specifically delimits seizure periods and EMG silence periods to more clearly make this point.

2) It should be made more clear in the results (line 227)
that the optogenetic validation experiments were done in vitro with thalamic brain slices.

3) In the discussion (line 364), when presenting the new conceptual model of siezure generation, I suggest the phrase "feed-forward inhibition" rather than "forward inhibition"

4) There is extensive literature on GABA-B receptor contribution to absence seizures in animal models. This might be a major contributor to the seizures in NLG2 ko mice, perhaps more so than tonic inhibition as described in the discussion. If so, the seizures (and perhaps the behavior) could be rescued by systemic or thalamic GABABR blockers.

**Author rebuttal, second version:**

Reviewer #1 (Remarks to the Author):

The authors have addressed most of my concerns. A few minor points remain:

1. Regarding the discrepancy between the present study and Gibson et al. 2009, the authors claim that this is due to the fact that Gibson et al. showed reductions in IPSCs from parvalbumin-positive but not somatostatin-positive interneurons. This is true (although these results were obtained using paired recordings rather than optical stimulation as stated here in the discussion in line 353); however, Gibson et al. also measured mIPSCs (Figure 2) and identified a reduction in mIPSC amplitude. Therefore, the specificity of the effect of NLG2 for PV-positive synapses does not preclude identifying changes in mIPSCs. The authors should remove this explanation and instead include an explanation of the difference between their supplementary figure 5F and Gibson et al. figure 2, which are largely identical experiments.

**Response:** We thank this reviewer for the clarification. We have now removed our original explanation that the difference in PV- and SST-positive synapses may contribute to the difference in the cortical mIPSC results. Instead we suggested that the difference between our supplementary figure 5F and Gibson et al. figure 2, could be caused by the differences in either the age of the mice (6-18 weeks in our study vs 2-3 weeks in the Gibson et al study) and/or the cortical layers recorded (layer 5/6 in our study vs layer 4 in the Gibson et al study. We raised the possibility that the effect of the NLG2 deletion may be regulated by both neuronal types and developmental stages.

2. In response to my question regarding the genetic background of the animals, the authors list C57BL/6Jx129sv in the response letter but C57BL/6J only in the manuscript. Please correct this discrepancy, since this may substantially affect the behavior.

**Response:** The NLG KO mice were originally generated on C57BL/6J×129sv mixed background and upon arrival in the lab, these mice were backcrossed to C57Bl/6 for a few generations, therefore they are still considered to be a C57BL/6J×129sv mixed background. This information has been added to the Method section.

3. Excellent commercial antibodies for gephyrin and the GABA(A)Rs are available from Synaptic Systems, which work very well with methanol-fixed tissue, the same protocol that is optimal for NLG2. This may help to confirm correct localization of exogenously expressed NLG2 in future studies.

**Response:** We thank this reviewer for this valuable suggestion for future studies.


Reviewer #2 (Remarks to the Author):

The authors did not address most of my comments experimentally and simply arguing their way through and saying that those are interesting ideas more suited for follow up studies. No, they are comments on the current manuscript. As such, I cannot recommend publication at this point.

**Response:** We respectfully disagree with this reviewer’s comments that "authors did not address most of my comments experimentally and simply arguing their way through and saying that those are interesting ideas more suited for follow up studies". This assessment was inaccurate because we actually did numerous new experiments and analyses that are possible at this time to address this reviewer’s concerns (the original comments and my response are attached at the end of this letter).  Therefore, we have sought clarification and advice from the editor Dr. Fiona Carr. She agreed that we had addressed most of Reviewer 2 comments and there is not much more we could do experimentally. But she did suggest that the response to the Reviewer 2's original comment **1a** and **2d** could be further strengthened by doing some recordings in the PO region of the thalamus. Therefore, we have been focusing on these two points by performing following additional experiments and analyses:

Comment 1a (The recordings are said to be from the VB thalamus, but that is a big and heterogenous area. Are the cells from the VPM or PO? Does that have any bearing on the input/output function of these cells and are there systematic differences between WT and KO mice when circuit-specificity is considered?).

**Response:** All our original electrophysiological recordings were done from the ventrobasal (VPL/VPM) region because this region has been the focus of most previous studies on absence seizures . To test whether the PO region is also important, we have now performed whole cell recordings in the PO region. As shown in Supplementary Fig. 6, we found no significant differences in either sIPSCs or sEPSCs between WT and NLG2 KO mice. These results indicate that the VPL/VPM, but not PO neurons, are impaired in inhibitory synaptic transmission in the NLG2 KO mice, suggesting that NLG2 has specific effects on the ventrobasal circuit.

Comment 2d (Again, for NGL rescue in the last figure-- how much is this a VPM rescue vs PO?).

**Response:** For consistency with our recording site, our original rescue experiments were done by injecting NLG2 expressing viruses in the ventrobasal thalamic region. To test whether the PO region was also involved, we have now performed the rescue experiments by specifically injecting the NLG2 expressing viruses to the PO region of the thalamus. As shown in Supplementary Fig. 13, the expression of NLG2 in the PO cells did not rescue the SWD or motor deficits in NLG2 KO mice. These results are consistent with the electrophysiological recording data that sIPSCs were not altered in the PO neurons of the NLG2 KO mice.

Taken together, these new data suggest that the ventrobasal thalamic (VPL/VPM), but not PO neurons, are critically involved in the absence seizures in NLG2 KO mice.

Reviewer #3 (Remarks to the Author):

This revised manuscript is markedly improved. The intriguing results of association between thalamic inhibitory defects, seizures, and ASD behaviors is now much better borne out. I have only a few additional comments.

1) The EMG/seizure data is convincing on a statistical basis (figure S1), yet the examples provided in figure 1A don't make the point very clearly. Please add a simple EEG/EMG trace, perhaps in figure S1, that specifically delimits seizure periods and EMG silence periods to more clearly make this point.

**Response:** As suggested, we have now provided example traces and delimits seizure periods and EMG silence periods in Supplementary Fig. 1.

2) It should be made more clear in the results (line 227)
that the optogenetic validation experiments were done in vitro with thalamic brain slices.

**Response:** We have clarified this point in the text.

3) In the discussion (line 364), when presenting the new conceptual model of seizure generation, I suggest the phrase "feed-forward inhibition" rather than "forward inhibition"

**Response:** Thanks for this suggestion. This has been corrected.

4) There is extensive literature on GABA-B receptor contribution to absence seizures in animal models. This might be a major contributor to the seizures in NLG2 ko mice, perhaps more so than tonic inhibition as described in the discussion. If so, the seizures (and perhaps the behavior) could be rescued by systemic or thalamic GABABR blockers.

**Response:** We agree with this reviewer that it is possible that GABA-B receptors may contribute to absence seizures in NLG2 KO mice. This is an interesting area for future studies.

**Reviewer #2 Original Comments and Author Response:**

The manuscript by Cao et al. titled "Neuroligin 2 regulates absence seizures and behavioral arrests through GABAergic transmission within the thalamocortical circuitry' includes a series of experiments connecting thalamic GABAergic dysfunction with spike and wave discharges (SWDs) in the Neuroligin 2 knockout (KO) animal. The manuscript contains a variety of technical approaches, including slice electrophysiology, behavioral testing and optical manipulations. Overall, I thought the study was interesting, but some key claims are not supported by the presented data which need to be fixed. Also, the authors ought to be a bit more scholarly in acknowledging previous work that impinges on their study.

Major concerns:

1. Figures 1-3 make a good case for the authors to pursue the VB thalamus as a target for correcting SWDs in the NGL2 KO mouse. There are, however, a few issues with linking slice data to an EEG phenomenon when a mesoscopic circuit description is lacking.

a. The recordings are said to be from the VB thalamus, but that is a big and heterogenous area. Are the cells from the VPM or PO? Does that have any bearing on the input/output function of these cells and are there systematic differences between WT and KO mice when circuit-specificity is considered?

**Response:** Based on the placement of our recording electrode in the slices (Fig. 3a), most of our thalamic recordings would be from the VPL/VPM neurons. However, we do not know from which projections these synaptic responses were generated, because we did not label specific inputs. Because the nRT neurons project to both the VP and PO neurons, it is possible that both neurons are impaired in inhibitory responses in NLG2 KO mice, and in this case, we expect systematic differences in the nRT-thalamic projections between WT and KO mice. It would be interesting in future studies to determine whether all or specific inhibitory pathways to the thalamic neurons are altered in NLG2 KO mice.

b. What about bursting properties of these neurons? Are those affected in the KO?

**Response:** We have now analyzed the bursting properties of the nRT and thalamic neurons, elicited by hyperpolarizing current injections and the results showed that they were not altered in NLG2 KO mice (Supplementary Fig. 9a, b).

c. What happens to the nRT in this animal? We only see evidence for changes in IPSCs (spontaneous and miniature), but it's unclear how much of it is related to changes in upstream inhibitory circuits (nRT or otherwise).

**Response:** Yes, we also recorded IPSCs, EPSCs and firing properties from the nRT (Fig. 3, Supplementary Fig. 4 and 9a) and somatosensory cortical layer 5/6 neurons (Supplementary Fig. 5) and found no differences between WT and NLG2 KO mice.

d. Does ethosuxamide normalize these measures in the slice?

**Response:** We have now performed additional experiments and shown that ETX treatment (500 uM) did not affect the firing frequency induced by depolarizing current injections (Supplementary Fig. 9d) or sIPSCs in WT or NLG2 KO slices. However, ETX reduced the action potential number in the rebound burst firing in both genotypes (Supplementary Fig. 9b and c). These results suggest that ETX blocks the SWDs in NLG2 KO mice likely through reducing the rebound burst firing upon hyperpolarization, but not through affecting IPSCs.

2. I am not at all convinced by the connection between thalamic deficits, rescue and unifying ASD and SWD symptoms-- the main thesis that the authors are pushing. I think it's plausible, but not really shown here.

a. It is unclear to me why the authors think that ETX having an effect on their behavioral readouts suggest a link between SWDs and ASD symptoms. These can be completely independent effects exerted by ETX. Unless there is substantial work and some understanding of the underlying circuit dysfunction contributing to both pathological effects, how can one say that ETX is targeting a common mechanism? Could be that ETX has multiple independent effects. Unless the authors can rule that out, they must change their central thesis.

**Response:** Yes, we agree with this reviewer that ETX could affect SWDs and ASD independently. However, our additional data suggest that these effects are linked to each other. . First, we performed ETX experiments in WT mice and shown that it did not have any affect on locomotor activity or social recognition memory (Supplementary Fig. 6, Fig. 5c, d). Second, we have performed ETX experiments in another mouse model (PAK3 transgenic mice) that show social memory deficits but without SWDs (Leung et al., 2018), and again ETX had no effects on social memory deficits in this mouse mode (Supplementary Fig. 7). Finally, we tested the effect of ETX in the FMR1 KO mice, a widely used mouse model for syndromic ASD that show social recognition memory deficits (Kazdoba et al., 2014). The FMR1 KO mice also have increased susceptibility to seizures but different from absence seizures (Berry-Kravis, 2002). As shown in Supplementary Fig. 8, ETX also did not improve the social memory impairments in these mice. These results, together with the fact that ETX’s therapeutic effect is highly specific to absence seizures, strengthen the idea that ETX affects locomotor activity and social memory via reducing SWDs in NLG2 KO mice. However, we cannot rule out the possibility that ETX may improves these behaviors independently of SWDs and we have raised this point in the Discussion (page 18, 19). 

b. The NLG2 KO mice have a pretty specific phenotype in the three-chamber social interaction, that may have something to do with social interactions or may not. How specific is this S1/S2 effects to social contexts? Maybe the animals simply have poorer memories because of SWDs and can't remember S1 vs. this being an ASD-related social deficit? I think much more work and thought needs to be put on this experiment and its link to thalamic deficits to see if there is something compelling here.

**Response:** To determine whether NLG2 KO mice are impaired in general learning and memory we have now performed the novel objection recognition test and shown that there is no difference between WT and NLG2 KO mice (Fig. 4i-k).  These results suggest that the KO mice can recognize novelty and form memory, in a similar temporal and spatial setting. Therefore, the memory defect in NLG2 KO mice is specific to social recognition memory.

c. What are the parameters for the optogenetic 'rescue'? why do they work and what doesn't work? what can they tell us about therapeutically-viable strategies more generally?

**Response:** We only presented the data using a protocol where optical stimulation with 5 s epoch continuous light on (473 nm, 25-35 mW) and 5 s epoch light off were alternately applied for 5 min in freely moving mice during EEG recordings or behavior tests. Other protocols (e.g., alternated 10 s epoch light off and 10 s epoch light on at 5 Hz or 10 Hz for 5 min) were also tested but shown to have no effects on the SWDs or locomotor activity, therefore they were not further investigated. The reason for the latter protocols not being effective is not known, but could be related to the intrinsic neuronal activity of the nRT neurons. The fact that 5 s epoch of continuous blue-light stimulation is effective suggesting that tonic activation of the nRT neurons may be used as a therapeutic strategy to disrupt the SWDs.

d. Again, for NGL rescue in the last figure-- how much is this a VPM rescue vs. PO?

**Response:** Due to the lack of VPM and PO specific immunomarkers, we cannot be certain about the relative ratio of NLG2 expression in these neurons, but based on the expression pattern of GFP in our recorded slices, the neurons that were infected were VPL/VPM neurons. .

3. How general are NGL2 deficits in ASD? How applicable are the current findings (from a KO mouse) to ASD more generally?

**Response:** Most of gene mutations associated with ASD, including those found in the NLG2 gene, are rare mutations. However, because the NLG2 affects excitation/inhibition balance, a common mechanism targeted by many other ASD-causing gene mutations, the finding in the present study that deficits at the nRT projection to the thalamic neurons could also be applicable to other gene mutations**.**

4. Scholarly conduct: the authors do not cite previous work investigating the role of TRN in ASD, nor do the accompanying literature on TRN function in intact circuitry.

**Response:** Sorry for this oversight. We have now included description on the roles of nRT/TC in normal physiology (including slow wave sleep and sleep spindle) and in ASD in the Discussion (page 14, 15).

**Reviewer comments, third version:**

Reviewer #1 (Remarks to the Author):

The authors have addressed all of my concerns, and the manuscript has been greatly strengthened. I have no further comments.


Reviewer #3 (Remarks to the Author):

The authors have responded very well to previous critiques. I have no further issues, and consider the paper suitable for publication.

**Author rebuttal, third version:**
